# Proteomic analysis of plasma proteins of high-flux haemodialysis and on-line haemodiafiltration patients reveals differences in transthyretin levels related with anaemia

**DOI:** 10.1038/s41598-020-72104-5

**Published:** 2020-09-29

**Authors:** Emma Martínez-Alonso, Paula Alcázar, Emilio Camafeita, Milagros Fernández-Lucas, Gloria Ruíz-Roso, Alberto Alcázar

**Affiliations:** 1 Proteomics Unit, Hospital Universitario Ramón y Cajal, IRYCIS, Madrid, Spain; 2 Department of Research, Hospital Universitario Ramón y Cajal, IRYCIS, Madrid, Spain; 3 Proteomics Unit, Centro Nacional de Investigaciones Cardiovasculares Carlos III (CNIC), Madrid, Spain; 4 Centro de Investigación Biomédica en Red Enfermedades Cardiovasculares (CIBERCV), Madrid, Spain; 5 Department of Nephrology, Hospital Universitario Ramón y Cajal, IRYCIS, Madrid, Spain; 6 Department of Nephrology, Facultad de Medicina, Universidad de Alcalá, Alcalá de Henares, Spain

**Keywords:** Biotechnology, Nephrology

## Abstract

A large proportion of end-stage renal disease (ESRD) patients under long-term haemodialysis, have persistent anaemia and require high doses of recombinant human erythropoietin (rhEPO). However, the underlying mechanisms of renal anaemia have not been fully elucidated in these patients. In this study, we will be focusing on anaemia and plasma proteins in ESRD patients on high-flux haemodialysis (HF) and on-line haemodiafiltration (HDF), to investigate using two proteomic approaches if patients undergoing these treatments develop differences in their plasma protein composition and how this could be related to their anaemia. The demographic and biochemical data revealed that HDF patients had lower anaemia and much lower rhEPO requirements than HF patients. Regarding their plasma proteomes, HDF patients had increased levels of a protein highly similar to serotransferrin, trypsin-1 and immunoglobulin heavy constant chain alpha-1, and lower levels of alpha-1 antitrypsin, transthyretin, apolipoproteins E and C-III, and haptoglobin-related protein. Lower transthyretin levels in HDF patients were further confirmed by transthyretin-peptide quantification and western blot detection. Since ESRD patients have increased transthyretin, a protein that can aggregate and inhibit transferrin endocytosis and erythropoiesis, our finding that HDF patients have lower transthyretin and lower anaemia suggests that the decrease in transthyretin plasma levels would allow an increase in transferrin endocytosis, contributing to erythropoiesis. Thus, transthyretin could be a critical actor for anaemia in ESRD patients and a novel player for haemodialysis adequacy.

## Introduction

There are an increased number of end-stage renal disease (ESRD) patients requiring renal replacement treatment^1^. Haemodialysis is the main replacement treatment for patients with ESRD. It is the resource for patients waiting for a kidney transplant, or patients who are not candidates for transplantation.

In haemodialysis systems, new membranes have been developed with the aim of achieving a better renal substitute. High-flux membranes, with larger pore size, allowing a greater middle-sized molecule clearance, and better biocompatibility improve clinical outcomes^2^. The latest development is on-line haemodiafiltration that combines diffusion with elevated convection, as well as preparing the substitution fluid from water and concentrate^3^. However, at present, whether high-flux or on-line haemodiafiltration is more appropriate remains under discussion^4^.

Renal anaemia appears in most chronic renal disease patients independently of the etiology of their kidney disease^5^. It has been described that anaemia persists in 50% ESRD patients under long-term haemodialysis, despite appropriate recombinant human erythropoietin (rhEPO) administration and iron supplementation. Anaemia has a negative effect on quality of life, and it is associated with increased cardiovascular disease and decreased life expectancy^5^. However, the underlying mechanisms of renal anaemia have not yet been elucidated in dialyzed patients and renal anaemia does not completely respond to rhEPO, suggesting that there are more elements at stake.

Over the last decade, plasma proteins from patients undergoing haemodialysis have attracted greater attention. It has been reported that haemodialysis patient’s plasma proteomes are different compared with healthy human subjects^6^. Proteomic techniques enable the identification and comparison of serum proteins from patients in different haemodialysis therapies in a non-invasive way. Serum protein levels of patients after low- and high-flux haemodialysis have recently been studied, and major differences have been found^7^. A proteomic approach enables the study of the protein removal of haemodialysis membranes, assessing their ability to remove toxic metabolites as well as the undesired loss of vital proteins^8^.

In this study, we compare patients on high-flux haemodialysis treatment (HF) against patients on on-line haemodiafiltration (HDF), focusing on the proteomic study of anaemia in both groups of patients since this is an area of interest which remains to elucidate. We hypothesised that patients undergoing these therapies might develop differences in their plasma protein composition and that these might, in turn, affect the proteomic composition of their blood and have an impact on their anaemia. To address this issue, data from HF and HDF patients were collected from the database, and their plasma samples were compared by fluorescence difference in gel electrophoresis (DIGE). Differentially detected proteins were quantified and then identified by matrix-assisted laser desorption/ionization time-of-flight mass spectrometry (MALDI-TOF MS). In addition, a quantitative proteomics analysis based on liquid chromatography coupled with tandem mass spectrometry (LC–MS/MS) was performed in HDF and HF patients’ plasma samples.

## Results

### Demographic and biochemical data

The demographic and biochemical data and the treatment prescription details of the participating patients are summarized in Table 1. Patients were similar in age, gender, anthropometrical indices (height, weight and body mass index), blood pressure, albumin, creatinine, electrolytes, fasting blood sugar, lipid profile, liver function parameters, calcium-phosphate metabolism and iron reserve. The groups did not differ in dialysis vintage; and the Charlson Comorbidity Index and pre-dialytic urea values were similar. Differences between the HF and HDF patients’ groups were found for the Kt/V value^9^, having HDF patients a higher Kt/V (Table 1). Given that none of the patients had a residual renal function, the Kt/V and the Total Kt/V values within each group were the same. As expected, blood flow rate and Kt were significantly higher in HDF patients (*p* = 0.0001 and *p* = 0.0027, respectively), since on-line haemodiafiltration works with greater convection volumes. HDF patients did also have lower post-dialytic urea levels, however, this difference was not significant (*p* = 0.1559) (Table 1). Interestingly, HF patients had hyperuricemia (8.73 ± 0.47 mg/dL uric acid, > 6.8 mg/dL), whereas HDF patients had lower uric acid levels (6.78 ± 0.37 mg/dL), being this difference significant (*p* = 0.0075) (Table 1). Regarding anaemia control, the ferritin level was similar in both groups, and all HF patients required rhEPO (mean, 9,200 U/week). Conversely, anaemia control in HDF patients was much better, since only three patients required rhEPO and in lower doses (mean, 5,000 U/week). Hence, the difference in the number of patients requiring rhEPO was very significant (*p* = 0.0031) (Table 1). Despite this treatment, the haemoglobin and haematocrit of HF patients were lower than those of HDF patients (Table 1).

**Table 1:** Demographics, biochemistry and treatment prescriptions of the studied haemodialysis patients.

	Studied haemodialysis patients		*p*^a^
	High-Flux Haemodialysis (HF) (n = 10)	On-Line Haemodiafiltration (HDF) (n = 9)	HF versus HDF
**Patient demographics**			
Gender (M/F)	4/6	5/4	0.6563^b^
Age (yr)	64.00 ± 9.84	70.89 ± 15.35	0.2553
Charlson Comorbidity Index (before start HD)	6.63 ± 3.38	6.50 ± 2.39	0.9331
Cause of ESRD (no. of patients)	DM (1), GN (2), PCKD (1), nefroangiosclerosis-ischemia (1), interstitial nephritis (2), multiple myeloma (1), amyloidosis (1), hyperfiltration (1)	DM (1), GN (1), PCKD (1), nefroangiosclerosis-ischemia (1), interstitial nephritis (2), amyloidosis (1), bilateral nephrectomy (1), unknown (1)	
Dialysis vintage (months)	36.35 ± 34.68	41.61 ± 19.38	0.6930
Weight (kg)	63.05 ± 20.32	70.89 ± 12.81	0.3351
Height (m)	1.58 ± 0.09	1.60 ± 0.09	0.5833
Body mass index (kg/m2)	24.98 ± 6.41	27.53 ± 4.05	0.3206
Systolic blood pressure (mmHg), pre-dialysis	135.00 ± 33.88	127.56 ± 11.22	0.5387
Diastolic blood pressure (mmHg), pre-dialysis	70.80 ± 15.16	65.11 ± 13.86	0.4071
**Blood chemistry pre-dialysis**			
Leukocyte count (× 10^3^/µl)	7.44 ± 4.47	7.19 ± 1.85	0.8772
Haemoglobin (g/dL)	10.77 ± 2.27	12.46 ± 1.17	0.0612 *
Haematocrit (%)	32.33 ± 6.37	36.97 ± 4.27	0.0831 *
Mean corpuscular volume (fL)	96.49 ± 3.54	96.27 ± 4.77	0.9084
Ferritin (ng/ml)	458.40 ± 319.52	424.10 ± 279.26	0.8072
Albumin (g/dL)	3.28 ± 0.41	3.29 ± 0.14	0.9514
Creatinine (mg/dL)	9.78 ± 3.12	10.18 ± 2.34	0.7579
Urea (mg/dL), pre-dialysis	145.80 ± 52.68	139.78 ± 32.23	0.7707
Sodium (mM/L)	139.20 ± 2.53	137.89 ± 1.27	0.1791
Potassium (mM/L)	5.36 ± 0.77	5.78 ± 0.58	0.2025
Fasting blood sugar (mg/dL)	112.80 ± 54.42	114.56 ± 42.06	0.9388
Cholesterol (mg/dL)	187.20 ± 56.87	182.44 ± 43.69	0.8419
HDL-C (mg/dL)	38.40 ± 19.63	38.89 ± 10.47	0.9477
LDL-C (mg/dL)	116.68 ± 46.80	115.07 ± 37.48	0.9354
Triglyceride (mg/dL)	163.70 ± 73.22	141.00 ± 41.73	0.4253
Uric acid (mg/dL)	8.73 ± 1.58	6.78 ± 1.16	0.0075 **
Alanine amonitransferase (U/L)	10.70 ± 3.92	13.33 ± 5.61	0.2479
Aspartate aminotransferase (U/L)	11.40 ± 3.10	13.67 ± 4.36	0.2052
ƴ-glutamyl transferase (U/L)	35.70 ± 30.85	41.89 ± 33.57	0.6806
Alkaline phosphatase (U/L)	136.27 ± 102.76	108.44 ± 43.77	0.4626
Total bilirubin (mg/dL)	0.53 ± 0.11	0.58 ± 0.14	0.3900
Calcium (mg/dL)	8.48 ± 0.64	8.37 ± 0.68	0.7138
Phosphate (mg/dL)	4.87 ± 1.99	4.23 ± 1.36	0.4325
Calcium x phsphate (mg/dL)	40.93 ± 16.45	35.31 ± 11.20	0.4019
Intact-parathyroid hormone	367.33 ± 278.09	525.18 ± 366.40	0.3019
25(OH) Vitamin D	24.00 ± 9.57	21.42 ± 9.44	0.5629
**Blood chemistry post-dialysis**			
Urea (mg/dL), post-dialysis	41.80 ± 19.26	31.44 ± 8.50	0.1559
**HD treatment prescriptions**			
Kt/V	1.50 ± 0.22	1.75 ± 0.30	0.0532 *
Total Kt/V	1.50 ± 0.22	1.75 ± 0.30	0.0532 *
Blood flow rate (ml/min)	318.89 ± 26.19	384.44 ± 24.04	0.0001 ***
Kt	41.49 ± 6.48	50.91 ± 5.06	0.0027 **
Gained weight (kg)	3.24 ± 0.68	2.30 ± 0.32	0.2308
**Medical prescriptions**			
Nº of patients with EPO treatment/nº patients	10/10	3/9	**0.0031**^**b**^,**
rhEPO dose (U/week) in treated patients	9,200 ± 6,957	5,000 ± 1732	0.3356

Data expressed as mean SD. ^a^*p* values between HF and HDF patients were performed by t-test; ^b^*p* values by Fischer’s test. *p < 0.1, **p < 0.01 and ***p < 0.001. HD, haemodialysis; DM, diabetes mellitus; GN, glomerulonephritis; PCKD, polycystic kidney disease.

### Differential protein detection in plasma samples of HF and HDF patients

**Figure 1:**
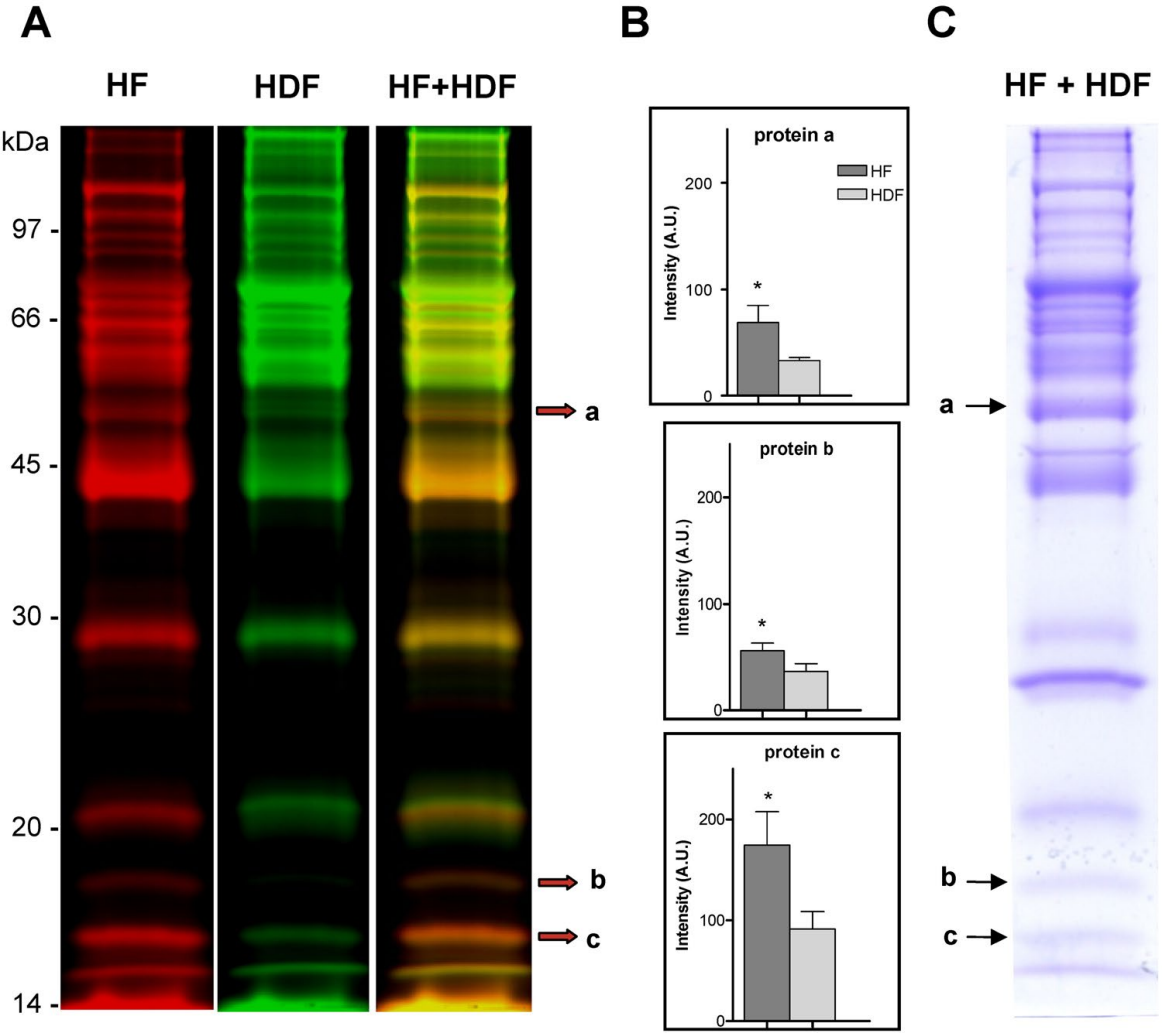
Differential protein detection of plasma samples of HF compared with HDF patients by fluorescence in gel electrophoresis (DIGE). (**A**) Albumin- and IgG-depleted plasma samples from high-flux haemodialysis (HF) and on-line haemodiafiltration (HDF) patients, were labelled with Cy5 or Cy3 fluorescent dyes. After labelling, the HF and HDF samples were combined and analysed by sodium dodecyl sulphate–polyacrylamide gel electrophoresis (SDS-PAGE) and the gel was scanned to display fluorescence-labelled proteins. For representative purpose a gel of pooled plasma samples of HF and HDF labelled with Cys is shown; proteins in the HF sample were labelled with Cy5 (red) and proteins in the HDF sample labelled with Cy3 (green). Proteins present in both HF and HDF samples were visualised in yellow due to the merge of the red and green labels (HF + HDF). The numbers on the left indicate the apparent molecular mass in kDa. The combinations of individual paired and Cy-labelled samples of HF and HDF are shown in Figure S2. (**B**) Quantification of the fluorescent proteins detected in individual HF and HDF samples from Figure S2 for differential detection. Graphs show the proteins levels (fluorescence intensity, in arbitrary units, A.U.) with significant differences in HF compared with HDF samples. Data represented as the mean of 9 independent combinations of 9 HF and 9 HDF patients. Error bars indicate SE; *p < 0.05, HF compared with HDF by t-test. The differentially detected proteins, *a* to *c*, are indicated in A by arrows with the highest labelling colour code (red, HF patients). (**C**) Gel A stained with Coomassie blue for protein staining and subsequent MALDI-TOF MS identification. Letters indicate the differentially detected proteins. Images of fluorescence labelling in A and stained image in C are a representative result and correspond to the same gel lane.

Differential protein detection of plasma samples of HF compared with HDF patients was performed by fluorescence DIGE assay. Individual and pools of HF and HDF patients’ plasma samples were depleted of albumin and IgG and labelled with fluorescent Cy3 and Cy5 dyes (Fig. 1A). Individual samples were analyzed combining each time a labelled sample from one HF patient with a corresponding labelled sample from another HDF, resulting in nine different biological replicates (Supplemental Figure S2). All detected proteins in HF and HDF patients were then quantified for differential protein detection. The results showed that HF samples had significantly higher levels than HDF samples for three proteins, named as *a*, *b* and *c* (2.1-, 1.6- and 1.9-fold, respectively) (Fig. 1B). To identify these differentially detected proteins pools of both HF and HDF samples were Cy-labelled and combined into SDS-PAGE and gels were stained with Coomassie blue (Fig. 1C) for subsequent protein digestion and identification by MALDI-TOF MS. The results of MS identification of *a* to *c* proteins are shown in Table 2.

**Table 2:** Proteins differentially detected in plasma samples of HF and HDF patients identified by MALDI-TOF MS.

Letter^a^	No.^b^	Protein	Accession no.^c^	Gene name	Theoretical mass (Da)	Score^d^	Peptides matched /searched	% Coverage	Lift^e^ (score)
a	12	Alpha-1-antitrypsin	P01009	*SERPINA1*	46,737	98	10/41	27	1641.83 (99)
b	22	Transthyretin	P02766	*TTR*	13,761	64	6/65	61	2,451.20 (189)
c	23	Haptoglobin α1	P00738	*HP*	9,192	64	10/81	19	1708.91 (91)

^a^Proteins identified by MALDI-TOF MS were named with letters according to differential protein detection in fluorescence-labelled experiments (Fig. 1), and in ^b^were named with the corresponding numbers in unlabelled and Coomassie blue-stained experiments (Fig. 2). ^c^Accession number in UniProt database (https://www.uniprot.org). ^d^Protein identification scores > 56 were significant (*p* < 0.05) in the MASCOT database search algorithm. ^e^MALDI LIFT-TOF/TOF MS identification mode; the m/z of the fragmented parental peptide is indicated; MASCOT scores (in parenthesis) > 28 were significant (*p* < 0.05).

**Figure 2:**
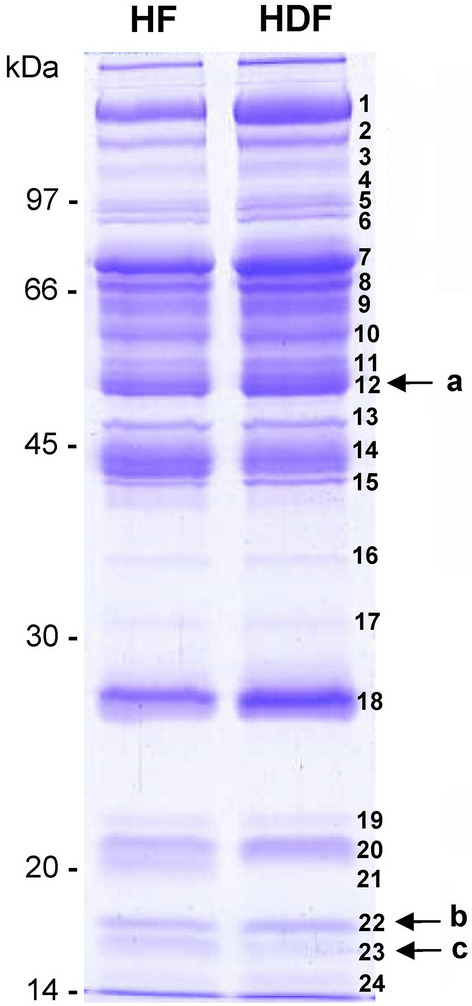
SDS-PAGE of plasma samples from HF and HDF patients for protein detection and MS identification. Gel stained with Coomassie blue shows the stained proteins in albumin/IgG-depleted plasma pools from HF and HDF samples. Detected proteins are indicated with numbers (bands 1–24) and were processed for identification by MALDI-TOF MS. Letters indicate the proteins differentially detected and shown in Fig. 1. The numbers on the left indicate the apparent molecular mass in kDa. The figure shows representative HF and HDF samples ran in the same stained gel.

In order to confirm the identification of the proteins *a* to *c*, unlabelled samples depleted of albumin and IgG from each pool of HF and HDF patients’ plasma were run into SDS-PAGE and the gels were stained with Coomassie blue (Fig. 2). The stained gels allowed the detection of 24 different proteins (bands) (Fig. 2), which were then excised from the gel for later protein digestion and identification by MALDI-TOF MS. Differentially detected proteins in HF and HDF patients’ plasma samples in fluorescence-labelled experiments, proteins *a* to *c* (Fig. 1), corresponding in unlabelled experiments to proteins 12, 22 and 23 (Fig. 2), respectively, and were identified as the exact same proteins, results that are shown in Table 2. Other proteins identified in plasma samples from HF and HDF patients are shown in Supplementary Table S1. The MALDI-TOF MS spectra and the identifications done with the database searches are shown in Supplementary Material. In summary, we found that HF patients showed higher levels of α-1-antitrypsin (protein 12), transthyretin (protein 22) and haptoglobin α1 (protein 23).

### Detection of low mass proteins in plasma samples of HF and HDF patients

**Figure 3:**
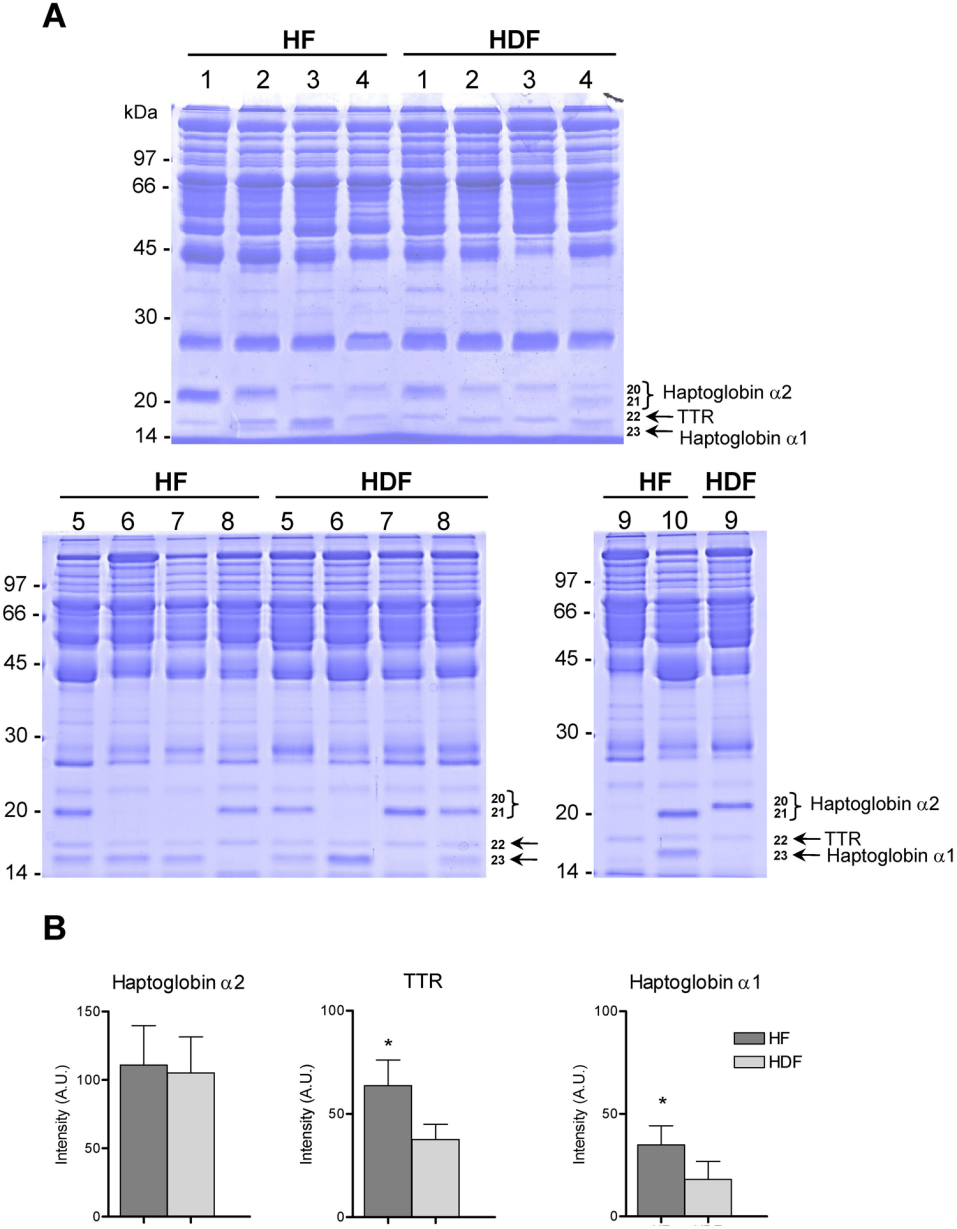
Transthyretin detection in individual HF and HDF patients’ plasma samples. (**A**) Individual albumin- and IgG-depleted plasma samples from each HF and HDF patient were analysed independently by SDS-PAGE and stained with Coomassie blue. Proteins haptoglobin α2 (20 and 21), transthyretin (22) and haptoglobin α1 (23) were identified by MALDI-TOF MS. The figure shows stained whole gels corresponding to HF and HDF patients. The numbers on the left indicate the apparent molecular mass in kDa. (**B**) Quantification of haptoglobin α2, transthyretin (TTR), and haptoglobin α1 (arbitrary units, A.U.). Graphs show the proteins levels in HF and HDF samples represented as the mean of the 10 and 9 samples, respectively. Error bars indicate SE; *p < 0.05, HF compared with HDF by t-test.

Interestingly, haptoglobin α2, transthyretin and haptoglobin α1, are proteins with a low mass that were detected in lower levels in HDF patients’ samples and could be cleared by the more efficient haemodiafiltration process. Therefore, we decided to study these proteins in unlabelled samples to characterize these differences. Individual albumin/IgG-depleted plasma samples from each of the ten HF and nine HDF patients were analysed independently by SDS-PAGE and stained with Coomassie blue (Fig. 3A). Proteins 20 to 23 were quantified and after protein digestion identified by MALDI-TOF MS and confirmed as expected as haptoglobin α2 (proteins 20 and 21), transthyretin (protein 22) and haptoglobin α1 (protein 23) (Fig. 3A). Since proteins 20 and 21 were both identified as haptoglobin α2, they were quantified together and showed similar intensity values in HF and HDF patient samples (Fig. 3B). This result was consistent with the absence of differences found for this protein between HF and HDF groups in the differential protein detection experiments (Fig. 1B). The changes for haptoglobin α2, transthyretin and haptoglobin α1 were 1.1-, 1.7- and 1.9-fold, respectively (Fig. 3B). Differences in haptoglobin α1 were significant (Fig. 1B, protein *c*; and Fig. 3B), but after further study, this was found to be a result of an imbalance in the allele distribution between the groups (*Hp1* or *Hp2*), and not due to a difference in protein levels (see Supplementary Material). In summary, transthyretin (TTR) showed the most significant difference in protein levels between HF and HDF patients’ groups (Fig. 3B), finding that was consistent with the result shown in Fig. 1B (protein *b*).

### LC–MS/MS analysis of plasma samples of HF and HDF patients

To assess additional proteins with protein abundance changes in HDF patients as compared with HF patients, we performed a quantitative proteomics analysis based on LC–MS/MS. Plasma samples from nine patients per HF and HDF group (the same patients as in DIGE analysis) were digested with trypsin and the resulting tryptic peptides were labelled with isobaric tags. After LC–MS/MS analysis, the proteins identified in HF and HDF patients were subjected to statistical analysis to determine significant changes in protein abundance in HDF samples compared with the HF as control group (Table 3). Only those proteins identified with more than one peptide and detected across all 18 samples were considered, which resulted in 233 proteins quantified (Supplementary Table S2). Of these, 3 were found to be increased in HDF patients (Zq ≥ 2.0, *p* ≤ 0.05) and 7 decreased (Zq ≤ −2.0, *p* ≤ 0.05) (Table 3). Among the proteins found increased in HDF patient, trypsin-1, immunoglobulin heavy constant chain α-1 and a protein highly similar to serotransferrin were identified. The latter protein that was also found significantly increased according to the more strict FDRq-based criterion (FDRq < 0.05, see Methods). Seven proteins were detected with lower levels in HDF patients –i.e., with higher levels in HF patients– which included apolipoproteins E and C-III and haptoglobin-related protein, the latter also significantly increased according to the more restrictive FDR-based criterion (FDRq < 0.05) (Table 3). The other four proteins were uncharacterized proteins (Table 3). Interestingly, the eighth protein with lower levels in HDF patients corresponded to transthyretin, with a quantification value (Zq = −1.97, Table S2 and Table 3) very closely to the limit of statistical significance (Zq ≤ −2.0, *p* ≤ 0.05). Complete information of the proteins quantified in LC–MS/MS-based study is listed in Supplementary Table S2.

**Table 3:**
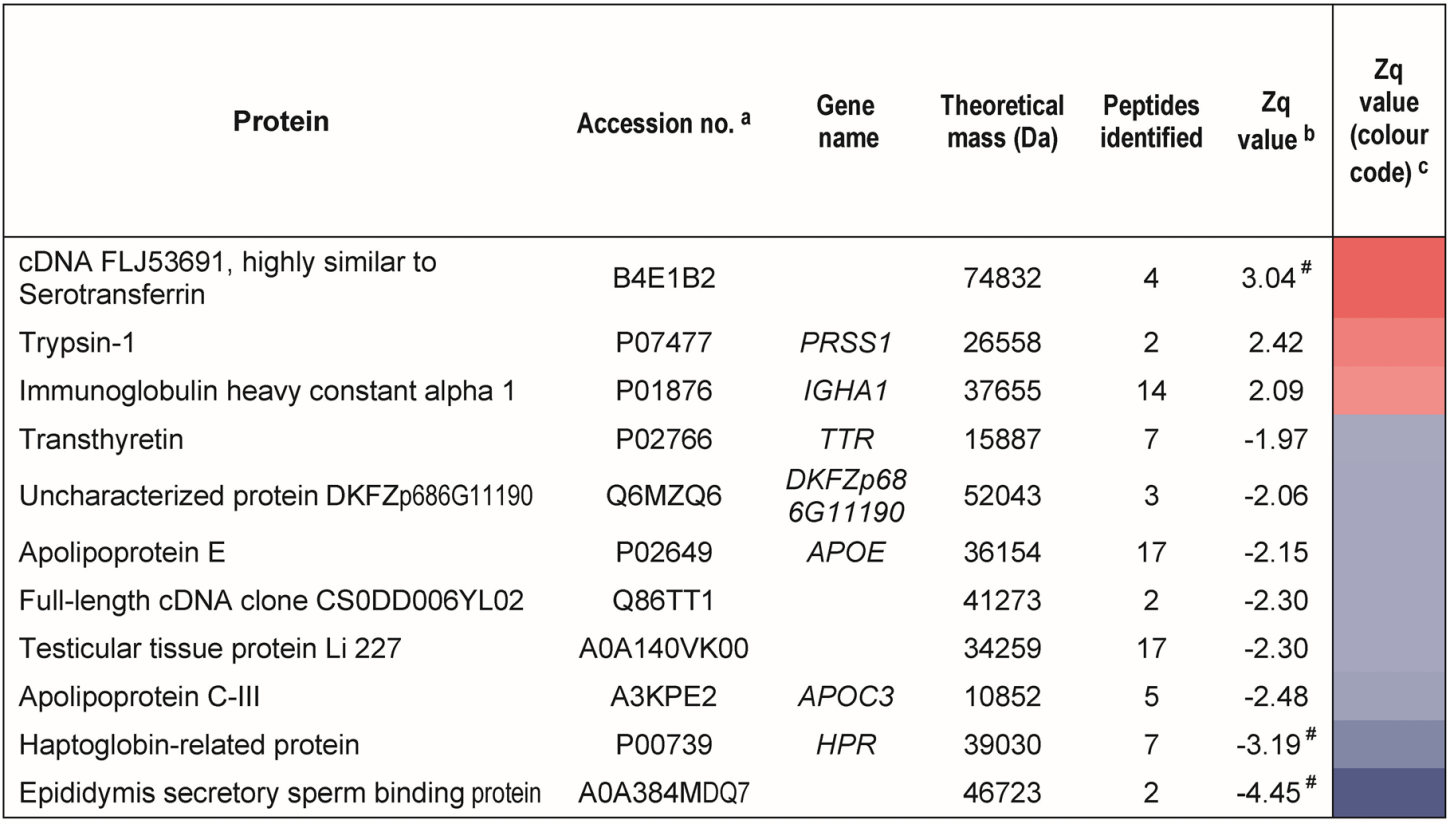
Proteins with abundance changes in plasma samples of HDF compared with HF patients in LC–MS/MS analysis.

^a^Accession number in UniProt database (https://www.uniprot.org). ^b^Protein quantification values (Zq) are normalized log2-ratios expressed in standard deviation units: Zq > 0 and Zq < 0 indicate increased or decreased, respectively, protein abundance in HDF compared with HF patients. ^c^Zq values in a colour scale; red and blue represent increased or decreased levels, respectively, in HDF compared with HF. Zq ≥ 2, and ≤ −2, were significant (*p* ≤ 0.05); ^#^significant FDRq value (< 0.05).

Haemodiafiltration provides a greater removal of middle mass molecules (0.5–40 kDa)^10^ and could be doing a more efficient clearance of plasma in HDF patients. Thus, the identification of low-middle molecular mass proteins (≤ 40 kDa) in plasma samples from HF and HDF patients resulted in 115 proteins (Table S2). Of these, 75 showed lower levels in HDF patients, including transthyretin, apolipoproteins E and C-III and haptoglobin-related protein, whilst 40 showed higher levels, with trypsin-1 and immunoglobulin heavy constant chain α-1 (Table 3). In summary, a total of 233 proteins were identified and quantified in plasma samples from HF and HDF patients in the LC–MS/MS analysis. We found that HDF patients showed higher levels of trypsin-1, immunoglobulin heavy constant chain α-1 and a protein highly similar to serotransferrin. Whereas, HF patients showed higher levels of apolipoproteins E and C-III, haptoglobin-related protein and transthyretin, confirming the results described above for this last protein.

### Transthyretin peptide quantification in patients’ plasma samples

**Figure 4:**
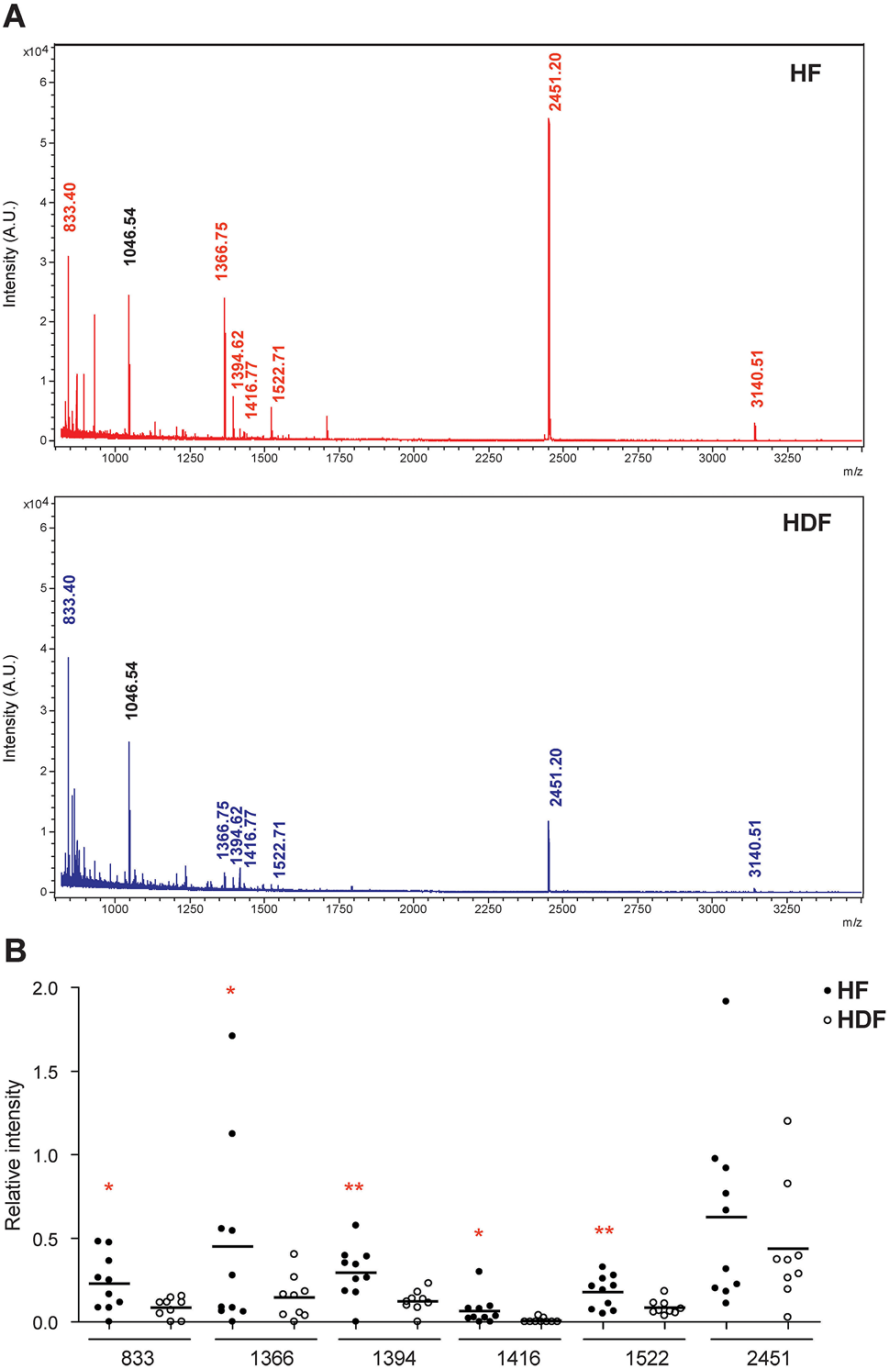
Mass spectra and quantification of transthyretin (TTR) peptides of HF and HDF paients by mass spectrometry. (**A**) Mass spectra of the TTR peptides from trypsin digestion obtained by MALDI-TOF MS. The spectra show the m/z peaks corresponding to human TTR peptides from HF (red) and HDF (blue) samples and the angiotensin-II peptide, added as internal standard (black). Peptide intensities in arbitrary units (A.U.); m/z values of MH^+^ peaks of TTR peptides and angiotensin-II are indicated. (**B**) TTR peptides, with MH^+^ peaks at m/z 833.40, 1,366.75, 1,394.62, 1,416.77, 1522.71 and 2,451.20 were quantified with respect to the angiotensin-II peptide (internal standard) as relative intensities (ratio TTR peptide intensity/angiotensin-II intensity). The graph shows the quantified levels of each TTR peptide for individual HF and HDF samples. Data represent individual values and horizontal lines represent the mean. *p < 0.05 and **p < 0.01, HF group compared with HDF group by t-test.

To confirm these differences found in the protein levels of TTR between the patients from both groups, TTR from individual HF and HDF patients’ plasma samples (Fig. 3A, protein 22) was excised, processed for trypsin digestion and TTR peptides quantified in the MALDI-TOF MS spectra (Fig. 4A). TTR peptides from MALDI-TOF MS spectra were quantified in the mass rage of the calibration experiments (TTR peptides at m/z from 833.40 to 2,451.20, Fig. 4B) as relative intensities (ratio TTR peptide intensity/angiotensin-II intensity, see Supplementary Material). The quantified levels of each TTR peptide for individual HF and HDF samples are shown in Fig. 4B. HF patients had a significantly increased relative intensity of TTR in five of the six TTR peptides quantified. This result confirmed that HF patients had an increased level of TTR.

### Transthyretin levels in patients’ plasma samples

**Figure 5:**
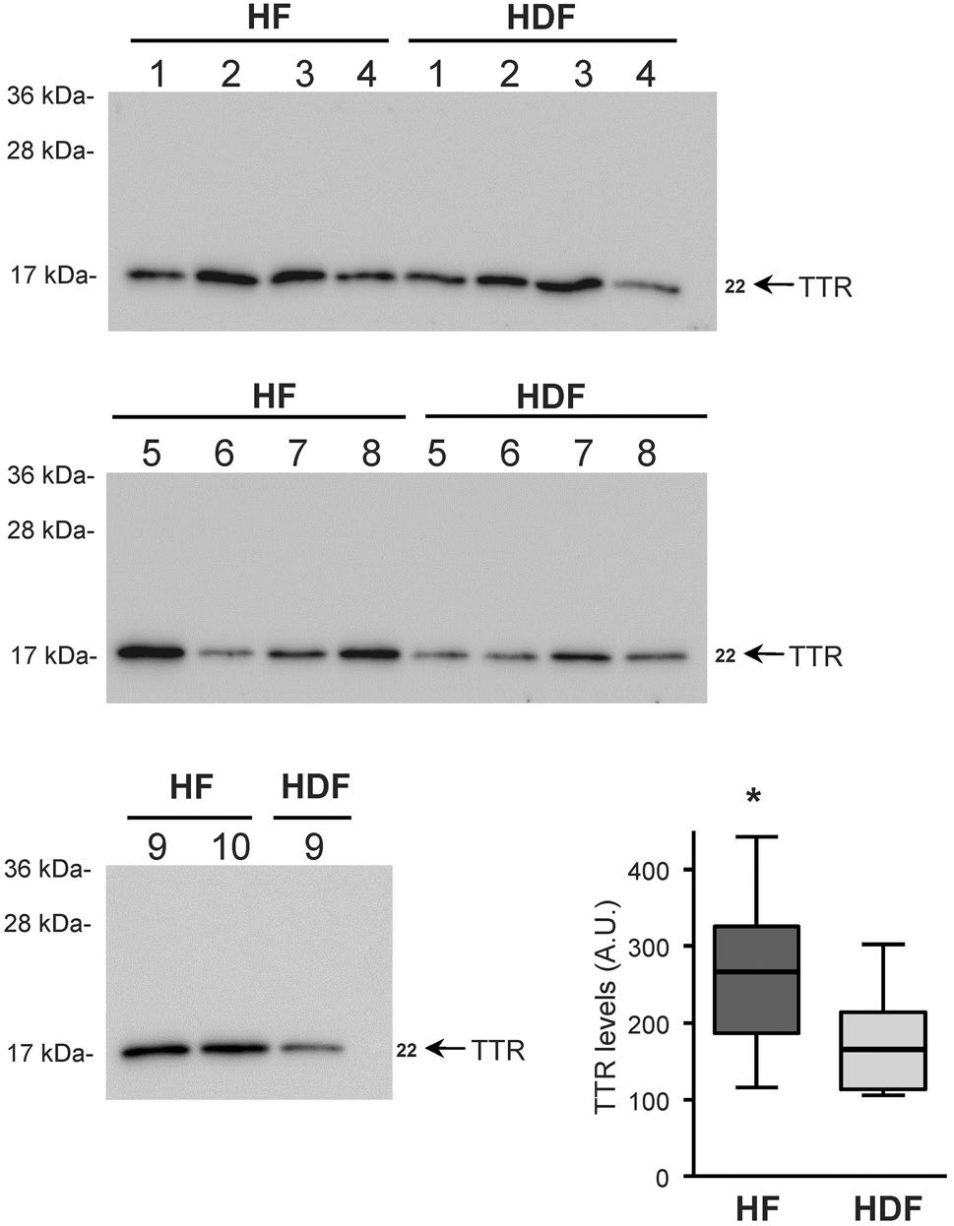
Transthyretin (TTR) detection by western blot in HF and HDF plasma samples. Individual albumin- and IgG-depleted plasma samples of each HF and HDF patient were analysed independently by western blotting with anti-TTR antibody (images). In the images, arrows show the TTR protein detected, and the numbers on the left indicate the apparent molecular mass from standards. The box graph show the quantification of the TTR levels (arbitrary units, A.U.) detected in HF and HDF samples represented as the mean of the 10 and 9 samples, respectively, (thick line) ± 25% and 75% percentile (box) and the minimum and maximum TTR level (whiskers). *p < 0.05, HF compared with HDF by t-test. Images show whole blots. Blotted proteins were staining with Fast Green as loading control of the analyzed samples, and used for normalization of detected TTR levels (see Figure S5 in the Supplementary Material).

Finally, differences in TTR protein levels between HF and HDF patients were detected and confirmed by western blot analysis. Individual albumin/IgG-depleted plasma samples were analysed independently by SDS-PAGE, blotted on PVDF membrane and after the membranes incubated with a specific anti-TTR antibody to TTR detection (Fig. 5). TTR signal was quantified in each patient and the results showed that HF patients had significantly higher levels of TTR (1.6-fold) compared with HDF patients (Fig. 5, box graph).

## Discussion

In our study, HDF patients had higher Kt/V, as reported by a recent study^10^. The Kt/V is the intradialytic urea-reduction ratio, and it is used as a measure of haemodialysis efficacy. The increased Kt/V correlated with increased clearance of urea (post-dialysis urea) by the dialyzer, although, this difference was not significant.

Interestingly, HDF patients showed significant lower uric acid levels compared with HF patients, a finding which has not been previously reported (Table 1). On-line haemodiafiltration does provide a greater clearing per unit surface area of small and middle molecules and high convection volume, and uric acid is a small (168 Da) and very water-soluble molecule, so this finding is coherent.

Haemoglobin and haematocrit were lower in HF patients than in HDF patients (Table 1). Moreover, the anaemia control in HDF patients was good and only three of the patients required rhEPO, and at low doses. These differences cannot be attributed to other causes of anaemia since there were no differences in the mean corpuscular volume nor in the ferritin levels (Table 1), and both groups were being supplemented with folic acid and iron. Additionally, liver function parameters were similar. Consequently, our results demonstrated that on-line haemodiafiltration improves anaemia control with reduced erythropoietin doses. This finding was also described by Maduell et al.^11^, who hypothesised that these differences may be due to the increased dialysis doses of on-line haemodiafiltration and to the greater elimination of middle-sized molecules.

Protein profiles in ESRD patients are altered by the haemodialysis process in a very notable way^6^. When studying and comparing the effect of these two haemodialysis techniques combining two complementary approaches of quantitative proteomics, we have found proteome differences which had not been described before. In the differential protein detection by fluorescence DIGE, α-1-antitrypsin and TTR showed significantly increased protein levels in HF samples compared with HDF (Fig. 1, Table 2). In the LC–MS/MS study, HDF patients showed higher levels of a protein highly similar to serotransferrin, trypsin-1 and immunoglobulin heavy constant chain α-1. In contrast, HF patients showed higher levels of apolipoproteins E and C-III, haptoglobin-related protein and TTR (Table 3). The α-1-antitrypsin protein (a trypsin inhibitor) was identified in LC–MS/MS study and, although here the change in protein abundance was in the same direction than in DIGE analysis, it showed no significant changes between the HF and HDF groups. The TTR identified in the LC–MS/MS analysis showed protein abundance changes in the same direction as those found in differential protein detection in DIGE analysis (Fig. 1, Table 2), supporting the results of the TTR identified in both studies. Hence, with isobaric labelling and LC–MS/MS analysis additional differential proteins were identified, which clearly shows that this approach is a powerful technique compared to differential protein detection by 1-D DIGE, although both approaches can be complementary to determine protein of physiological interest.

TTR result was confirmed comparing TTR levels in HF and HDF patients’ plasma samples in the stained gel-based approach (Fig. 3). Moreover, we found that HF patients had increased levels in 5 of the 6 TTR peptides by MS quantification (Fig. 4) and had increased levels of TTR detected by western blot and later quantification (Fig. 5). Thus, the fold change for TTR in plasma samples between HF and HDF patients was 1.6-, 1.7- and 1.6-fold according to the results of Figs. 1, 3 and 5, respectively, all of which data that were in agreement. Herein, DIGE data and western blot quantification yield the exact fold change for TTR in HF samples compared with HDF (1.6-fold) showing the strength of both approaches.

Regarding the identification of low-middle molecular mass proteins in plasma samples in HDF patients that could be cleared by the more efficient haemodiafiltration process^10^, the LC–MS/MS analysis found 115 proteins of which 75 showed lower levels in HDF, including five of the eight proteins with lower levels in HDF compared with HF group. The quantification of these 115 proteins by isobaric labelling in the LC–MS/MS-based study showed a significant correlation against their theoretical molecular mass (*p* < 0.0043, Pearson test), being the middle mass proteins more commonly decreased in HDF patients (Figure S6). This result could be confirming a more efficient haemodiafiltration process on middle molecular mass proteins.

Transthyretin (or prealbumin), is synthesised by the choroid plexus of the brain and by the liver and catabolised in the liver and in the kidneys. TTR is known for transporting thyroid hormones and vitamin A (by forming the retinol transport complex with retinol-binding protein (RBP)-4) in blood and cerebrospinal fluid. Interestingly, ESRD patients have increased TTR serum concentrations compared with healthy subjects^12^, however, the role of TTR in anaemia has not been studied before.

Anaemia in ESRD patients has been attributed to inflammation, partly because a uremic milieu promotes an inflammatory response, which would inhibit EPO synthesis^5^. Anaemia of inflammation is thought to be a result of iron sequestration leading to defective erythropoiesis, due to the inhibiting effect of proinflammatory cytokines^13^. Following this, it has been described that proinflammatory cytokines increase hepcidin expression decreasing duodenal iron absorption^14^. However, this does not explain why ESRD patients might not be able to use their iron stores effectively, e.g., with iron supplementation treatment, and therefore have anaemia^15^.

Transferrin-bound iron is the only source of iron for erythroid precursors, required for haemoglobin synthesis and maturation in the bone marrow. If iron is not delivered into erythroid precursors, erythroid maturation is halted, leading to anaemia. A recent study has shown that aggregated TTR inhibits transferrin endocytosis^16^. Notably, this inhibition was reversible because the removal of the aggregated TTR restored normal transferrin endocytic activity in cells^16^. Related to this interesting finding, it is known that normal TTR can induce TTR aggregates^16,17^.

Our results show that HDF patients have decreased TTR levels and a reduced incidence of anaemia and rhEPO requirements. Given that on-line haemodiafiltration provides an increased clearance of middle-sized molecules (0.5–40 kDa), it can increase the removal of in excess TTR –which could aggregate–, hence increasing transferrin endocytosis and favouring erythropoiesis. Thus, our suggested mechanism for anaemia in ESRD patients is that an increased level of TTR could induce TTR aggregates, inhibiting transferrin endocytosis, this, in turn, decreases intracellular iron, reducing erythropoiesis and leading to anaemia.

HDF patients have significantly lower rhEPO requirements and have lower TTR levelsand increased a protein highly similar to serotransferrin. Serotransferrin is considered a marker of erythropoiesis^18–20^ and this result would indicate that these patients are carrying out more erythropoiesis. These findings are of clinical relevance and future studies should be conducted, exploring further into the beneficial role of on-line haemodiafiltration in ESRD patients. Thus, TTR could be a critical actor for anaemia in ESRD patients and could also be a novel biomarker for haemodialysis adequacy.

## Methods

### Subjects

The patients for the study were selected randomly from the list of patients that were going to attend their usual high-flux haemodialysis or on-line haemodiafiltration session on the same day, and were named as HF or HDF patients, respectively. Following the standard procedure, blood samples were drawn prior to the start of haemodialysis procedure for follow-up and clinical control of patients, and coded and used for this study. We performed a power analysis (https://www.biomath.info/power/ttest.htm) to determine sample size. We chose the significant level at 0.05 and the power set at 0.8 (80%), and the sample size obtained was 9 subjects per group. In this way, blood samples from ten HF and HDF patients were studied. One HDF patient revoked later the informed consent and removed from the study, with the HDF group having nine patients.

Data concerning the type of dialysis, their kidney disease, and their demographic status was collected from the haemodialysis database for these patients, preserving their anonymity and analysed blindly. Data from patients’ samples were independently analyzed and treatment information was blindly performed throughout the study. Informed consent was obtained from all subjects and the study was approved by the Research Ethical Committee from the Hospital Universitario Ramón y Cajal (Madrid, Spain) and according to the Declaration of Helsinki. The description of high-flux haemodialysis and on-line haemodiafiltration and the baseline characteristics of the studied patients are included in the Supplementary Material.

### Plasma samples

Blood samples from HF and HDF patients were analysed following the standard clinical laboratory methods. Pre-dialysis concentrations of electrolytes, urea and creatinine, along with the haemogram, the lipid profile, the liver function indices and the calcium-phosphate metabolic parameters, were determined by routine clinical laboratory methods. Urea concentration was measured before and after treatment. Plasma samples were obtained and albumin and IgG removed to improve the resolution of in-gel proteomic analysis and were analysed blindly at the proteomic laboratory. Detailed protocol of plasma sample processing is included in Supplementary Material.

### Gel electrophoresis and fluorescence labelling

Plasma samples from HF and HDF patients (15 μg of protein) were separated and analysed by denaturating sodium dodecyl sulphate–polyacrylamide gel electrophoresis (SDS-PAGE) of 12% acrylamide (3% cross-linking). Low molecular weight calibration kit (GE Healthcare) for SDS-PAGE was used as molecular mass standards.

In DIGE experiments, plasma samples from HF and HDF patients (5 μg of protein) were labelled with Cy3 or Cy5 fluorescent dyes (GE Healthcare) according to the standard protocol for DIGE assay, as previously described^21^. Labelling was performed in samples from HF and HDF patients (n = 9 per group) in a paired combination. A sample of HF (patient 1) was randomly discarded and the labelling was alternated between Cy3 and Cy5 in each group. After fluorescence labelling, HF and HDF samples were combined and analysed by SDS-PAGE as described above. In the Cy-labelled experiments for subsequent MALDI-TOF MS identification pools (n = 5) of both HF and HDF patients’ plasma samples were combined. A detailed protocol is included in the Supplementary Material.

Coomassie brilliant blue (R-250, BioRad) was used to stain the proteins in gels, that after were washed-out with 20% ethanol/7% acetic acid (vol/vol). Finally, gels were preserved in 10% ethanol until scanning and protein quantification, or extraction for subsequent identification by MS.

### In-gel protein digestion and protein identification by mass spectrometry

Gel bands were excised manually from the Coomassie blue-stained gels for tryptic digestion with modified porcine trypsin (Promega). The digestion protocol was according to Shevchenko et al., with minor variations, as previously described^21,22^. After digestion, peptide mass fingerprinting (PMF) was analyzed by MALDI-TOF MS (Autoflex III, Bruker-Daltonics) for protein identification, as previously described^21^. Additionally, when available and for confirmation of protein identity, peptide fragmentation was performed by MS in tandem MALDI LIFT-TOF/TOF^23^. Detailed protocol is included in the Supplementary Material. Additionally, results of protein searches and spectra in the case of MALDI-TOF MS- and LIFT-TOF/TOF-based protein identifications are included in the Supplementary Material Sects. 16 and 17, respectively.

### Protein quantification

The fluorescence-labelled proteins in DIGE experiments were detected scanning the gels using a Typhoon 9,200 imager (GE Healthcare). Differential protein detection between HF and HDF samples was quantified in the scanned images using the Quantity One software package (Bio-Rad). Proteins stained with Coomassie blue were scanned and quantified using an image analyser equipped with the Quantity One software. The data obtained were processed using Microsoft Excel spreadsheet and Prism statistical package (GraphPad Software).

### LC–MS/MS analysis

Plasma samples (6 μl, without albumin/IgG depletion) were boiled for 5 min at 100 °C in the presence of 25 mM Tris–HCl pH 6.8, 1% SDS and 50 mM DTT, diluted with denaturing buffer (8 M urea in 100 mM Tris–HCl pH 8.5) and after washing, samples were digested overnight at 37 °C with sequencing grade trypsin (Promega). The resulting tryptic peptides from each sample were recovered and their concentration was determined using a Direct Detect IR spectrometer (Millipore). Equal amounts of each peptide sample were labelled with isobaric 10-plex tandem mass tags (TMT) (Thermo Fisher Scientific) according to the manufacturer’s instructions and mixed together. An internal control was prepared by pooling the nine HF samples (control group) and was used as a reference to express relative quantification values. A detailed protocol is included in the Supplementary Material.

For peptide identification, labelled tryptic peptide samples were applied to an EASY-nLC 1,000 nano-flow HPLC system (Thermo Fisher Scientific) coupled on-line with an orbitrap Fusion mass spectrometer (Thermo Fisher Scientific). C18-based reverse phase separation was used with a 2-cm trap column and a 50-cm analytical column (EASY-Spray, Thermo Fisher Scientific). Mass spectra were acquired and LC–MS/MS data were analyzed with Proteome Discoverer (version 2.1, Thermo Fisher Scientific) using SEQUEST-HT (Thermo Fisher Scientific). Peptide identification from MS/MS data was performed using the probability ratio method^24^. Peptides were assigned only to the best protein proposed by the Proteome Discoverer algorithm. A detailed protocol is included in the Supplementary Material.

### Quantification at the peptide and protein levels in LC–MS/MS analysis

The quantitative information extracted from the MS/MS spectra by Proteome Discoverer was integrated from the spectrum level to the peptide level and then to the protein level on the basis of the WSPP model^25^ and the systems biology triangle algorithm^26^ using the SanXoT software package^27^. A log2-ratio of every scan was calculated using the TMT reporter ion intensities coming from samples and the reference. Thus, the quantification of each peptide and protein was calculated as the weighted average of its scans or peptides, respectively, and expressed in standard deviation units as Zq value. A threshold of Zq ≥ 2, or ≤ −2, was considered significant (*p* ≤ 0.05)^25^. Additional information is included in the Supplementary Material.

### Quantification of TTR peptides by mass spectrometry

After in-gel protein digestion, angiotensin-II peptide (4 fmol) was added to collected tryptic peptides. Peptides obtained from the digestion and the angiotensin-II peptide were analysed by MALDI-TOF MS, and peptide intensities in the MS spectra were quantified with respect to the angiotensin-II peptide intensity as a ratio of the relative intensities peptide/angiotensin-II. Calibration experiments carried out to test the accuracy of this quantification are included in the Supplementary Material.

### Western blot analysis

Albumin- and IgG-depleted plasma samples from HF and HDF patients (5 μg of protein) were analysed by SDS-PAGE as describe above, and transferred onto PVDF membranes (GE Healthcare). The membranes were incubated overnight at 4 °C with rabbit polyclonal anti-TTR antibody (ab78548, Abcam), washed, then incubated for 1 h with peroxidase-conjugated anti-rabbit IgG (GE Healthcare), and developed with Clarity reagent (Bio-Rad). TTR protein was detected scanning the membranes using a ChemiDoc MP imager (Bio-Rad) and quantified using the Quantity One software (Bio-Rad). In all experiments, blotted proteins were staining with Fast Green (Bio-Rad) in the PVDF membranes as loading control of the samples.

### Data collection and statistical analyses

The patients’ data from the haemodialysis database was collected and analysed blindly. To compare and determine differences between the groups, statistical analysis was performed using Microsoft Excel spreadsheets and the Prism statistical package (GraphPad Software). Data are presented in arbitrary units (A.U.) unless specified, and expressed as mean ± SD for the nineteen patients. Comparisons were done by the Student’s t test for continuous variables and Fisher's test for categorical variables. Pearson test was used to calculate linear correlations.

## Supplementary information

Supplementary information.
