# Supplementary material for: Proteomic analysis of plasma proteins of high-flux haemodialysis and on-line haemodiafiltration patients reveals differences in transthyretin levels related with anaemia
Source: Sci Rep. 2020 Sep 29;10:16029. doi: 10.1038/s41598-020-72104-5 (PMC7524835; doi:10.1038/s41598-020-72104-5)

SUPPLEMENTARY MATERIAL

Proteomic analysis of plasma proteins of high-flux haemodialysis and on-line haemodiafiltration patients reveals differences in transthyretin levels related with anaemia

Emma Martínez-Alonso,[1,2] Paula Alcázar,[1] Emilio Camafeita,[3,4] Milagros Fernández-Lucas,[5,6] Gloria Ruíz-Roso,[5] and Alberto Alcázar [1,2*]

[1] Proteomics Unit. Hospital Universitario Ramón y Cajal, IRYCIS, Madrid, Spain

[2] Department of Research, Hospital Universitario Ramón y Cajal, IRYCIS, Madrid, Spain

[3] Proteomics Unit. Centro Nacional de Investigaciones Cardiovasculares Carlos III (CNIC), Madrid, Spain

[4] Centro de Investigación Biomédica en Red Enfermedades Cardiovasculares (CIBERCV), Spain

[5] Department of Nephrology, Hospital Universitario Ramón y Cajal, IRYCIS, Madrid, Spain

[6] Department of Nephrology, Facultad de Medicina, Universidad de Alcalá, Alcal§ de Henares, Spain

Table of Contents:

1. High-flux haemodialysis and on-line haemodiafiltration…………………………......S2
2. Baseline characteristics of the patients……………………………………………...S3
3. Preparation of plasma samples………………………………………………………S3
4. 1-D DIGE analysis..………………..…………………...…………………………...S4
    4.1. Fluorescence labelling. DIGE assay..…….…………………….…………..S4
    4.2. In-gel protein digestion……………………….………………………....S4
    4.3. Protein identification by mass spectrometry……………………..……........S5
5. LC-MS/MS Analysis………………………………………………………...………S5
    5.1 Protein digestion………………………………………………………………S5
    5.2. Isobaric labelling of peptides using tandem tass tags (TMT)……………....S6
    5.3. Peptide identification……………………………………………………….…S6
    5.4. Quantification at the peptide and protein levels…..………………………..S7
6. Quantification of TTR peptides by mass spectrometry. Calibration experiments…...S8

7. Detection of the allele distribution of haptoglobin α in HF and HDF patients………S9
8. Supplementary Table S1……………………………………………………..……....S10
9. Supplementary Table S2……………………………………………………..……....S11
10. Supplementary Figure S1………………………………………………..……....…..S15
11. Supplementary Figure S2………………………………………………….……..….S16
12. Supplementary Figure S3………………………………………………….……..….S17
13. Supplementary Figure S4……………………………………………...………....S19
14. Supplementary Figure S5……………………………………………………………S20
15. Supplementary Figure S6……………………………………………………………S21
16. MALDI-TOF MS spectra and Mascot searches……………………………….…….S22
17. MALDI LIF-TOF/TOF MS spectra and Mascot searches…………………….….S52

1. High-flux haemodialysis and on-line haemodiafiltration

Haemodialysis was prescribed as four-hour dialysis sessions trice weekly, with a blood flow rate between 220 and 400 ml/min. High-flux haemodialysis was performed using dialysis membranes of a blend of polyarylethersulfone and polyvinylpyrrolidone (Revaclear), and polyamide (Polyflux-210H). On-line haemodiafiltration was performed using polysulfone membranes (Helixone). Other aspects of the patient's treatment prescription did not differ between the two groups. All patients had not residual renal function and were on dialysis for more than 6 months. The treatment times and blood flow rates (individualized for each patient) remained unchanged from their usual prescription. To achieve anticoagulation, heparin was used (firstly a loading dose and then a constant infusion of heparin). Net fluid removal was also individualized. The efficacy of haemodialysis was indexed by the urea kinetic equation single-pool variable volume Kt/V according to Daugirdas [1]. Dialysis vintage years were similar for both groups. Residual renal function was negligible. Kt/V represents the fractional clearance of urea, and it is used as an index of the dialysis dose (K: rate of urea clearance by the dialyzer in ml/min, t: duration of treatment session in min, V: urea volume distribution in the patient in ml). Both groups met the recommendations from the National Kidney Foundation guidelines, Kt/V= 1.2 per haemodialysis session for patients treated thrice weekly [2].

References:

1. Daugirdas JT: Second generation logarithmic estimates of single-pool variable volume Kt/V: an analysis of error. *J Am Soc Nephrol,* 4: 1205-1213, 1993
2. National Kidney Foundation NK: I. NKF-K/DOQI Clinical Practice Guidelines for Haemodialysis Adequacy: update 2000. *Am J Kidney Dis*, 37: S7-S64, 2001

2. Baseline characteristics of the patients

The aetiologies of the ESRD in the patients from both groups were balanced between the groups and representative of the most common causes of ESRD. In the HF group, there were patients suffering from diabetes mellitus (1 patient), glomerulonephritis (2 patient), polycystic kidney disease (1 patient), nephroangiosclerosis and renal ischemia (1 patient), interstitial nephritis (2 patient), multiple myeloma (1 patient), amyloidosis (1 patient) and glomerular hyperfiltration (1 patient). The renal diseases in the HDF group were diabetes mellitus (1 patient), glomerulonephritis (1 patient), polycystic kidney disease (1 patient), nephroangiosclerosis and renal ischemia (1 patient), interstitial nephritis (2 patient), amyloidosis (1 patient), bilateral nephrectomy (1 patient) and unknown (1 patient). In the HDF group there was a patient who had undergone a bilateral nephrectomy due to a renal carcinoma; and a patient whose nephropathy was undiagnosed.

All the patients had a glomerular filtration rate (GFR) < 6 ml/ min (end-stage kidney disease, chronic kidney disease stage 5, GFR< 15 ml/min). There were two patients with diabetes mellitus (requiring the administration of insulin) in each group. The Charlson Comorbidity Index (CCI) was used to assess the base comorbidity of these patients. The CCI considers 17 comorbidities and subcategories for liver disease and diabetes mellitus (Roffman 2016). Patients from both groups received subcutaneous recombinant human erythropoietin (rhEPO) weekly. Though, the requirements of erythropoietin varied significantly between both groups. Patients were also prescribed iron (1-2 vials of 1 g, monthly), folic acid (50 mg) and vitamin D (individualized dosage) supplementation.

3. Preparation of plasma samples

The patient's blood sample was drawn after the long interdialytic period in EDTA-K_3 tubes. The samples were then centrifuged at 900g for 10 minutes and the plasma was separated. Aliquots were stored at -80°C. To study plasma proteome, purification of pooled or individual plasma samples was performed removing high abundant proteins to improve the resolution of gels and detection of low molecular weight proteins using the Pure Proteome™ Albumin/IgG Depletion kit (Millipore), as recommended by the supplier. Plasma sample corresponding to

each patient was stored separately at -80°C, and protein concentrations were determined for each sample.

To check the depletion of these proteins, both the unprocessed and the albumin/IgG-depleted plasma samples were run into SDS-PAGE, and the gel was stained with Coomassie blue for protein detection. Supplemental Figure S1 shows the effective depletion of albumin/IgGs in HF and HDF pooled plasma samples compared to unprocessed HF and HDF pooled plasma samples.

4. 1-D DIGE analysis

4.1. Fluorescence labelling. DIGE assay

Plasma samples from HF and HDF patients were analyzed by one-dimensional fluorescence difference in gel electrophoresis (1-D DIGE). Albumin/IgG-depleted HF and HDF patients' plasma samples (5 μg of protein) were made to 8.5 M urea, centrifuged and labelled on cysteine residues with Cy3 or Cy5 fluorescent dyes (GE Healthcare). After dithiothreitol addition, HF and HDF samples were combined and fluorescence-labelled proteins loaded into standard vertical slab SDS-PAGE for 1-D DIGE. SDS-PAGE was performed in 12% acrylamide (3% cross-linking) gels (1.0 mm thick). The gel was scanned using a Typhoon 9200 imager (GE Healthcare) at 450 V per channel, and the paired Cy5/Cy3 images captured at 100 μm/pixel resolution for analysis. Cy5 was excited by the laser red-line (633 nm), and Cy3 was excited by the laser green-line (532 nm), sequentially and using appropriate emission filters (Cy5, 670 band-pass 30; Cy3, 580 band-pass 30) to minimize crosstalk. After scanning, the gel was stained with Coomassie blue.

4.2. In-gel protein digestion

Firstly, excised stained gel bands were destaining adding 200 μl of 50% ethanol (vol/vol) in 25 mM ammonium bicarbonate (AB) for 20 min, and after it was removed, added 200 μl of pure ethanol for 15 min, removed it and dried for 5 min. After, in-gel reduction and alkylation of proteins was carried out covering the bands with 200 μl 10 mM dithiothreitol (DTT) in 50 mM AB for 30 min at 56 °C, and after DTT was removed, washed with 50 mM AB for 5 min and bands incubated with 55 mM iodoacetamide in 50 mM AB for 30 min in the dark. Then, iodoacetamide was removed and washed twice with 50 and 25 mM AB for 5 min. Gel pieces were dehydrated adding 200 μl of 50% acetonitrile (ACN) (vol/vol) in 25 mM AB for 20 min and removed, it was repeated again, then 200 μl of neat ACN were added for 10 min, and

after ACN removal, gel pieces were dried completely. Then, the gel pieces were saturated with trypsin adding 15 µl of a solution containing 40 ng of trypsin (1:20 w/w) in 25 mM AB and leaving them in an ice bucket or a fridge for 45 min. After removing liquid excess, 20 µl of 25 mM AB was added and incubated overnight at 30°C. Finally, to extract the peptide digestion products, 1 µl of neat ACN were added (final concentration 6-15% ACN), incubated at 37°C for 15 min, and then 0.2 µl of trifluoroacetic (TFA) was added to 0.1% (vol/vol) final concentration and incubated for 10 min at room temperature, collecting the supernatant for mass spectrometry analysis.

4.3. Protein identification by mass spectrometry

After digestion, peptide mass fingerprinting (PMF) was analyzed by matrix-assisted laser desorption/ionization time-of-flight mass spectrometry (MALDI-TOF MS) (Autoflex III TOF/TOF, Bruker-Daltonics) for protein identification, as previously described [3]. PMF spectra were obtained using an alpha-cyanocinnamic acid matrix and calibrated with a peptide calibration standard mixture (222570, Bruker Daltonics) contained nine standard peptides in a molecular mass range between ~ 700 and 3500 Da to reach a typical mass measurement accuracy < ±3 ppm. Additionally, when available and for confirmation of protein identity, peptide fragmentation was performed by MS in tandem MALDI LIFT-TOF/TOF [4]. MS data of PMFs and MS/MS data from LIFT TOF/TOF spectra were searched in the SwissProt database using the MASCOT database search algorithm (Matrix Science) for protein identification. No more than one missed tryptic cleavage was allowed and a mass accuracy of 50 ppm was used for all mass searches.

References:

3. Cid C, Garcia-Bonilla L, Camafeita E, Burda J, Salinas M, Alcazar A: Proteomic characterization of protein phosphatase 1 complexes in ischemia-reperfusion and ischemic tolerance. *Proteomics*, 7: 3207-3218, 2007

4. Suckau D, Resemann A, Schuerenberg M, Hufnagel P, Franzen J, Holle A: A novel MALDI LIFT-TOF/TOF mass spectrometer for proteomics. *Anal Bioanal Chem*, 376: 952-965, 2003

5. LC-MS/MS Analysis

5.1 Protein digestion

Plasma samples were boiled for 5 min at 100 ºC in the presence of 25 mM Tris-HCl pH 6.8, 1% SDS and 50 mM DTT. This mixture (6 µl) was diluted with denaturing buffer (8 M urea

in 100 mM Tris-HCl pH 8.5) and concentrated on FASP filters (Expedeon). After washing with denaturing buffer at 10,000 rpm for 15 min, free thiol groups were alkylated by incubation with 50 mM iodoacetamide 30 min at room temperature in the dark. Then the filters were washed with denaturing buffer followed by washing with trypsin digestion buffer (50 mM ammonium bicarbonate pH 8.8). Protein samples were digested overnight at 37ºC with sequencing grade trypsin (Promega, Madison, WI, USA) at 1:40 (w/w) trypsin:protein ratio in digestion buffer. The resulting tryptic peptides from each sample were recovered by centrifugation at 10,000 rpm for 5 min after addition of 40 µl of trypsin digestion buffer, after which 50 µl of 500 mM NaCl were added and the filters centrifuged for 15 min at 10,000 rpm. Trifluoroacetic acid was added to a final concentration of 1% and the peptides were desalted on C18 Oasis HLB extraction cartridges (Waters Corporation, Milford, MA, USA) and dried-down.

5.2. Isobaric labelling of peptides using tandem tass tags (TMT)

The dried peptides were dissolved in triethylammonium bicarbonate buffer, and their concentration was determined using a Direct Detect IR spectrometer (Millipore). Equal amounts of each peptide sample were labeled with isobaric 10-plex Tandem Mass Tags (TMT, Thermo Scientific) according to the manufacturer's instructions and mixed together. An internal control was prepared by pooling the nine Control samples and was used as a reference to express relative quantification values. Samples were desalted using C18 Oasis HLB extraction cartridges (Waters) and dried-down for later LC-MS/MS analysis.

5.3. Peptide identification

Peptide samples were taken up in 0.1% formic acid and applied to an EASY-nLC 1000 nano-flow HPLC system (Thermo Fisher Scientific) coupled on-line with an orbitrap Fusion mass spectrometer (Thermo Fisher Scientific). C18-based reverse phase separation was used with a 2-cm trap column and a 50-cm analytical column (EASY-Spray, Thermo Fisher Scientific). Peptides were loaded in buffer A (0.1% formic acid (v/v)) and eluted with a 300-min linear gradient of buffer B (90% ACN, 0.1% formic acid (v/v)) at 200 nL/min flow. Mass spectra were acquired in a data-dependent manner, with an automatic switch between MS and MS/MS, using a top-speed method and 30 s dynamic exclusion. MS spectra were acquired in the 400–1500 m/z range at 120,000 resolution, while HCD MS/MS were performed at 33 normalized collision energy and analyzed with 35,000 resolution in the orbitrap.

LC-MS/MS data were analyzed with Proteome Discoverer (version 2.1, Thermo Fisher Scientific) using SEQUEST-HT (Thermo Fisher Scientific) against a Uniprot database containing all sequences from Homo sapiens (February 2020; 148,000 entries). Database search parameters were selected as follows: trypsin digestion with two maximum missed cleavage sites, precursor mass tolerance of 800 ppm, fragment mass tolerance of 20 mmu. Met oxidation was considered a variable modification, while Cys carbamidomethylation and Lys and peptide N-terminal modification of +229.163 were set as fixed modifications for TMT. The corresponding inverted protein sequences were incorporated to the database for false discovery rate (FDR) calculation. Peptide identification from MS/MS data was performed using the probability ratio method [5], and the FDR of peptide identifications was calculated using the refined method [6,7], taking 1% FDR as a threshold for peptide identification. Peptides were assigned only to the best protein proposed by the Proteome Discoverer algorithm.

References:

5. Martinez-Bartolome, S. *et al.* Properties of average score distributions of SEQUEST: the probability ratio method. *Mol Cell Proteomics* **7**, 1135-1145 (2008).
6. Navarro, P. & Vazquez, J. A refined method to calculate false discovery rates for peptide identification using decoy databases. *J Proteome Res* **8**, 1792-1796 (2009).
7. Bonzon-Kulichenko, E., Garcia-Marques, F., Trevisan-Herraz, M. & Vazquez, J. Revisiting peptide identification by high-accuracy mass spectrometry: problems associated with the use of narrow mass precursor windows. *J Proteome Res* **14**, 700-710 (2015).

5.4. Quantification at the peptide and protein levels

The quantitative information extracted from the MS/MS spectra by Proteome Discoverer was integrated from the spectrum level to the peptide level and then to the protein level on the basis of the WSPP model [8] and the systems biology triangle algorithm [9] using the SanXoT software package [10]. Briefly, the log2-ratio of every scan was calculated using the TMT reporter ion intensities coming from samples A and B. The log2-ratio of every peptide was then calculated as the weighted average of its scans, whereas the quantification of each protein was the weighted average of its peptides, and averaged of all the protein values [8].

Peptide quantification weights were calculated from the corresponding scan weights and the peptide variance, and protein quantification weights were calculated from the corresponding peptide weights and the protein variance. Thus, protein quantification was defined by a Zq

value as a normalized log2-ratio at the protein level expressed in standard deviation units [8]. Outliers at the peptide and protein levels were detected at 1% FDR, and a FDRq value < 0.05 was defined as significant [8] .

References:

8. Navarro, P. *et al.* General statistical framework for quantitative proteomics by stable isotope labeling. *J Proteome Res* **13**, 1234-1247 (2014).

9. Garcia-Marques, F. *et al.* A Novel Systems-Biology Algorithm for the Analysis of Coordinated Protein Responses Using Quantitative Proteomics. *Mol Cell Proteomics* **15**, 1740-1760 (2016).

10. Trevisan-Herraz, M. *et al.* SanXoT: a modular and versatile package for the quantitative analysis of high-throughput proteomics experiments. *Bioinformatics* **35**, 1594-1596 (2019).

6. Quantification of TTR peptides by mass spectrometry. Calibration experiments

Calibration experiments were previously carried out to test the accuracy of the quantification using known concentrations of reference peptides bradykinin (765.85 Da), bombesin (1619.85 Da) and adrenocorticotropic hormone (ACTH, 2465.67 Da), in addition to angiotensin-II peptide (1045.54 Da). Peptide relative intensities were obtained from MS spectra in the same conditions as for peptide mass fingerprinting analysis by MALDI-TOF MS (see above). Reference peptides were quantified with respect to the angiotensin-II peptide as a ratio of the relative intensities of peptide/angiotensin-II. The reference peptide concentrations used were 2-100 fmol, with 4 fmol of angiotensin-II, and 4-400 fmol, with 40 fmol of angiotensin-II.

To quantify transthyretin (TTR) peptides, the angiotensin-II peptide was added as an internal standard to obtain a relative intensity of the TTR peptides. The accuracy of MS peptide quantification was confirmed with calibration experiments carried out with increasing concentrations of reference peptides (molecular mass range from ~ 765 to 2465 Da) (Figure S4). The calibration experiments showed a strict correlation between peptide concentration and relative intensity for each peptide (Figure S4). TTR peptides with 1366.75, 1394.62, 1416.77, 1522.71, 2451.20 and 3140.51 Da were identified by MASCOT database search algorithm (Matrix Science, http://www.matrixscience.com), in addition to 833.40 Da TTR peptide, identified by FindPept tool (ExPASy, https://web.expasy.org/findpept/) (Figure 4A). Accordingly, TTR peptides from MALDI-TOF MS spectra were quantified in the mass rage of the calibration experiments (TTR peptides from 833.40 to 2451.20 Da) as relative intensities (ratio TTR peptide intensity/angiotensin-II intensity).

7. Detection of the allele distribution of haptoglobin α in HF and HDF patients

Haptoglobin is a glycoprotein with α and β chains. In human, haptoglobin exists in two allelic forms designated Haptoglobin 1 (*Hp1*) and Haptoglobin 2 (*Hp2*) that results in three known phenotypes, Hp1-1, Hp2-2 and the heterozygous phenotype Hp2-1. *Hp*2 contain the allele *Hp*α2 that is the product of a partial *Hp*α1 gene duplication [11]. Thus, the product of *Hp*α2 gives a protein with ~ 7 kDa greater than that of *Hp*α1.

In our experiments, haptoglobin α2 was detected as proteins 20 and 21 (Figure 3A), showing similar intensity values in both HF and HDF patient samples (Figure 3B). In these experiments, haptoglobin α1 was significantly detected with higher levels in HF patients than in HDF patients (Figure 1B, protein *f*; and Figure 3B). However, the allele distribution between HF and HDF groups should be tested. To these end, we analysed the expression of haptoglobin α2 and α1 in HF and HDF samples by western blot using a specific anti-haptoglobin α antibody. The results showed two proteins identified by the antibody, which according to the molecular mass standards used, would correspond to haptoglobin α2 and α1 (Figure S3). These results also showed that three HF patients (patients 3, 6, and 7) had not haptoglobin α2 expression –patient HF 9 showed a very slight expression of haptoglobin α2– (Figure S3), having therefore the phenotype Hp1-1. On the other hand, only one HDF patient (patient 6) had not haptoglobin α2, being of the Hp1-1 phenotype. As expected, the phenotype Hp1-1 induces more expression of haptoglobin α1 than the phenotypes Hp2-1 and Hp2-2 (Figure S3, see patients HF 3, 6 and 7 and HDF 6), and therefore, the presence of more subjects with phenotype Hp1-1 among HF patients can be the cause of the higher levels of haptoglobin α1 found in this group.

References:

11. Yang, F., Brune, J. L., Baldwin, W. D., Barnett, D. R. & Bowman, B. H. Identification and characterization of human haptoglobin cDNA. *Proc Natl Acad Sci U S A* **80**, 5875-5879 (1983).

8. Supplementary Table S1

Table S1. Proteins identified by MALDI-TOF MS in plasma samples of HF and HDF patients.

N°	Protein	Accession no. [a]	Gene name	Theoretical mass (Da)	Score [b]	Peptides matched/ searched	% Coverage	Lift [c] (score)
1	Alpha-2-macroglobulin	P01023	*A2M*	164613	95	24/86	20	1148.62(63)
2	Ceruloplasmin	P00450	*CP*	122983	112	19/66	24	2155.05(80)
3	Inter-alpha-trypsin inhibitor heavy chain H4	Q14624	*ITIH4*	103521	48	6/26	10	2184.07(50)
7	Serotransferrin	P02787	*TF*	79294	152	25/95	33	1881.91(91)
**12**	**Alpha-1-antitrypsin**	**P01009**	***SERPINA1***	**46737**	**98**	**10/41**	**27**	**1641.83(99)**
13	Apolipoprotein A-IV	P06727	*APOA4*	45371	127	17/72	37	1352.69(76)
14	Haptoglobin β	P00738	*HP*	27265	109	14/85	33	1707.87(75)
15	Haptoglobin β	P00738	*HP*	27265	123	13/29	33	
18	Apolipoprotein A-I	P02647	*APOA1*	30759	191	23/98	71	
19	Retinol-binding protein 4	P02753	*RBP4*	23337	70	5/13	33	2693.10(65)
20	Haptoglobin α2	P00738	*HP*	15945	62	8/52	21	
21	Haptoglobin α2	P00738	*HP*	15945	60	6/38	16	3432.62(70)
**22**	**Transthyretin**	**P02766**	***TTR***	**13761**	**64**	**6/65**	**61**	**2451.20(189)**
**23**	**Haptoglobin α1**	**P00738**	***HP***	**9192**	**64**	**10/81**	**19**	**1708.91(91)**
24	Serum amyloid A1	P0DJI8	*SAA1*	13581	63	5/39	55	

[a], Accession number in UniProt database (https://www.uniprot.org). [b],Protein identification scores > 56 were significant ($p < 0.05$) in the MASCOT database search algorithm. [c], MALDI LIFT-TOF/TOF MS identification mode; the m/z of the fragmented parental peptide is indicated; MASCOT scores (in parenthesis) > 28 were significant ($p < 0.05$). Proteins marked in bold were significant by DIGE quantification (Figure 1; $p < 0.05$, t-test) with higher levels in plasma samples of HF compared with HDF patients.

9. Supplementary Table S2

Table S2. Proteins quantified in LC-MS/MS analysis in plasma samples of HDF compared with HF patients.

Protein	Accession no. [a]	Gene name	Theoretical mass (Da)	Peptides identified	Zq value [b]	Zq value (colour code) [c]
cDNA FLJ53691, highly similar to Serotransferrin	B4E1B2		74832	4	3.04 *,#	
Trypsin-1	P07477	*PRSS1*	26558	2	2.42 *	
Immunoglobulin heavy constant alpha 1	P01876	*IGHA1*	37655	14	2.09 *	
Dystroglycan	Q14118	*DAG1*	97441	2	1.77	
Immunoglobulin heavy variable 4-28	A0A0C4DH34	*IGHV4-28*	13124	3	1.57	
Immunoglobulin delta heavy chain	P0DOX3		56224	2	1.50	
Immunoglobulin heavy constant gamma 3	P01860	*IGHG3*	41287	6	1.43	
Immunoglobulin heavy variable 3-7	P01780	*IGHV3-7*	12943	2	1.42	
Rheumatoid factor G9 light chain (Fragment)	A0N5G3	*V-lambda-3*	12897	2	1.32	
IBM-A3 heavy chain variable region (Fragment)	A0A0X9UWM4		14369	2	1.28	
Uncharacterized protein DKFZp686K04218 (Fragment)	Q7Z379	*DKFZp686K 04218*	51620	2	1.23	
Vitronectin	D9ZGG2	*VTN*	54306	5	1.18	
Immunoglobulin heavy constant gamma 4	P01861	*IGHG4*	35941	7	1.11	
Ig heavy chain variable region (Fragment)	A0A068LKQ8		12449	2	1.05	
IBM-B3 light chain variable region (Fragment)	A0A109PW74		11399	2	1.03	
Immunoglobulin kappa constant	P01834	*IGKC*	11765	8	1.01	
V1-22 protein (Fragment)	Q5NV88	*V1-22*	10643	2	1.00	
Alpha-2-macroglobulin	P01023	*A2M*	163291	70	0.94	
Immunoglobulin heavy constant gamma 1	P01857	*IGHG1*	36106	17	0.93	
Properdin	P27918	*CFP*	51276	3	0.87	
B cell receptor heavy chain variable region (Fragment)	A0A1C9J6R2		14524	2	0.87	
Immunoglobulin lambda-like polypeptide 5	B9A064	*IGLL5*	23063	4	0.87	
Immunoglobulin lambda constant 2	P0DOY2	*IGLC2*	11294	2	0.85	
Cartilage oligomeric matrix protein	P49747	*COMP*	82860	4	0.79	
Immunoglobulin lambda constant 7	A0M8Q6	*IGLC7*	11254	4	0.72	
Immunoglobulin heavy variable 3-49	A0A0A0MS15	*IGHV3-49*	13056	3	0.69	
Immunoglobulin heavy constant alpha 2	P01877	*IGHA2*	36591	2	0.67	
Serum amyloid A protein	D3DQX7	*SAA1*	13562	2	0.65	
Carboxypeptidase N subunit 2	P22792	*CPN2*	60557	9	0.62	
Immunoglobulin lambda-like polypeptide 1	P15814	*IGLL1*	22963	4	0.61	
MS-F1 light chain variable region (Fragment)	A0A0X9V9B3		12108	2	0.60	
Immunoglobulin kappa variable 3D-20	A0A0C4DH25	*IGKV3D-20*	12515	2	0.59	
Beta-Ala-His dipeptidase	Q96KN2	*CNDP1*	56706	7	0.56	
Immunoglobulin alpha-2 heavy chain	P0DOX2		48934	4	0.54	
MS-F1 heavy chain variable region (Fragment)	A0A125U0V1		13509	2	0.51	
Immunoglobulin kappa variable 4-1	P06312	*IGKV4-1*	13380	2	0.51	
Ig heavy chain variable region (Fragment)	A0A2U8J8Y4	*IgH*	12010	2	0.46	
Pregnancy zone protein	P20742	*PZP*	163863	16	0.44	
IBM-A1 light chain variable region (Fragment)	A0A0X9USN3		11626	2	0.44	
Myosin-reactive immunoglobulin light chain variable region (Fragment)	Q9UL83		11834	2	0.42	
Immunoglobulin kappa variable 3D-7	A0A0C4DH55	*IGKV3D-7*	13148	2	0.42	
IBM-B2 heavy chain variable region (Fragment)	A0A125QYY9		13569	2	0.39	
Fibulin-1	P23142	*FBLN1*	77214	9	0.38	
Afamin	P43652	*AFM*	69069	17	0.38	
Carboxypeptidase N catalytic chain	P15169	*CPN1*	52286	4	0.37	
Biotinidase	P43251	*BTD*	61133	4	0.36	
IgGFc-binding protein	Q9Y6R7	*FCGBP*	572017	2	0.33	
Vitamin K-dependent protein S	P07225	*PROS1*	75123	18	0.27	
Thyroxine-binding globulin	P05543	*SERPINA7*	46325	14	0.26	
Immunoglobulin kappa variable 3-7 (non-functional) (Fragment)	A0A075B6H7	*IGKV3-7*	12783	2	0.24	

Ig heavy chain variable region (Fragment)	A0A2U8J8Z6	*IgH*	10905	2	0.22	
Fibronectin	P02751	*FN1*	272320	54	0.22	
Apolipoprotein(a)	P08519	*LPA*	501319	10	0.21	
Complement factor B	P00751	*CFB*	85533	18	0.21	
Immunoglobulin kappa variable 1-6	A0A0C4DH72	*IGKV1-6*	12697	2	0.19	
Coagulation factor IX	P00740	*F9*	51778	5	0.18	
CP protein	A5PL27	*CP*	122205	33	0.15	
Carbonic anhydrase 1 (Fragment)	E5RFE7	*CA1*	21402	3	0.13	
Apolipoprotein C-I	P02654	*APOC1*	9332	5	0.12	
Hyaluronan-binding protein 2	Q14520	*HABP2*	62672	10	0.12	
Immunoglobulin kappa variable 3D-15	A0A087WSY6	*IGKV3D-15*	12534	3	0.11	
von Willebrand factor	P04275	*VWF*	309265	16	0.11	
Gelsolin	A0A0A0MS51	*GSN*	82526	14	0.11	
Immunoglobulin J chain	P01591	*JCHAIN*	18099	4	0.09	
Hepatocyte growth factor activator	Q04756	*HGFAC*	70682	7	0.09	
Complement C3	P01024	*C3*	187148	100	0.09	
SPARC-like protein 1	Q14515	*SPARCL1*	75208	2	0.09	
Complement component C8 gamma chain	P07360	*C8G*	22277	7	0.07	
IGK@ protein	Q6PIL8	*IGK@*	25834	2	0.05	
Selenoprotein P	P49908	*SELENOP*	43174	2	0.05	
Prothrombin	P00734	*F2*	70037	31	0.05	
Apolipoprotein F	Q13790	*APOF*	35399	3	0.05	
cDNA FLJ51266, highly similar to Vitronectin	B7Z553		23605	3	0.05	
Angiotensinogen (Fragment)	A7L3A3	*AGT*	9407	4	0.04	
N-acetylmuramoyl-L-alanine amidase	Q96PD5	*PGLYRP2*	62217	8	0.02	
Immunoglobulin heavy variable 3-9	P01782	*IGHV3-9*	12945	3	0.01	
Immunoglobulin heavy constant delta	P01880	*IGHD*	42353	4	0.00	
Immunoglobulin heavy variable 3-15	A0A0B4J1V0	*IGHV3-15*	12926	2	-0.01	
Cystatin-C	P01034	*CST3*	15799	6	-0.02	
Immunoglobulin lambda variable 3-10	A0A075B6K4	*IGLV3-10*	12441	2	-0.02	
Myosin-reactive immunoglobulin kappa chain variable region (Fragment)	Q9UL86		11928	3	-0.05	
MS-C1 heavy chain variable region (Fragment)	A0A125U0U7		13097	2	-0.05	
Immunoglobulin heavy constant gamma 2	P01859	*IGHG2*	35901	13	-0.06	
Transforming growth factor-beta-induced protein ig-h3	Q15582	*TGFBI*	74681	3	-0.06	
Angiotensinogen (Serpin peptidase inhibitor, clade A, member 8)	B0ZBE2	*AGT*	53154	5	-0.07	
EGF containing fibulin-like extracellular matrix protein 1 isoform 2	A0A0S2Z3V1	*EFEMP1*	45723	5	-0.07	
Coagulation factor X	P00742	*F10*	54732	5	-0.07	
C4b-binding protein alpha chain	P04003	*C4BPA*	67033	26	-0.08	
Peroxiredoxin-2	P32119	*PRDX2*	21892	4	-0.09	
Ig heavy chain variable region (Fragment)	A0A2U8J9B3	*IgH*	12311	2	-0.10	
Ig heavy chain variable region (Fragment)	A0A2U8J8R6	*IgH*	10922	2	-0.14	
Serum albumin	P02768	*ALB*	69367	76	-0.16	
Plasma kallikrein	P03952	*KLKB1*	71370	12	-0.16	
Pigment epithelium-derived factor	P36955	*SERPINF1*	46312	11	-0.17	
Sulfhydryl oxidase 1	O00391	*QSOX1*	82578	2	-0.18	
Apolipoprotein C-IV	P55056	*APOC4*	14553	2	-0.18	
Ig heavy chain variable region (Fragment)	A0A2U8J8Q6	*IgH*	10379	2	-0.19	
Fibrinogen alpha chain	P02671	*FGA*	94973	41	-0.20	
Platelet glycoprotein Ib alpha chain	P07359	*GP1BA*	71540	2	-0.20	
Coagulation factor V	P12259	*F5*	251703	12	-0.21	
Chromogranin-A	P10645	*CHGA*	50688	9	-0.22	
Adiponectin A	A0A024RAB9	*C1QB*	26722	5	-0.22	
Immunoglobulin heavy variable 3-21	A0A0B4J1V1	*IGHV3-21*	12840	3	-0.23	
Apolipoprotein D	P05090	*APOD*	21276	7	-0.23	
Fetuin-B	Q9UGM5	*FETUB*	42055	5	-0.24	
Immunoglobulin heavy variable 4-4	A0A075B6R2	*IGHV4-4*	12848	4	-0.25	
Inter-alpha-trypsin inhibitor heavy chain H4	Q14624	*ITIH4*	103357	26	-0.26	
Complement factor H	P08603	*CFH*	139096	54	-0.26	
Heavy chain of factor I (Fragment)	Q6LAM1		35883	2	-0.26	
Immunoglobulin heavy variable 3-72	A0A0B4J1Y9	*IGHV3-72*	13203	3	-0.28	
Immunoglobulin kappa variable 3D-11	A0A0A0MRZ8	*IGKV3D-11*	12625	2	-0.28	
C-reactive protein	P02741	*CRP*	25039	6	-0.29	
Monocyte differentiation antigen CD14	P08571	*CD14*	40076	4	-0.29	
Actin, cytoplasmic 1	P60709	*ACTB*	41737	5	-0.30	

Apolipoprotein B-100	P04114	*APOB*	515605	178	-0.31	
Complement factor D	P00746	*CFD*	27033	9	-0.31	
Complement component C8 alpha chain	P07357	*C8A*	65163	15	-0.31	
Complement component C6	P13671	*C6*	104786	17	-0.31	
Lipopolysaccharide-binding protein	P18428	*LBP*	53384	5	-0.33	
Glutathione peroxidase 3	P22352	*GPX3*	25552	5	-0.33	
Fibrinogen beta chain	P02675	*FGB*	55928	43	-0.34	
Complement factor I	P05156	*CFI*	65750	17	-0.34	
Inter-alpha-trypsin inhibitor heavy chain H1	P19827	*ITIH1*	101389	18	-0.34	
Apolipoprotein A-I	P02647	*APOA1*	30778	38	-0.35	
Inter-alpha-trypsin inhibitor heavy chain H2	P19823	*ITIH2*	106463	21	-0.37	
Alpha-1B-glycoprotein	P04217	*A1BG*	54254	10	-0.37	
Immunoglobulin kappa light chain	P0DOX7		23379	4	-0.38	
Complement component C8 beta chain	P07358	*C8B*	67047	13	-0.38	
Serotransferrin	P02787	*TF*	77064	56	-0.38	
Complement C5	P01031	*C5*	188305	38	-0.39	
Fibrinogen gamma chain	P02679	*FGG*	51512	31	-0.39	
GCT-A1 light chain variable region (Fragment)	A0A0X9TDD0	*2 SV*	12436	2	-0.39	
Alpha-2-HS-glycoprotein	P02765	*AHSG*	39341	10	-0.41	
Cystatin-M	Q15828	*CST6*	16511	2	-0.42	
Platelet basic protein	P02775	*PPBP*	13894	2	-0.43	
Fibronectin 1, isoform CRA_n	A0A024R462	*FN1*	259211	2	-0.43	
Immunoglobulin heavy variable 3-74	A0A0B4J1X5	*IGHV3-74*	12840	4	-0.43	
Plasma serine protease inhibitor	P05154	*SERPINA5*	45675	5	-0.44	
Vasorin	Q6EMK4	*VASN*	71713	2	-0.46	
Mannan-binding lectin serine protease 1	P48740	*MASP1*	79247	5	-0.47	
Carbonic anhydrase 1 (Fragment)	E5RFL2	*CA1*	13256	2	-0.48	
Lectin galactoside-binding soluble 3 binding protein isoform 1	A0A0S2Z3Y1	*LGALS3BP*	65331	7	-0.49	
Heparin cofactor 2	P05546	*SERPIND1*	57071	15	-0.49	
Kininogen-1	P01042	*KNG1*	71957	28	-0.52	
Prenylcysteine oxidase 1	Q9UHG3	*PCYOX1*	56640	3	-0.54	
Basement membrane-specific heparan sulfate proteoglycan core protein	P98160	*HSPG2*	468830	2	-0.57	
Mutant hemoglobin alpha 2 globin chain	A0A0K2BMD8	*HBA2*	15258	6	-0.60	
Adiponectin B	A0A024RAA7	*C1QC*	25774	2	-0.60	
IBM-B1 light chain variable region (Fragment)	A0A125QYY8		12167	2	-0.63	
Complement factor H-related protein 1	Q03591	*CFHR1*	37651	3	-0.63	
Inter-alpha-trypsin inhibitor heavy chain H3	Q06033	*ITIH3*	99849	15	-0.63	
Clusterin	P10909	*CLU*	52495	18	-0.65	
Complement C1s subcomponent	P09871	*C1S*	76684	16	-0.66	
Coagulation factor XIII B chain	P05160	*F13B*	75511	15	-0.67	
Vitamin K-dependent protein C	P04070	*PROC*	52071	3	-0.69	
N90-VRC38.01 light chain variable region (Fragment)	A0A1W6IYJ9		12352	4	-0.69	
Plasminogen	P00747	*PLG*	90569	32	-0.69	
Serum amyloid P-component	P02743	*APCS*	25387	7	-0.69	
C4b-binding protein beta chain	P20851	*C4BPB*	28357	6	-0.71	
Alpha-1-antitrypsin	P01009	*SERPINA1*	46737	38	-0.71	
Testicular tissue protein Li 61	A0A140VJI7		60674	7	-0.72	
Epididymis secretory sperm binding protein	A0A384N669		38429	10	-0.72	
Carboxypeptidase B2	Q96IY4	*CPB2*	48424	7	-0.72	
Coagulation factor XIII A chain	P00488	*F13A1*	83267	4	-0.74	
Kallistatin	P29622	*SERPINA4*	48542	12	-0.74	
Insulin-like growth factor II	P01344	*IGF2*	20140	2	-0.76	
Alpha-2-antiplasmin	P08697	*SERPINF2*	54566	12	-0.77	
Corticosteroid-binding globulin	P08185	*SERPINA6*	45141	7	-0.78	
Immunoglobulin kappa variable 1-27	A0A075B6S5	*IGKV1-27*	12712	2	-0.79	
Phosphatidylinositol-glycan-specific phospholipase D	P80108	*GPLD1*	92336	7	-0.82	
Vitamin D-binding protein	P02774	*GC*	52918	29	-0.84	
Coagulation factor XII	P00748	*F12*	67792	8	-0.84	
Complement C2	P06681	*C2*	83268	16	-0.85	
Hemoglobin subunit delta	P02042	*HBD*	16055	9	-0.86	
Apolipoprotein L1	O14791	*APOL1*	43974	7	-0.86	
Phospholipid transfer protein	P55058	*PLTP*	54739	3	-0.86	
Hemoglobin subunit beta	P68871	*HBB*	15998	8	-0.86	
Vitamin D binding protein (Fragment)	A0A1B1CYC5	*Gc*	3718	2	-0.86	
Insulin-like growth factor binding protein 4, isoform	A0A024R1U8	*IGFBP4*	27934	2	-0.86	

CRA_a					
Serpin peptidase inhibitor clade G member 1 isoform 4	A0A0S2Z333	*SERPING1*	19647	10	-0.87
Serum paraoxonase/arylesterase 1	P27169	*PON1*	39731	7	-0.88
HCG40889, isoform CRA_b	A0A024R962	*hCG_40889*	139070	2	-0.88
Mannan-binding lectin serine protease 2	O00187	*MASP2*	75702	2	-0.88
Protein AMBP	P02760	*AMBP*	38999	18	-0.90
Immunoglobulin lambda variable 2-8	P01709	*IGLV2-8*	12382	2	-0.95
Histidine-rich glycoprotein	P04196	*HRG*	59578	12	-0.96
Retinol-binding protein 4	P02753	*RBP4*	23010	8	-0.96
Apolipoprotein C-II isoform 1	A0A024R0T9	*APOC4-APOC2*	11284	4	-0.97
Complement C1r subcomponent	P00736	*C1R*	80119	12	-0.98
Serum albumin	Q56G89		69084	2	-0.99
Apolipoprotein M	O95445	*APOM*	21253	4	-1.01
Complement component C7	P10643	*C7*	93518	17	-1.04
Serpin peptidase inhibitor, clade A (Alpha-1 antiproteinase, antitrypsin) member 3, isoform CRA_c	A0A024R6P0	*SERPINA3*	47651	22	-1.12
Hepatocyte growth factor-like protein	P26927	*MST1*	80320	5	-1.12
Attractin	O75882	*ATRN*	158537	10	-1.13
Serpin peptidase inhibitor clade G member 1	A0A348GSH7	*SERPING1*	49757	8	-1.15
Serum amyloid A-1 protein	P0DJI8	*SAA1*	13532	7	-1.17
Apolipoprotein A-II	P02652	*APOA2*	11175	7	-1.17
Complement C1r subcomponent-like protein	Q9NZP8	*C1RL*	53498	2	-1.20
Protein Z-dependent protease inhibitor	Q9UK55	*SERPINA10*	50707	2	-1.21
Alpha-1-acid glycoprotein 2	P19652	*ORM2*	23603	9	-1.22
Antithrombin-III	P01008	*SERPINC1*	52602	29	-1.22
Prostaglandin-H2 D-isomerase	P41222	*PTGDS*	21029	5	-1.24
Beta-2-microglobulin	P61769	*B2M*	13715	4	-1.27
Complement C4-A	P0C0L4	*C4A*	192785	65	-1.30
Complement factor H-related protein 2	P36980	*CFHR2*	30651	4	-1.36
Protein S100-A9	P06702	*S100A9*	13242	6	-1.38
Complement C4-B	P0C0L5	*C4B*	192751	4	-1.42
Insulin-like growth factor-binding protein 3	P17936	*IGFBP3*	31674	4	-1.44
Complement component C9	P02748	*C9*	63173	16	-1.46
Hemopexin	P02790	*HPX*	51676	24	-1.47
Immunoglobulin kappa variable 3-20	P01619	*IGKV3-20*	12557	2	-1.51
CD44 antigen	P16070	*CD44*	81538	2	-1.56
Immunoglobulin heavy variable 5-51	A0A0C4DH38	*IGHV5-51*	12675	4	-1.58
Alpha-1-acid glycoprotein	V9HWF6	*HEL-S-153w*	23512	6	-1.61
Serum amyloid A-4 protein	P35542	*SAA4*	14747	4	-1.62
Insulin-like growth factor-binding protein complex acid labile subunit	P35858	*IGFALS*	66035	10	-1.63
Insulin-like growth factor-binding protein 2	P18065	*IGFBP2*	34814	4	-1.63
CD5 antigen-like	O43866	*CD5L*	38088	11	-1.65
Haptoglobin	P00738	*HP*	45205	30	-1.71
Ficolin-3	O75636	*FCN3*	32903	5	-1.74
Apolipoprotein A-IV	P06727	*APOA4*	45372	33	-1.81
Apolipoprotein H (Beta-2-glycoprotein I), isoform CRA_a	A0A384NKM6	*APOH*	38298	12	-1.82
Leucine-rich alpha-2-glycoprotein	P02750	*LRG1*	38178	5	-1.86
Immunoglobulin heavy constant mu	P01871	*IGHM*	49440	15	-1.91
Transthyretin	P02766	*TTR*	15887	7	-1.97
Uncharacterized protein DKFZp686G11190	Q6MZQ6	*DKFZp686G 11190*	52043	3	-2.06 *
Apolipoprotein E	P02649	*APOE*	36154	17	-2.15 *
Full-length cDNA clone CS0DD006YL02	Q86TT1		41273	2	-2.30 *
Testicular tissue protein Li 227	A0A140VK00		34259	17	-2.30 *
Apolipoprotein C-III	A3KPE2	*APOC3*	10852	5	-2.48 *
Haptoglobin-related protein	P00739	*HPR*	39030	7	-3.19 *,#
Epididymis secretory sperm binding protein	A0A384MDQ7		46723	2	-4.45 *,#

[a], Accession number in UniProt database (https://www.uniprot.org). [b], Protein quantification values (Zq) are normalized log2-ratios expressed in standard deviation units: Zq > 0 and Zq < 0 indicate increased or decreased, respectively, protein abundance in HDF compared with HF patients. [c], Zq values in a colour scale; red and blue represent the increased or decreased protein levels, respectively, in HDF compared with HF. * Zq ≥ 2, and ≤ -2, were significant (p ≤ 0.05); [#], significant FDRq value (< 0.05).

10. Supplementary Figure S1

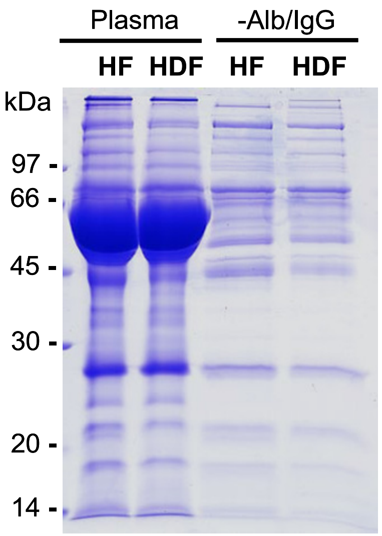

Figure S1. Plasma samples from HF and HDF patients were depleted from albumin and IgG. Plasma samples from patients on high-flux haemodialysis (HF) or on-line haemodiafiltration (HDF) were pooled, albumin and IgG depleted and ran into sodium dodecyl sulphate-polyacrylamide gel electrophoresis (SDS-PAGE), and the gel was stained with Coomassie blue for protein detection. The numbers on the left indicate the apparent molecular mass in kDa. The figure shows plasma samples from HF and HDF patients before and after albumin and IgG depletion (-Alb/IgG) in a whole stained gel.

11. Supplementary Figure S2

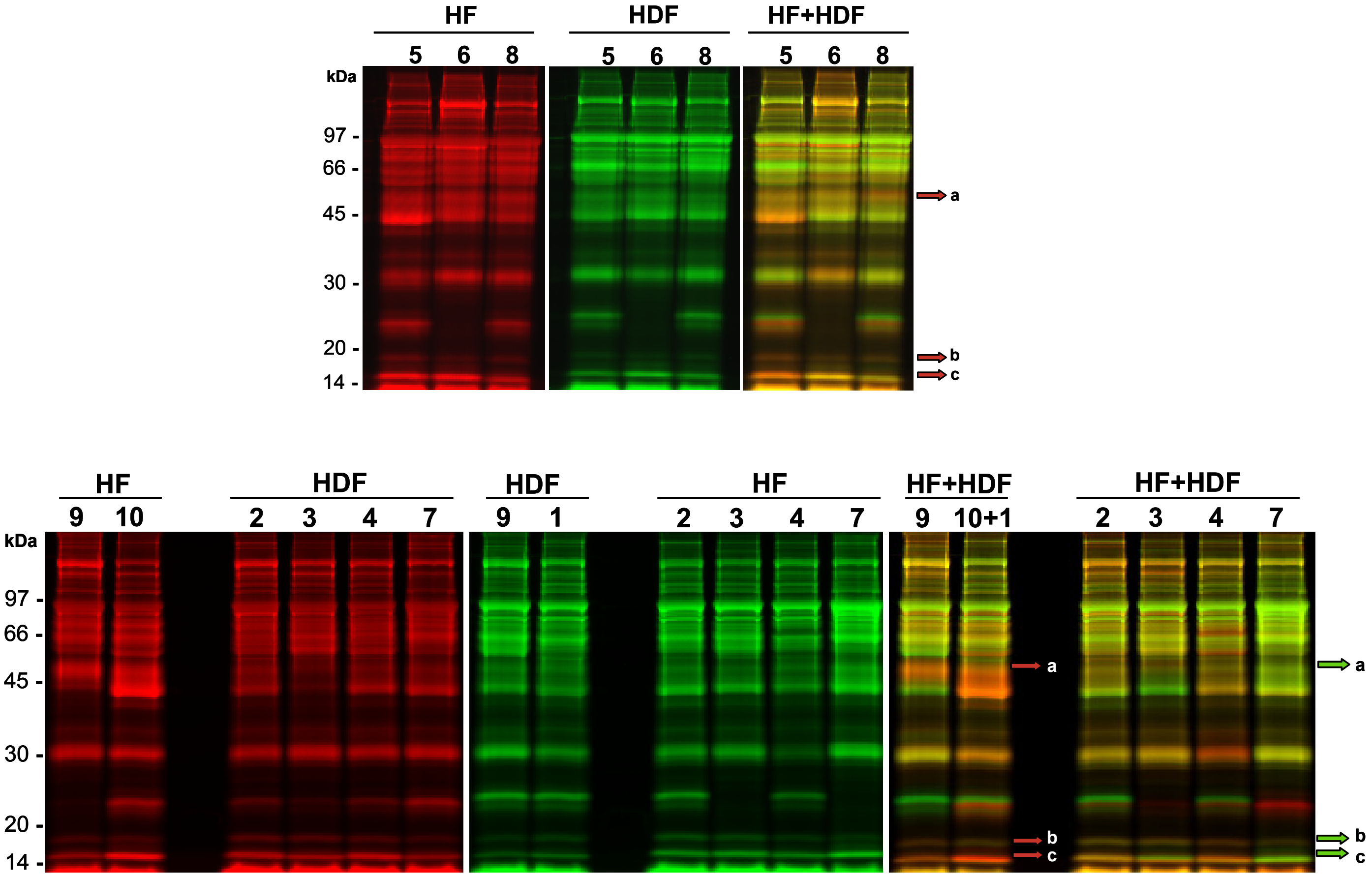

Figure S2. Differential protein detection of individual plasma samples of HF combined with HDF patients by fluorescence in gel electrophoresis (DIGE). The images show the combinations of individual paired and Cy-labelled samples of nine HF and HDF patients performed in the same way as described in Figure 1A. The scanned gel displays fluorescence-labelled proteins; proteins in the HF or HDF samples were visualised in red (Cy5-labelled) or green (Cy3-labelled). Proteins present in both HF and HDF samples were visualised in yellow due to the merge of the red and green labels (HF+HDF). The numbers on the left indicate the apparent molecular mass. The differentially detected proteins quantified in Figure 1B, *a* to *c*, are indicated in by arrows in the highest labelling colour code (red or green, HF patients). The fluorescence image corresponds to whole gels and adjacent empty lanes are not shown.

12. Supplementary Figure S3

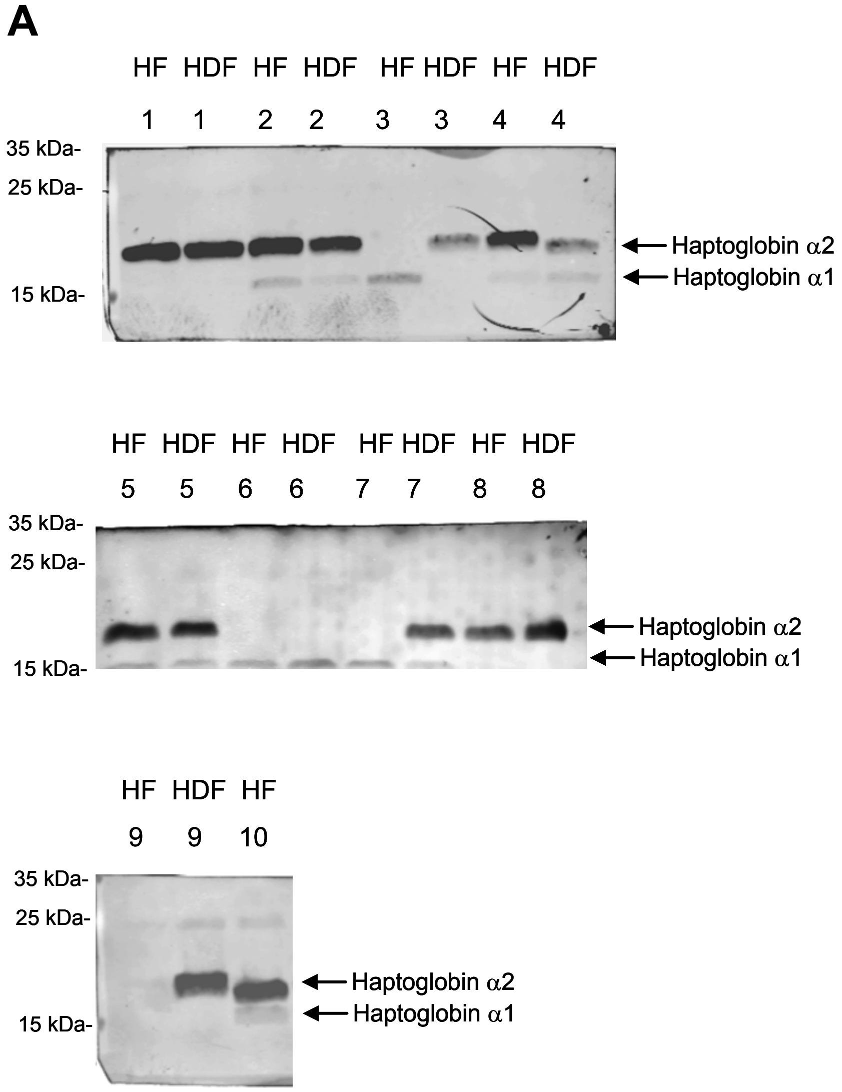

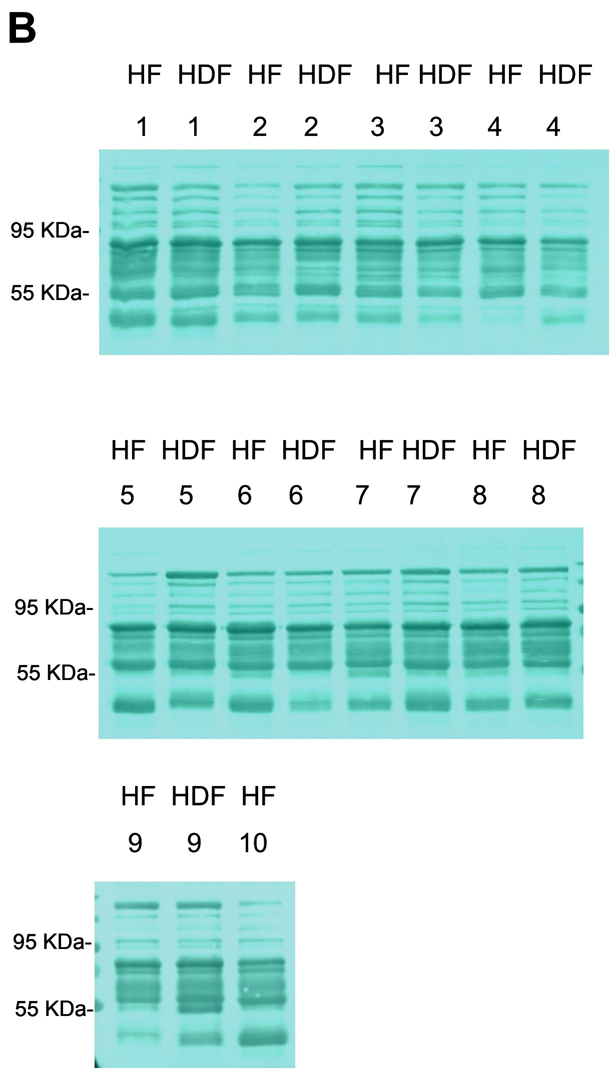

Figure S3. Haptoglobin α detection by western blot in HF and HDF plasma samples. (**A**) Individual albumin- and IgG-depleted plasma samples (5 µg of protein) from 10 and 9 HF and HDF patients, respectively, were analysed independently by western blotting as describe in Methods using a mouse monoclonal anti-haptoglobin α antibody (sc-376893, Santa Cruz Biotechnology). In the images, arrows show the haptoglobin α2 and α1 detected. The numbers on the left indicate the apparent molecular mass from standards. (**B**) Blotted proteins were staining with Fast Green as loading control of the analyzed samples. . The upper piece of the PVDF membranes containing most proteins was used for staining. Images show whole blots.

11. Supplementary Figure S4

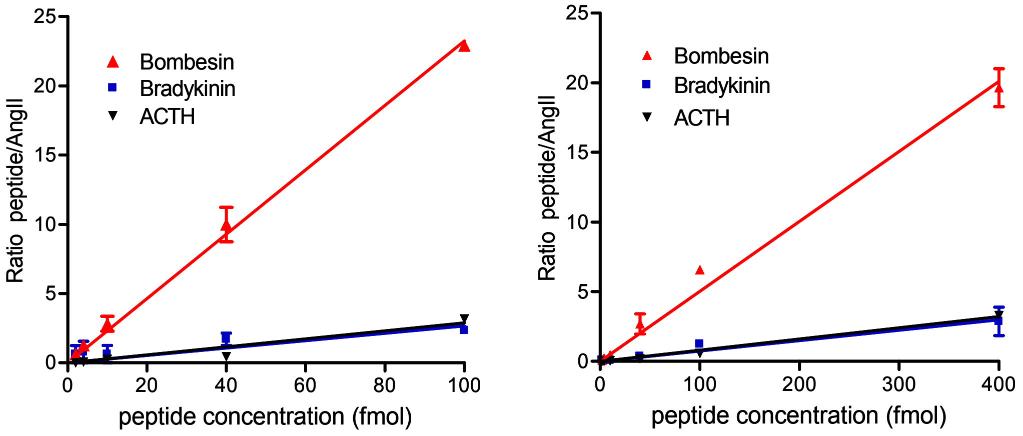

Figure S4. Quantification of the peptide intensities in the mass spectra from MALDI-TOF MS. Calibration experiments showing the linear correlation between peptide concentration and the ratio peptide intensity/angiotensin-II peptide intensity as relative intensity. Peptides used were bradykinin (765.85 Da), bombesin (1619.85 Da), and adrenocorticotropic hormone (ACTH, 2465.67 Da). Peptide intensities were obtained from mass spectra by MALDI-TOF MS analysis, in the same conditions as mass fingerprinting analysis and using angiotensin-II peptide (1045.53 Da) as internal standard. Peptide concentrations used were 2-100 fmol, and 4 fmol of angiotensin-II (left graph), or 4-400 fmol, with 40 fmol of angiotensin-II (right graph). The correlation between peptide concentration and relative intensity for each peptide was very significant, $p < 0.0005$ (Pearson test).

13. Supplementary Figure S5

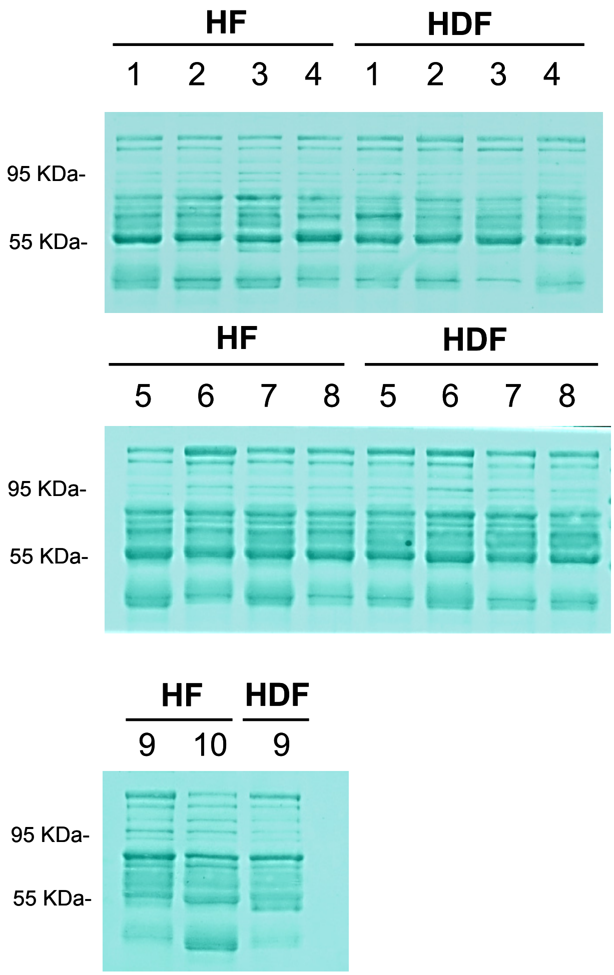

Figure S5. Loading control in western blot experiments for anti-TTR antibody detection. Blotted proteins were staining with Fast Green in the PVDF membranes as loading control of analyzed samples from HF and HDF patients. The upper piece of the membranes containing most proteins was used for staining and normalization dividing the TTR level of the western blot by the total level of stained proteins of the same loading lane. Images show stained whole blots.

15. Supplementary Figure S6

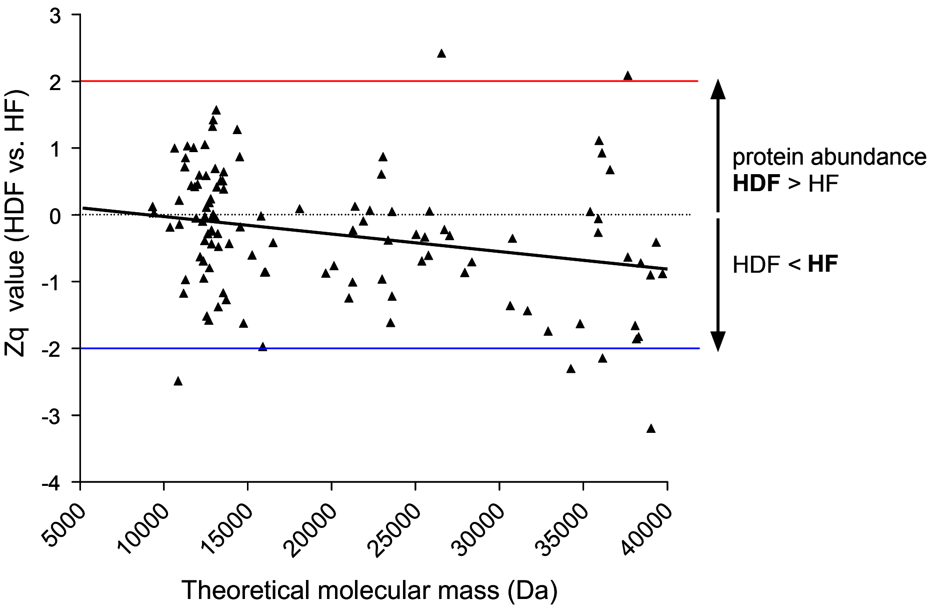

Figure S6. Correlation between the molecular mass (≤ 40 kDa) and the quantification value (Zq) in the proteins identified in plasma samples of HDF compared with HF patients by LC-MS/MS analysis. Protein quantification values (Zq) are the normalized log2-ratios expressed in standard deviation units: Zq > 0 and Zq < 0 indicate increased or decreased, respectively, protein abundance in HDF compared with HF patients. Zq ≥ 2, and ≤ -2, were significant ($p \leq 0.05$). Red and blue lines show the values Zq=2 and Zq= -2, respectively. The continuous black line shows de linear regression and correlation was significant ($p < 0.0043$, Pearson test).

16. MALDI-TOF MS spectra and Mascot searches

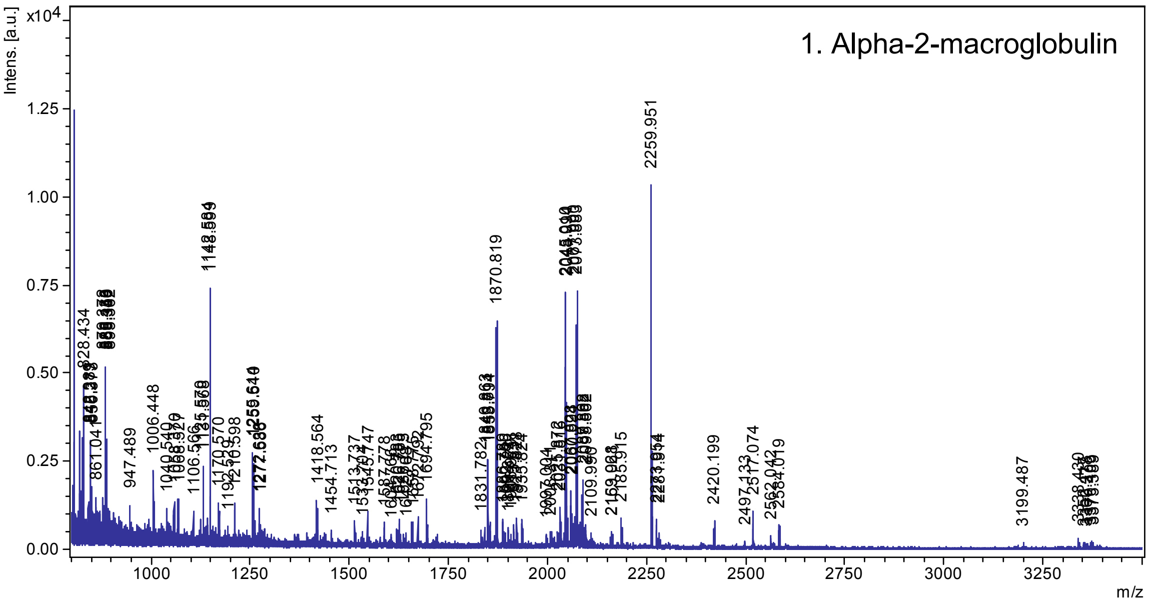

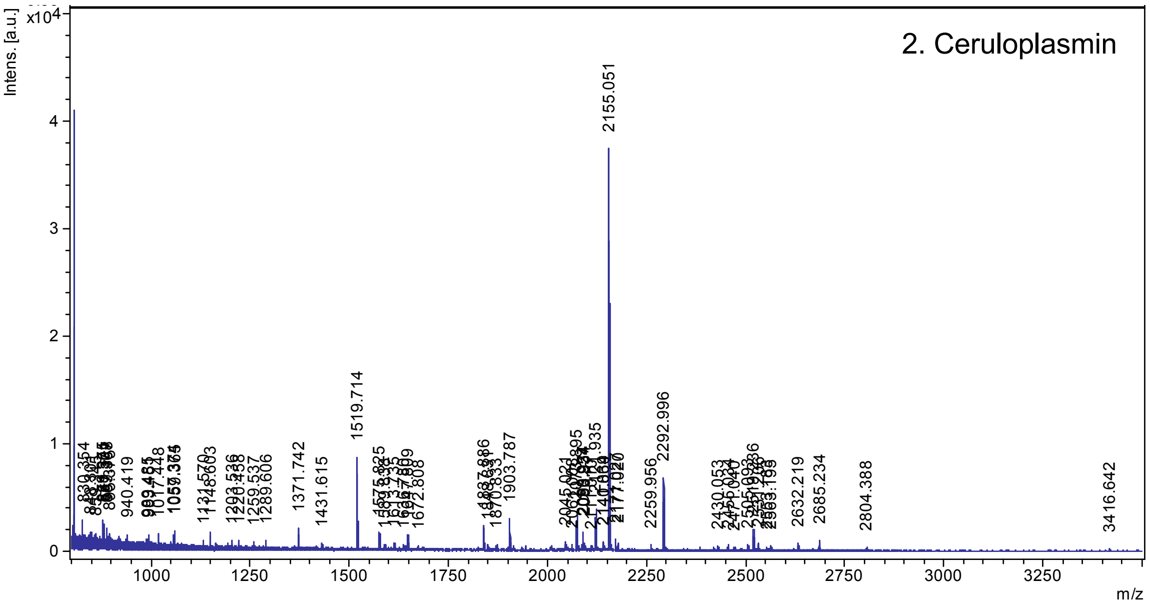

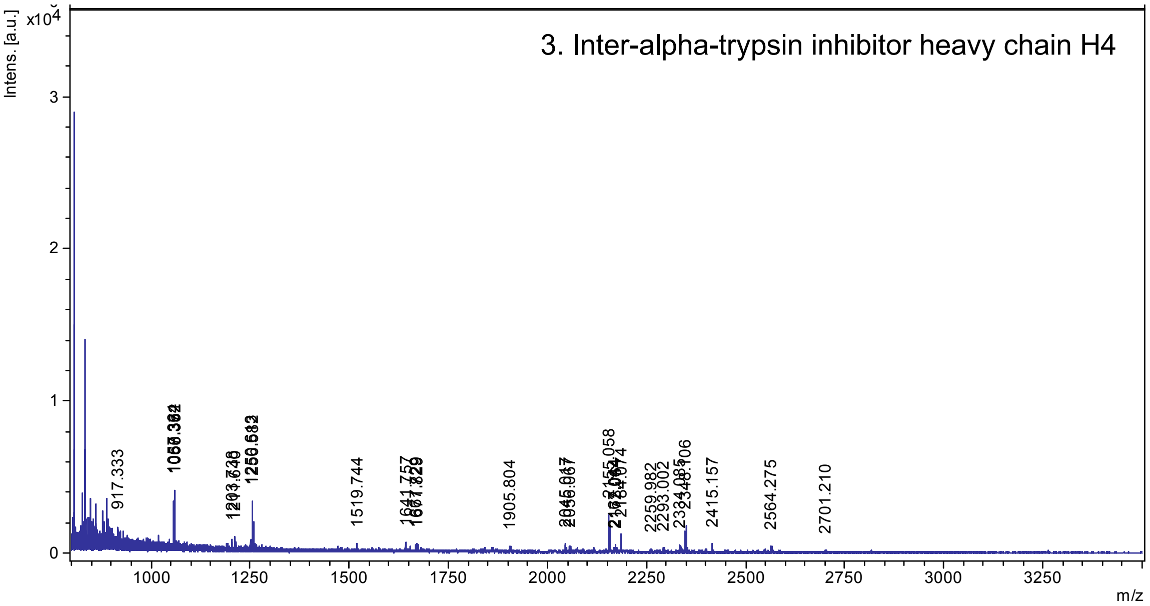

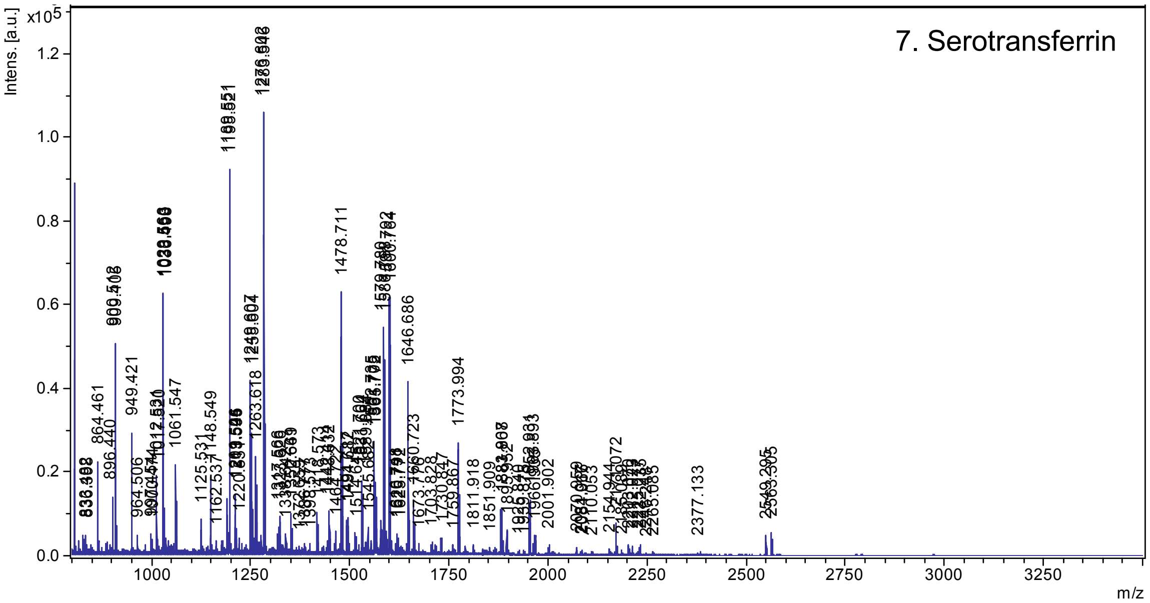
7. Serotransferrin
Intens. [a.u.]
x10^5
1.2
1.0
0.8
0.6
0.4
0.2
0.0
1000
1250
1500
1750
2000
2250
2500
2750
3000
3250
m/z
864.461
896.440
949.421
964.506
1061.547
1125.531
1148.549
1162.537
1263.618
1478.711
1646.686
1660.723
1773.994
1811.918
1851.909
2001.902
2110.053
2377.133
2549.295
2563.305

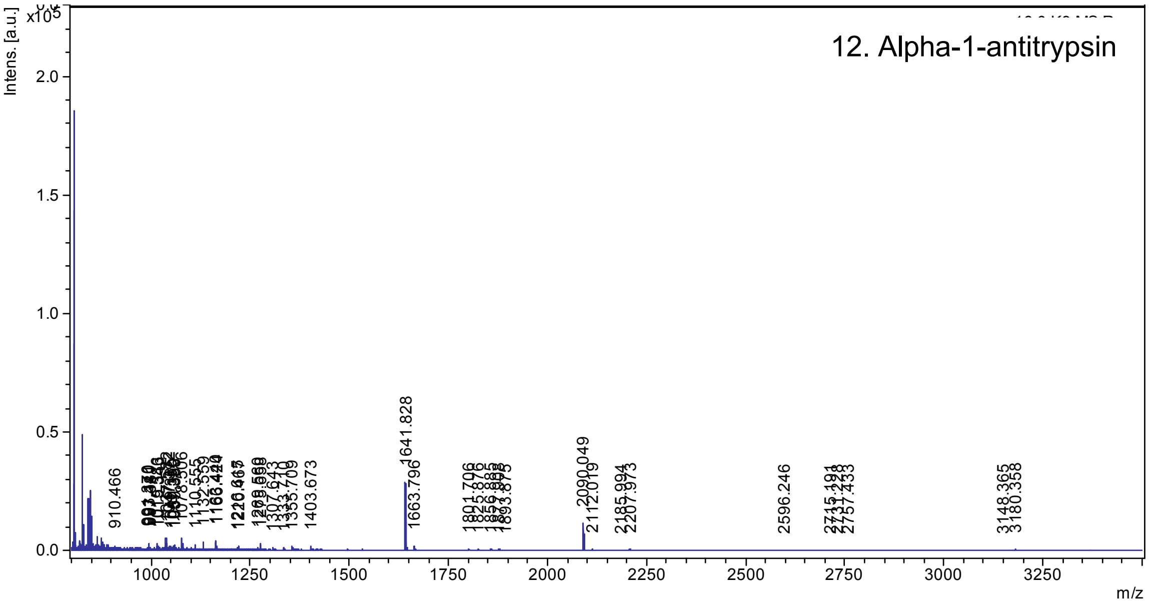
12. Alpha-1-antitrypsin
Intens. [a.u.]
x10^5
2.0
1.5
1.0
0.5
0.0
1000
1250
1500
1750
2000
2250
2500
2750
3000
3250
m/z
910.466
1403.673
1641.828
1663.796
1893.875
2090.049
2112.019
2185.994
2207.973
2596.246
2757.433
3148.365
3180.358

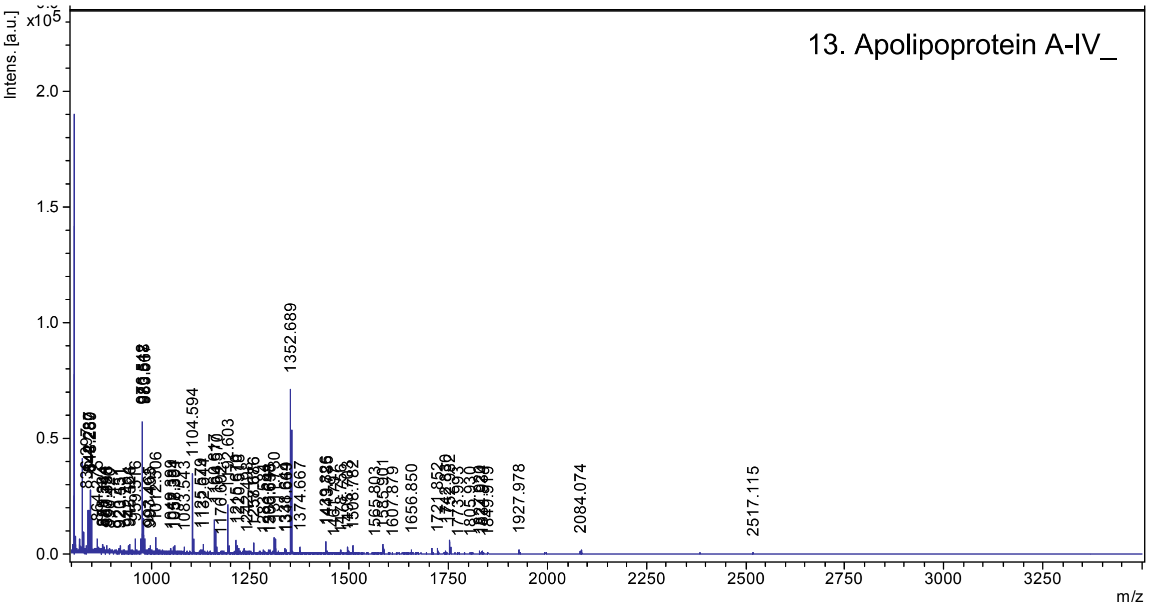
13. Apolipoprotein A-IV_
Intens. [a.u.]
x10^5
2.0
1.5
1.0
0.5
0.0
1000
1250
1500
1750
2000
2250
2500
2750
3000
3250
m/z
1104.594
1352.689
1374.667
1565.803
1656.850
1721.852
1927.978
2084.074
2517.115

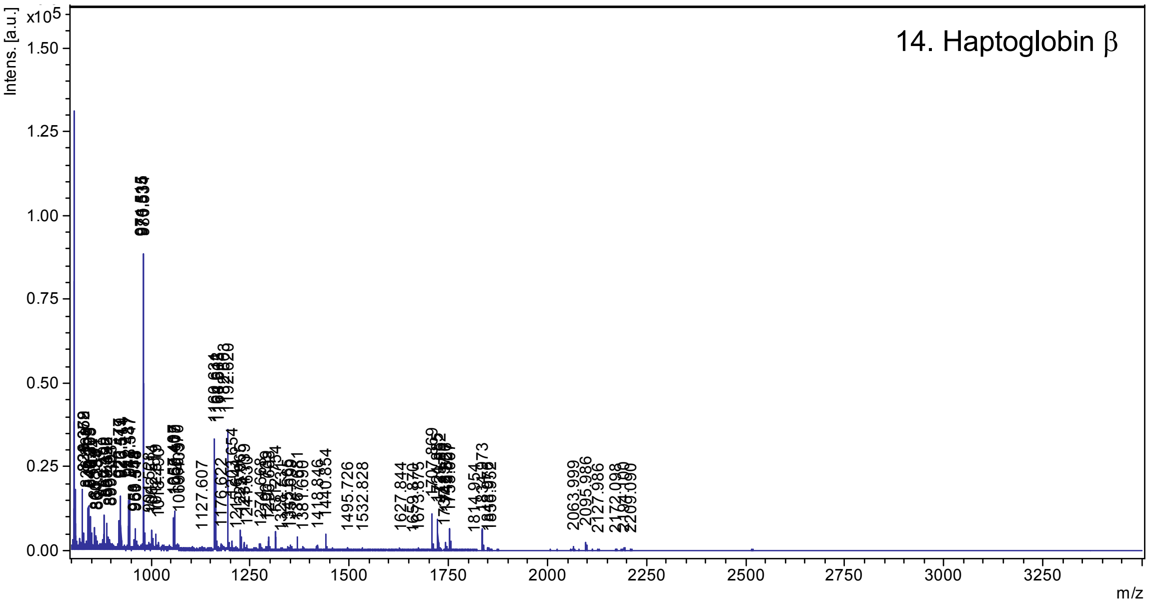
14. Haptoglobin β
Intens. [a.u.]
x10^5
m/z
980.535
1127.607
1495.726
1532.828
1627.844
2063.999
2095.986
2127.986

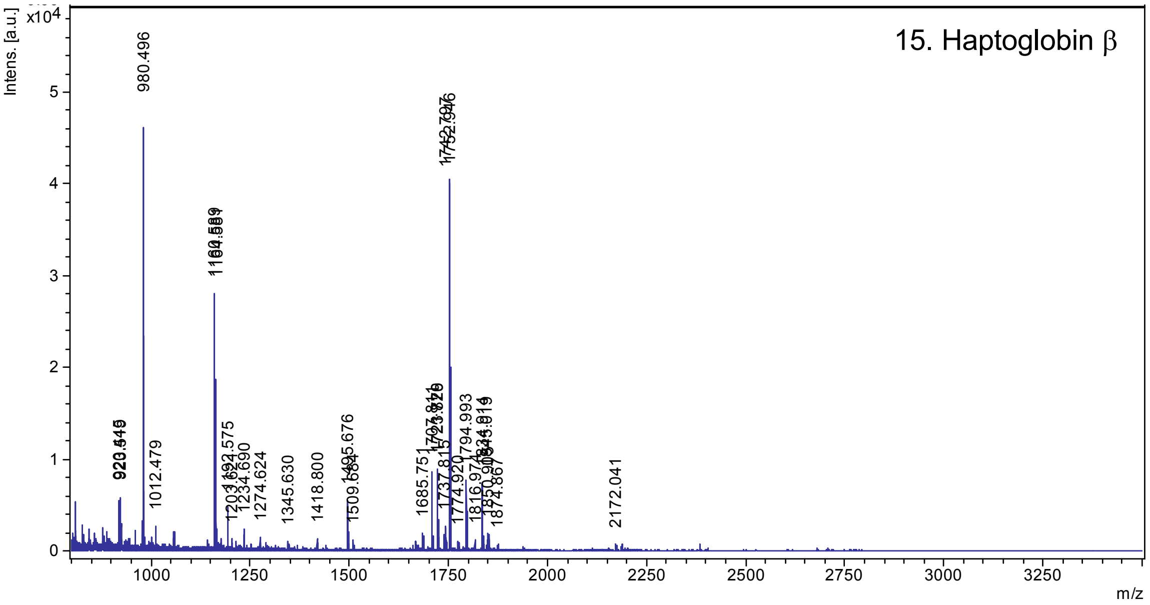
15. Haptoglobin β
Intens. [a.u.]
x10^4
m/z
980.496
1012.479
1234.690
1274.624
1345.630
1418.800
1495.676
1509.664
1685.751
1794.993
1874.867
2172.041

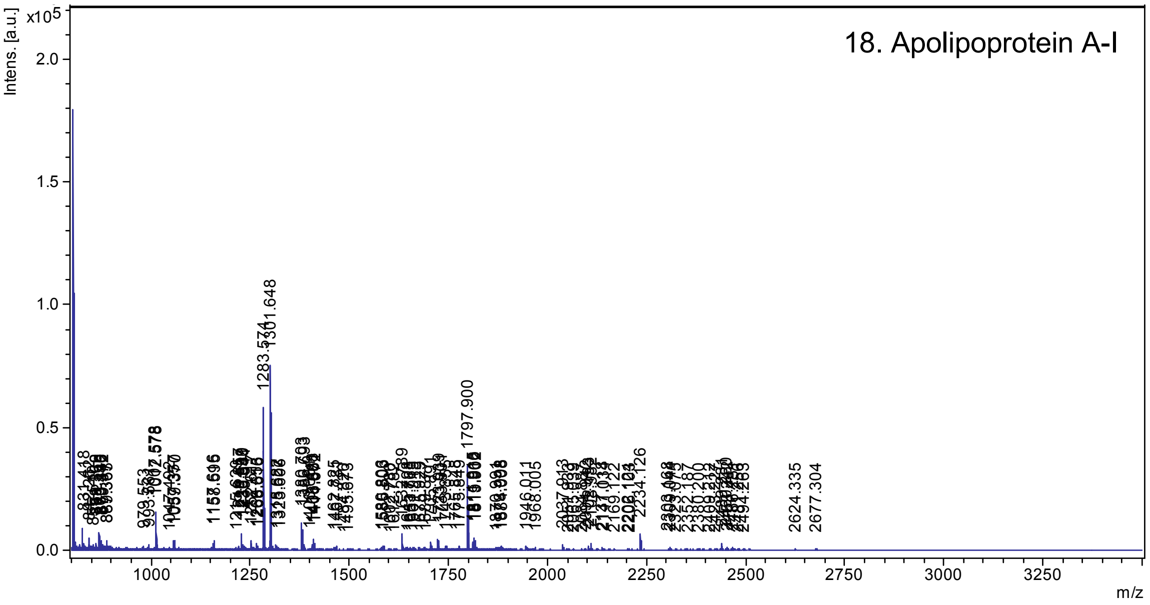
18. Apolipoprotein A-I
Intens. [a.u.]
x10^5
m/z
1301.648
1283.574
1797.900
1946.011
1968.005
2234.126
2624.335
2677.304

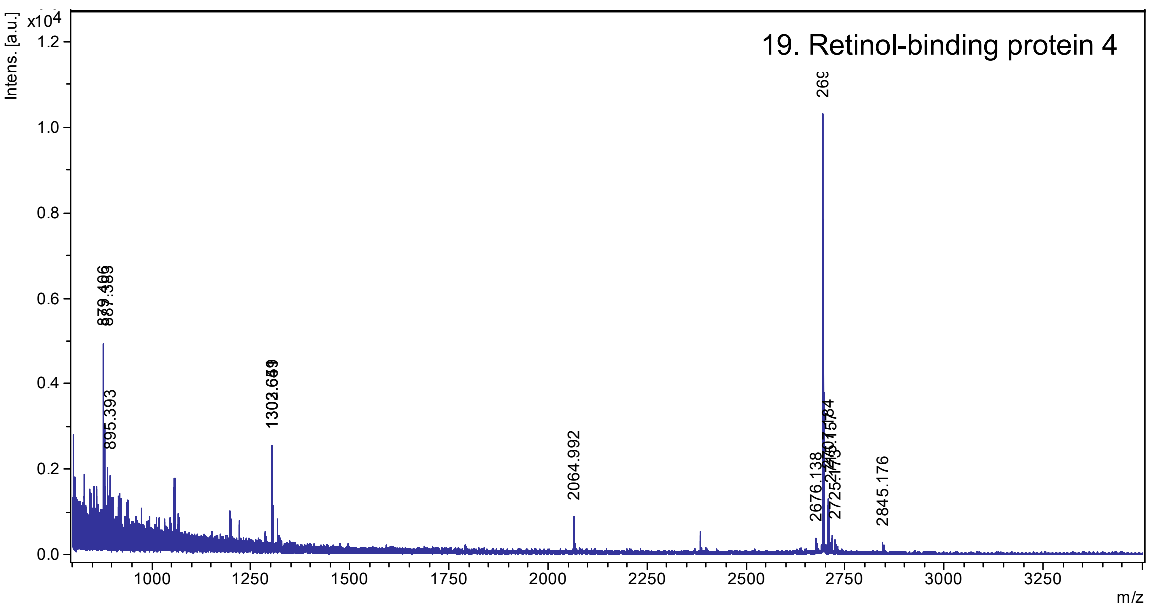
19. Retinol-binding protein 4
Intens. [a.u.]
x10^4
895.393
1302.659
2064.992
2676.138
2845.176
m/z

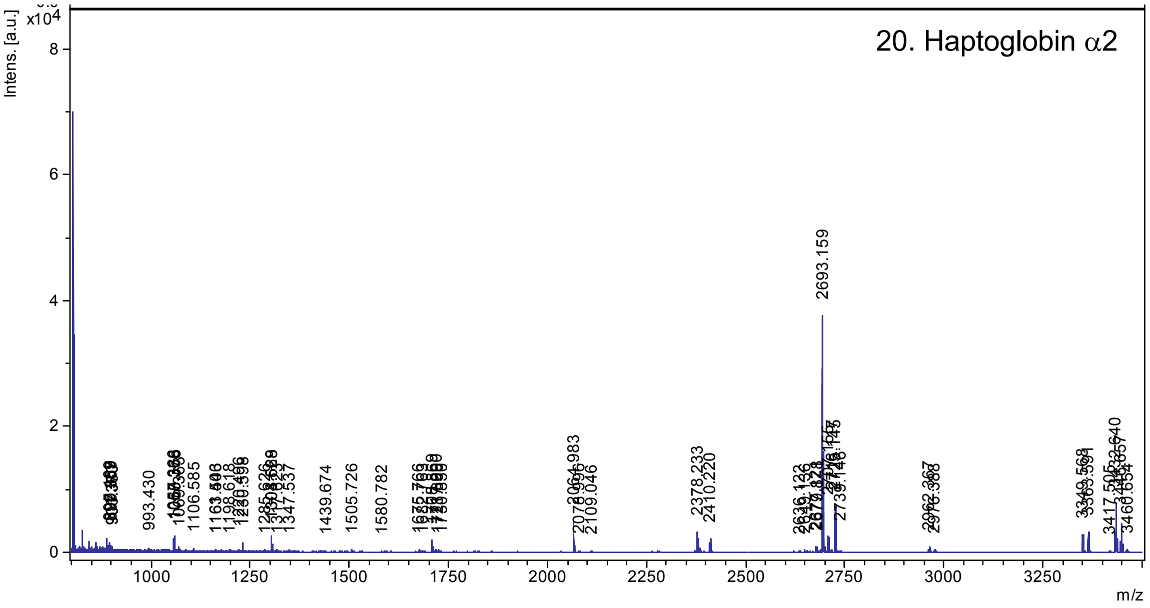
20. Haptoglobin α2
Intens. [a.u.]
x10^4
2693.159
993.430
1106.585
1439.674
1505.726
1580.782
2064.983
2109.046
2378.233
2410.220
m/z

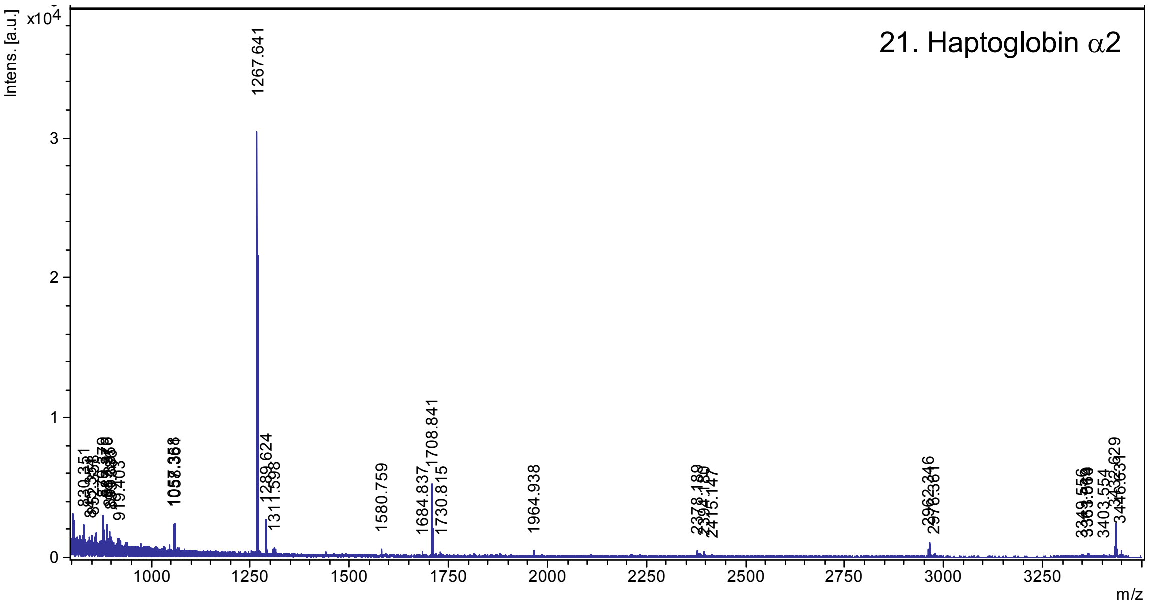
21. Haptoglobin α2
Intens. [a.u.]
x10^4
1267.641
1289.624
1311.598
1580.759
1684.837
1708.841
1730.815
1964.938
2415.147
3403.554
m/z

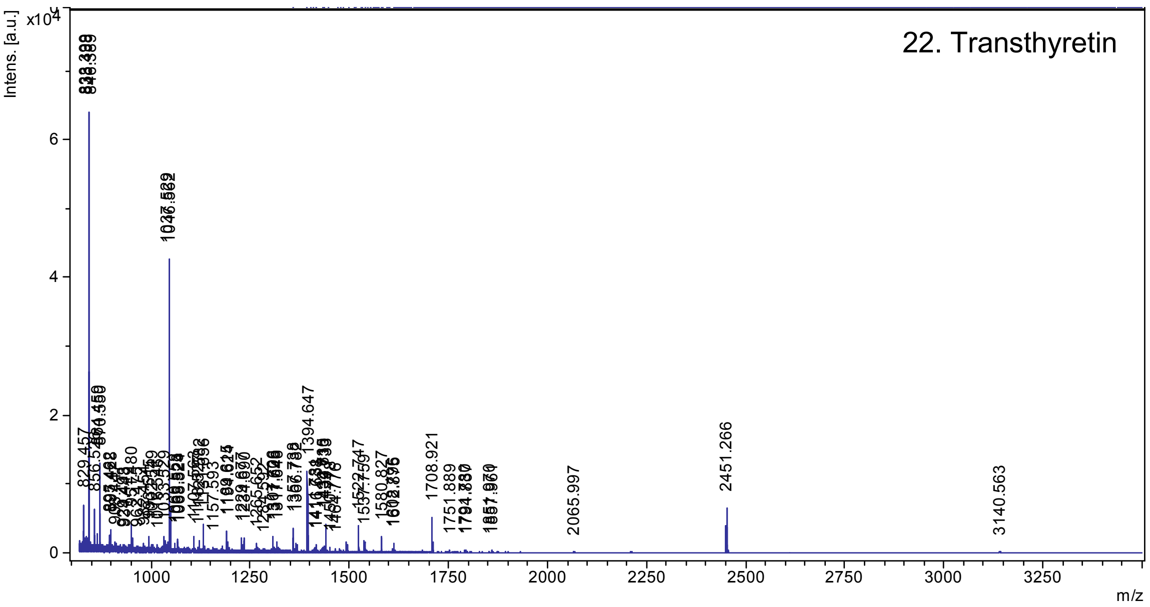
22. Transthyretin
Intens. [a.u.]
x10^4
m/z
1708.921
2065.997
2451.266
3140.563
1394.647

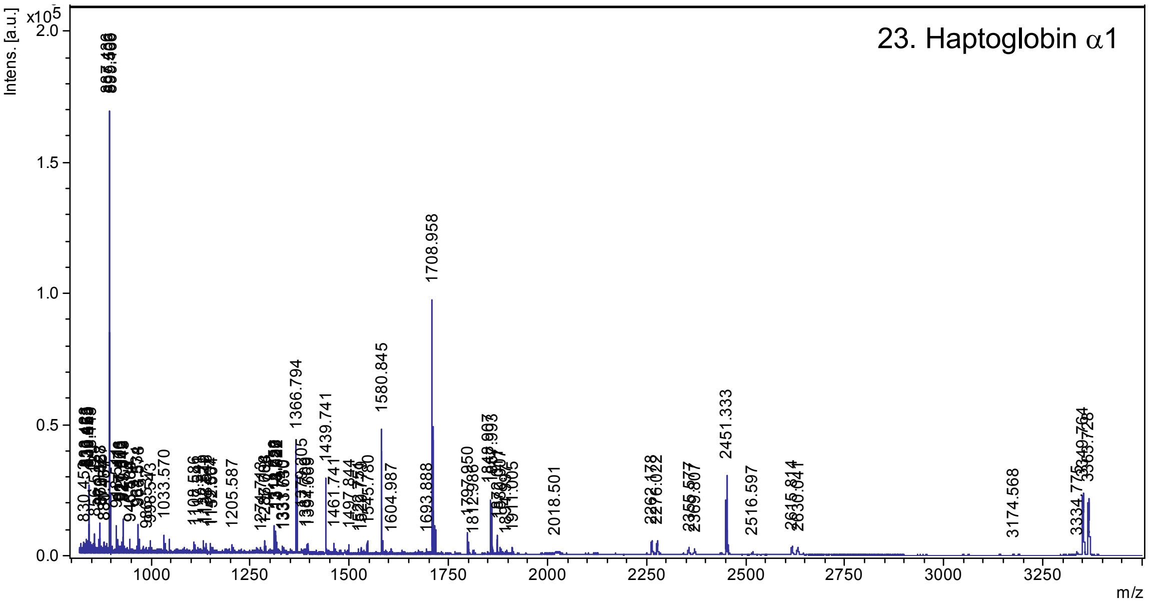
23. Haptoglobin α1
Intens. [a.u.]
x10^5
m/z
1708.958
1580.845
1366.794
1439.741
2451.333
3174.568
2018.501
2516.597

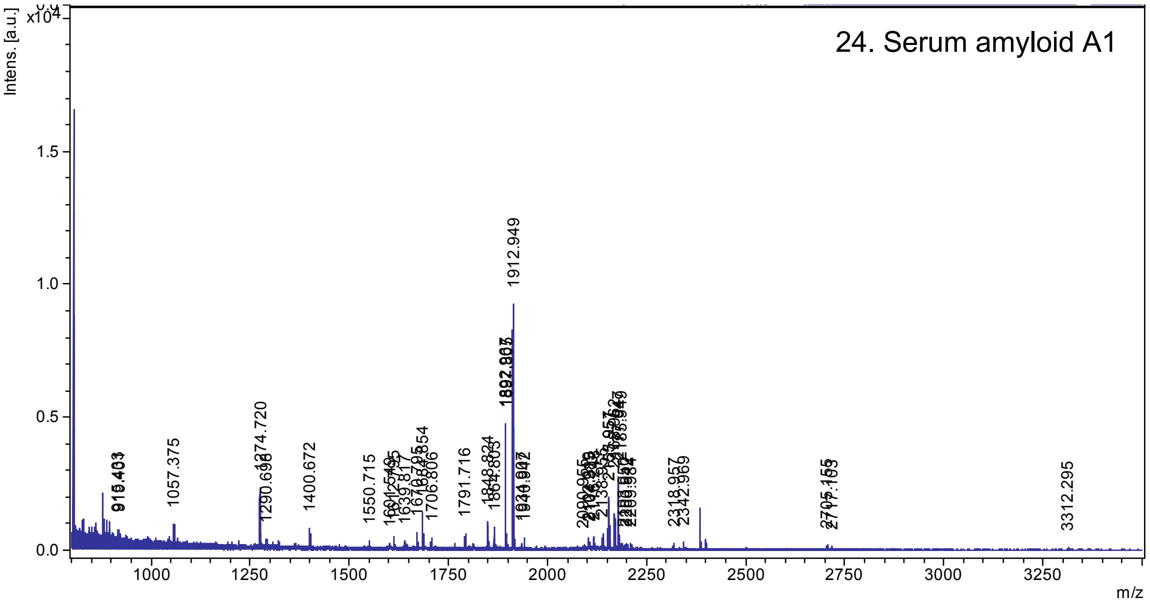
24. Serum amyloid A1
Intens. [a.u.]
x10^4
m/z
1912.949
1274.720
1057.375
1400.672
1550.715
1791.716
3312.295
2342.969

MATRIX SCIENCE MASCOT Search Results

Protein View: A2MG_HUMAN

Alpha-2-macroglobulin OS=Homo sapiens OX=9606 GN=A2M PE=1 SV=3

**Database:**	SwissProt
**Score:**	95
**Expect:**	6e-06
**Monoisotopic mass (M_r):**	164613
**Calculated pI:**	6.03
**Taxonomy:**	Homo sapiens

Sequence similarity is available as an NCBI BLAST search of A2MG_HUMAN against nr.

Search parameters

**Enzyme:**	Trypsin: cuts C-term side of KR unless next residue is P.
**Fixed modifications:**	Carbamidomethyl (C)
**Variable modifications:**	Oxidation (M)
**Mass values searched:**	86
**Mass values matched:**	24

Protein sequence coverage: 20%

Matched peptides shown in ***bold red***.

1	MGKNKLLHPS	LVLLLLVLLP	TDASVSGKPQ	YMVLVPSLLH	TETTEKGCVL
51	LSYLNETVTV	SASLESVRGN	RSLFTDLEAE	NDVLHCVAFA	VPKSSSNEEV
101	MFLTVQVKGP	TQEFKKRTTV	MVKNEDSLVF	VQTDKSIYKP	GQTVKFRVVS
151	MDENFHPLNE	LIPLVYIQDP	KGNR**IAQWQS**	**FQLEGGLKQF**	**SFPLSSEPFQ**
201	**GSYK**VVVQKK	SGGR**TEHPFT**	**VEEFVLPK**FE	VQVTVPKIIT	ILEEEMNVSV
251	CGLYTYGKPV	PGHVTVSICR	KYSDASDCHG	EDSQAFCEK**F**	**SGQLNSHGCF**
301	**YQQVK**TKVFQ	LKRKEYEMK**L**	**HTEAQIQEEG**	**TVVELTGRQS**	**SEITR**TITKL
351	SFVKVDSHFR	**QGIPFFGQVR**	LVDGKGVPIP	NKVIFIRGNE	ANYYSNATTD
401	EHGLVQFSIN	TTNVMGTSLT	VRVNYKDR**SP**	**CYGYQWVSEE**	**HEEAHHTAYL**
451	**VFSPSK**SFVH	LEPMSHELPC	GHTQTVQAHY	ILNGGTLLGL	KKLSFYYLIM
501	AKGGIVRTGT	HGLLVKQEDM	KGHFSISIPV	K**SDIAPVAR**L	LIYAVLPTGD
551	VIGDSAKYDV	ENCLANK**VDL**	**SFSPSQSLPA**	**SHAHLRVTAA**	**PQSVCALR**AV
601	DQSVLLMKPD	AELSASSVYN	LLPEKDLTGF	PGPLNDQDNE	DCINRHNVYI
651	NGITYTPVSS	TNEKDMYSFL	EDMGLKAFTN	SKIRKPK**MCP**	**QLQQYEMHGP**
701	**EGLRVGFYES**	**DVMGR**GHAR**L**	**VHVEEPHTET**	**VR**KYFPETWI	WDLVVVNSAG
751	VAEVGVTVPD	TITEWKAGAF	CLSEDAGLGI	SSTASLR**AFQ**	**PFFVELTMPY**
801	**SVIR**GEAFTL	KATVLNYLPK	CIRVSVQLEA	SPAFLAVPVE	KEQAPHCICA
851	NGRQTVSWAV	TPKSLGNVNF	TVSAEALESQ	ELCGTEVPSV	PEHGRKDTVI
901	KPLLVEPEGL	EKETTFNSLL	CPSGGEVSEE	LSLK**LPPNVV**	**EESAR**ASVSV
951	LGDILGSAMQ	NTQNLLQMPY	GCGEQNMVLF	APNIYVLDYL	NETQQLTPEI
1001	KSK**AIGYLNT**	**GYQR**QLNYK**H**	**YDGSYSTFGE**	**R**YGRNQGNTW	LTAFVLKTFA
1051	QARAYIFIDE	AHITQALIWL	SQRQKDNGCF	RSSGSLLNNA	IKGGVEDEVT
1101	LSAYITIALL	EIPLTVTHPV	VRNALFCLES	AWKTAQEGDH	GSHVYTKALL
1151	AYAFALAGNQ	DKRKEVLKSL	NEEAVKK**DNS**	**VHWERPQKPK**	APVGHFYEPQ

1201 APSAEVEMTS YVLLAYLTAQ PAPTSEDLTS ATNIVKWITK QQNAQGGFSS
1251 TQDTVVALHA LSK**YGAATFT** **R**TGKAAQVTI QSSGTFSSK**F** **QVDNNNR**LLL
1301 QQVSLPELPG EYSMKVTGEG CVYLQTSLKY NILPEKEEFP FALGVQTLPQ
1351 TCDEPK**AHTS** **FQISLSVSYT** **GSR**SASNMAI VDVKMVSGFI PLKPTVKMLE
1401 RSNHVSRTEV SSNHVLIYLD KVSNQTLSLF FTVLQDVPVR DLKPAIVKVY
1451 DYYETDEFAI AEYNAPCSKD LGNA

Unformatted sequence string: 1474 residues (for pasting into other applications).

Sort by ◉ residue number ○ increasing mass ○ decreasing mass
Show ◉ matched peptides only ○ predicted peptides also

Start – End	Observed	Mr(expt)	Mr(calc)	ppm	M	Peptide
175 – 188	1604.7657	1603.7584	1603.8358	-48.3	0	R.IAQWQSFQLEGGLK.Q
189 – 204	1848.8113	1847.8040	1847.8730	-37.3	0	K.QFSFPLSSEPFQGSYK.V
215 – 228	1672.7917	1671.7844	1671.8508	-39.7	0	R.TEHPFTVEEFVLPK.F
290 – 305	1899.8224	1898.8151	1898.8734	-30.7	0	K.FSGQLNSHGCFYQQVK.T
320 – 338	2109.9903	2108.9831	2109.0702	-41.3	0	K.LHTEAQIQEEGTVVELTGF
339 – 345	820.4113	819.4040	819.4086	-5.59	0	R.QSSEITR.T
361 – 370	1148.5988	1147.5916	1147.6138	-19.4	0	R.QGIPFFGQVR.L
429 – 456	3338.4298	3337.4225	3337.4775	-16.5	0	R.SPCYGYQWVSEEHEEAHHT
532 – 539	828.4336	827.4263	827.4501	-28.7	0	K.SDIAPVAR.L
568 – 586	2048.9940	2047.9867	2048.0439	-27.9	0	K.VDLSFSPSQSLPASHAHLF
587 – 598	1272.6381	1271.6308	1271.6656	-27.4	0	R.VTAAPQSVCALR.A
688 – 704	2073.8831	2072.8758	2072.9230	-22.8	0	K.MCPQLQQYEMHGPEGLR.V
688 – 704	2089.8621	2088.8548	2088.9179	-30.2	0	K.MCPQLQQYEMHGPEGLR.V
705 – 715	1259.5404	1258.5331	1258.5652	-25.5	0	R.VGFYESDVMGR.G
720 – 732	1545.7465	1544.7393	1544.7947	-35.9	0	R.LVHVEEPHTETVR.K
788 – 804	2045.0104	2044.0031	2044.0492	-22.6	0	R.AFQPFFVELTMPYSVIR.G
788 – 804	2060.9944	2059.9872	2060.0441	-27.6	0	R.AFQPFFVELTMPYSVIR.G
935 – 945	1210.5984	1209.5911	1209.6353	-36.5	0	K.LPPNVVEESAR.A
1004 – 1014	1255.6135	1254.6063	1254.6357	-23.4	0	K.AIGYLNTGYQR.Q
1020 – 1031	1418.5643	1417.5570	1417.5899	-23.2	0	K.HYDGSYSTFGER.Y
1178 – 1190	1620.7833	1619.7761	1619.8168	-25.2	0	K.DNSVHWERPQKPK.A
1264 – 1271	886.4327	885.4254	885.4345	-10.2	0	K.YGAATFTR.T
1290 – 1297	1006.4483	1005.4410	1005.4628	-21.6	0	K.FQVDNNNR.L
1357 – 1373	1840.8629	1839.8556	1839.9115	-30.4	0	K.AHTSFQISLSVSYTGSR.S

No match to: 826.2877, 842.3894, 848.2769, 850.3727, 861.0413, 879.3721, 888.3465, 889.3590, 895.3618, 947.4890, 1040.5396, 1058.3703, 1068.5270, 1106.5661, 1125.5697, 1131.5676, 1142.5836, 1170.5696, 1192.5295, 1277.5860, 1454.7129, 1513.7372, 1533.7041, 1587.7778, 1626.7851, 1636.7750, 1642.7680, 1658.7747, 1694.7954, 1831.7819, 1853.7943, 1870.8189, 1886.7886, 1892.7857, 1905.9787, 1913.8309, 1921.8281, 1935.8236, 1997.0044, 2008.3115, 2025.8719, 2031.8163, 2057.8285, 2067.0004, 2087.8984, 2095.8619, 2159.9278, 2163.0614, 2185.9147, 2259.9510, 2273.9541, 2281.9145, 2420.1989, 2497.1328, 2517.0739, 2562.0416, 2584.0188, 3199.4870, 3352.4173, 3360.3783, 3370.4030, 3375.3890

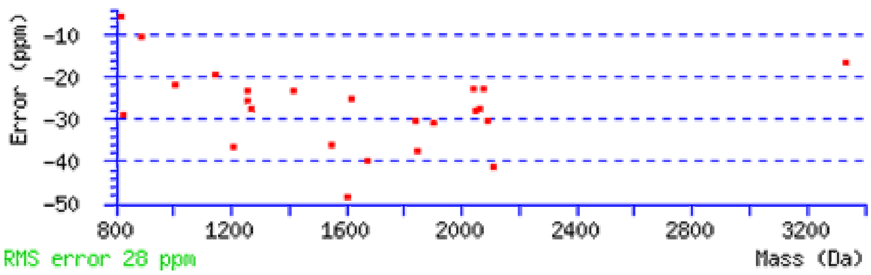

MATRIX SCIENCE MASCOT Search Results

Protein View: CERU_HUMAN

Ceruloplasmin OS=Homo sapiens OX=9606 GN=CP PE=1 SV=1

**Database:**	SwissProt
**Score:**	112
**Expect:**	1.3e-07
**Monoisotopic mass (M_r):**	122983
**Calculated pI:**	5.44
**Taxonomy:**	Homo sapiens

Sequence similarity is available as an NCBI BLAST search of CERU_HUMAN against nr.

Search parameters

**Enzyme:**	Trypsin: cuts C-term side of KR unless next residue is P.
**Fixed modifications:**	Carbamidomethyl (C)
**Variable modifications:**	Oxidation (M)
**Mass values searched:**	66
**Mass values matched:**	19

Protein sequence coverage: 24%

Matched peptides shown in ***bold red***.

1	MKILILGIFL	FLCSTPAWAK	EKHYYIGIIE	TTWDYASDHG	EKK**LISVDTE**
51	**HSNIYLQNGP**	**DR**IGRLYK**KA**	**LYLQYTDETF**	**R**TTIEKPVWL	GFLGPIIKAE
101	TGDKVYVHLK	**NLASRPYTFH**	**SHGITYYK**EH	EGAIYPDNTT	DFQR**ADDKVY**
151	**PGEQYTYMLL**	**ATEEQSPGEG**	**DGNCVTR**IYH	SHIDAPKDIA	SGLIGPLIIC
201	KKDSLDKEKE	KHIDREFVVM	FSVVDENFSW	YLEDNIKTYC	SEPEKVDKDN
251	EDFQESNR**MY**	**SVNGYTFGSL**	**PGLSMCAEDR**	VKWYLFGMGN	EVDVHAAFFH
301	GQALTNKNYR	IDTINLFPAT	LFDAYMVAQN	PGEWMLSCQN	LNHLKAGLQA
351	FFQVQECNKS	SSKDNIRGKH	VRHYYIAAEE	IIWNYAPSGI	DIFTKENLTA
401	PGSDSAVFFE	QGTTRIGGSY	KKLVYR**EYTD**	**ASFTNR**KERG	PEEEHLGILG
451	PVIWAEVGDT	IRVTFHNK**GA**	**YPLSIEPIGV**	**RFNKNNEGTY**	**YSPNYNPQSR**
501	**SVPPSASHVA**	**PTETFTYEWT**	**VPK**EVGPTNA	DPVCLAKMYY	SAVDPTKDIF
551	TGLIGPMKIC	KK**GSLHANGR**	QKDVDKEFYL	FPTVFDENES	LLLEDNIRMF
601	TTAPDQVDKE	DEDFQESNKM	HSMNGFMYGN	QPGLTMCKGD	SVVWYLFSAG
651	NEADVHGIYF	SGNTYLWRGE	RRDTANLFPQ	TSLTLHMWPD	TEGTFNVECL
701	TTDHYTGGMK	QK**YTVNQCR**R	**QSEDSTFYLG**	**ER**TYYIAAVE	VEWDYSPQRE
751	WEKELHHLQE	QNVSNAFLDK	GEFYIGSKYK	KVVYR**QYTDS**	**TFR**VPVERKA
801	EEEHLGILGP	QLHADVGDKV	KIIFKNMATR	PYSIHAHGVQ	TESSTVTPTL
851	PGETLTYVWK	IPER**SGAGTE**	**DSACIPWAYY**	**STVDQVKDLY**	**SGLIGPLIVC**
901	**R**RPYLKVFNP	RRKLEFALLF	LVFDENESWY	LDDNIKTYSD	HPEKVNKDDE
951	EFIESNKMHA	INGRMFGNLQ	GLTMHVGDEV	NWYLMGMGNE	IDLHTVHFHG
1001	HSFQYKHR**GV**	**YSSDVFDIFP**	**GTYQTLEMFP**	**R**TPGIWLLHC	HVTDHIHAGM
1051	ETTYTVLQNE	DTKSG			

Unformatted sequence string: 1065 residues (for pasting into other applications).

Sort by ◉ residue number ○ increasing mass ○ decreasing mass

Show ◉ matched peptides only ○ predicted peptides also

Start - End	Observed	Mr(expt)	Mr(calc)	ppm	M	Peptide
44 - 62	2171.0270	2170.0197	2170.0654	-21.1	0	K.LISVDTEHSNIYLQNGPDF
69 - 81	1647.8086	1646.8013	1646.8304	-17.7	1	K.KALYLQYTDETFR.T
70 - 81	1519.7144	1518.7072	1518.7354	-18.6	0	K.ALYLQYTDETFR.T
111 - 128	2155.0509	2154.0436	2154.0647	-9.77	0	K.NLASRPYTFHSHGITYYK.
145 - 177	3693.5792	3692.5720	3692.6247	-14.3	1	R.ADDKVYPGEQYTYMLLATE
259 - 280	2455.0337	2454.0264	2454.0654	-15.9	0	R.MYSVNGYTFGSLPGLSMCA
259 - 280	2471.0401	2470.0328	2470.0603	-11.1	0	R.MYSVNGYTFGSLPGLSMCA
427 - 436	1203.5360	1202.5287	1202.5204	6.91	0	R.EYTDASFTNR.K
469 - 481	1371.7425	1370.7352	1370.7558	-15.0	0	K.GAYPLSIEPIGVR.F
482 - 500	2292.9955	2291.9882	2292.0195	-13.6	1	R.FNKNNEGTYYSPNYNPQSR
485 - 500	1903.7868	1902.7795	1902.8132	-17.7	0	K.NNEGTYYSPNYNPQSR.S
501 - 523	2531.2065	2530.1992	2530.2380	-15.3	0	R.SVPPSASHVAPTETFTYEW
563 - 570	811.4003	810.3930	810.4096	-20.5	0	K.GSLHANGR.Q
713 - 719	940.4193	939.4120	939.4232	-11.9	0	K.YTVNQCR.R
721 - 732	1431.6146	1430.6073	1430.6314	-16.8	0	R.QSEDSTFYLGER.T
786 - 793	1017.4482	1016.4409	1016.4563	-15.2	0	R.QYTDSTFR.V
865 - 887	2505.0933	2504.0860	2504.1166	-12.2	0	R.SGAGTEDSACIPWAYYSTV
888 - 901	1575.8252	1574.8179	1574.8490	-19.8	0	K.DLYSGLIGPLIVCR.R
1009 - 1031	2685.2344	2684.2271	2684.2469	-7.37	0	R.GVYSSDVFDIFPGTYQTLE

No match to: 826.2964, 830.3540, 848.3008, 859.3449, 879.3752, 881.3009, 887.3666, 889.3685, 895.3748, 989.4849, 993.4508, 1057.3741, 1059.3650, 1131.5696, 1148.6031, 1220.4582, 1259.5374, 1289.6064, 1589.8379, 1613.7348, 1636.7803, 1672.8079, 1837.8858, 1848.8312, 1870.8327, 2045.0215, 2061.0078, 2073.8950, 2088.9267, 2090.9538, 2093.9340, 2111.0095, 2121.9353, 2140.0393, 2141.8642, 2177.0200, 2259.9564, 2430.0527, 2519.2360, 2553.1833, 2563.1952, 2632.2186, 2804.3878, 3416.6424, 3487.5071, 3707.5985, 3867.3625

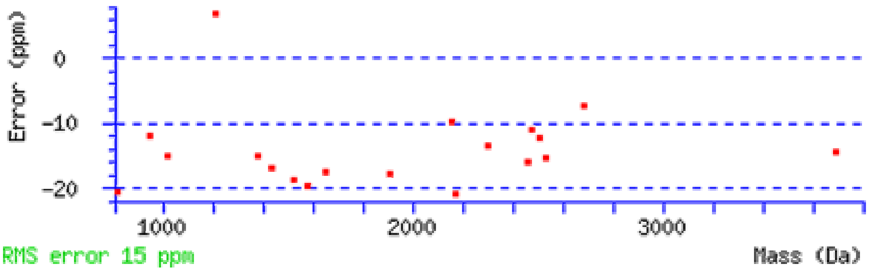

MATRIX SCIENCE MASCOT Search Results

Protein View: ITIH4_HUMAN

Inter-alpha-trypsin inhibitor heavy chain H4 OS=Homo sapiens OX=9606 GN=ITIH4 PE=1 SV=4

**Database:**	SwissProt
**Score:**	48
**Expect:**	0.33
**Monoisotopic mass (M_r):**	103521
**Calculated pI:**	6.51
**Taxonomy:**	Homo sapiens

Sequence similarity is available as an NCBI BLAST search of ITIH4_HUMAN against nr.

Search parameters

**Enzyme:**	Trypsin: cuts C-term side of KR unless next residue is P.
**Fixed modifications:**	Carbamidomethyl (C)
**Variable modifications:**	Oxidation (M)
**Mass values searched:**	26
**Mass values matched:**	6

Protein sequence coverage: 10%

Matched peptides shown in ***bold red***.

1	MKPPRPVRTC	SKVLVLLSLL	AIHQTTTAEK	NGIDIYSLTV	DSRVSSRFAH
51	TVVTSRVVNR	ANTVQEATFQ	MELPKKAFIT	NFSMIIDGMT	YPGIIKEKAE
101	AQAQYSAAVA	KGKSAGLVKA	TGRNMEQFQV	SVSVAPNAKI	TFELVYEELL
151	KR**RLGVYELL**	**LK**VRPQQLVK	HLQMDIHIFE	PQGISFLETE	STFMTNQLVD
201	ALTTWQNKTK	AHIRFKPTLS	QQQKSPEQQE	TVLDGNLIIR	YDVDRAISGG
251	SIQIENGYFV	HYFAPEGLTT	MPKNVVFVID	KSGSMSGRKI	QQTREALIKI
301	LDDLSPRDQF	NLIVFSTEAT	QWRPSLVPAS	AENVNKARSF	AAGIQALGGT
351	NINDAMLMAV	QLLDSSNQEE	RLPEGSVSLI	ILLTDGDPTV	GETNPRSIQN
401	NVREAVSGRY	SLFCLGFGFD	VSYAFLEKLA	LDNGGLARRI	HEDSDSALQL
451	QDFYQEVANP	LLTAVTFEYP	SNAVEEVTQN	NFRLLFKGSE	MVVAGKLQDR
501	GPDVLTATVS	GKLPTQNITF	QTESSVAEQE	AEFQSPK**YIF**	**HNFMER**LWAY
551	LTIQQLLEQT	VSASDADQQA	LRNQALNLSL	AYSFVTPLTS	MVVTKPDDQE
601	QSQVAEKPME	GESRNRNVHS	GSTFFKYYLQ	GAKIPKPEAS	FSPRRGWNRQ
651	AGAAGSRMNF	RPGVLSSR**QL**	**GLPGPPDVPD**	**HAAYHPFR**R**L**	**AILPASAPPA**
701	**TSNPDPAVSR**	VMNMKIEETT	MTTQTPAPIQ	APSAILPLPG	QSVERLCVDP
751	RHR**QGPVNLL**	**SDPEQGVEVT**	**GQYER**EKAGF	SWIEVTFKNP	LVWVHASPEH
801	VVVTRNRRSS	AYKWKETLFS	VMPGLKMTMD	KTGLLLLSDP	DKVTIGLLFW
851	DGRGEGLRLL	LRDTDRFSSH	VGGTLGQFYQ	EVLWGSPAAS	DDGRRTLRVQ
901	GNDHSATRER	**RLDYQEGPPG**	**VEISCWSVEL**		

Unformatted sequence string: 930 residues (for pasting into other applications).

Sort by	◉ residue number	○ increasing mass	○ decreasing mass
Show	◉ matched peptides only	○ predicted peptides also	

Start - End	Observed	Mr(expt)	Mr(calc)	ppm	M	Peptide
153 - 162	1203.7383	1202.7310	1202.7387	-6.36	1	R.RLGVYELLLK.V
538 - 546	1256.5818	1255.5745	1255.5808	-4.99	0	K.YIFHNFMER.L
669 - 688	2184.0738	2183.0666	2183.0912	-11.3	0	R.QLGLPGPPDVPDHAAYHPF
690 - 710	2045.0168	2044.0095	2044.0953	-42.0	0	R.LAILPASAPPATSNPDPAV
754 - 775	2415.1570	2414.1497	2414.1714	-8.98	0	R.QGPVNLLSDPEQGVEVTGQ
911 - 930	2334.0845	2333.0772	2333.0998	-9.67	1	R.RLDYQEGPPGVEISCWSVE

No match to: 917.3328, 1057.3640, 1058.3722, 1060.3816, 1211.6404, 1250.6130, 1519.7444, 1641.7572, 1667.7294, 1671.8289, 1905.8039, 2056.0675, 2155.0575, 2167.0439, 2172.0641, 2259.9816, 2293.0024, 2348.1058, 2564.2746, 2701.2096

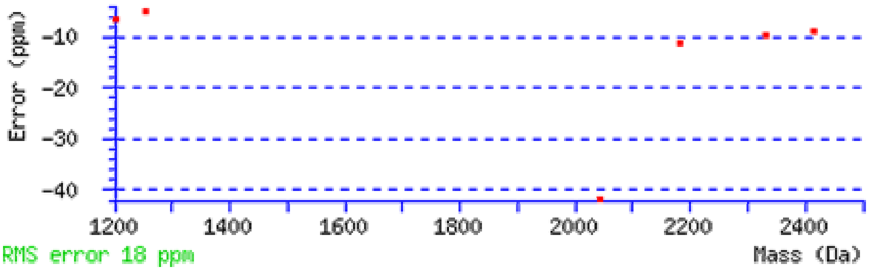

MATRIX SCIENCE MASCOT Search Results

Protein View: TRFE_HUMAN

Serotransferrin OS=Homo sapiens OX=9606 GN=TF PE=1 SV=3

**Database:**	SwissProt
**Score:**	152
**Expect:**	1.3e-11
**Monoisotopic mass (M_r):**	79294
**Calculated pI:**	6.81
**Taxonomy:**	Homo sapiens

Sequence similarity is available as an NCBI BLAST search of TRFE_HUMAN against nr.

Search parameters

**Enzyme:**	Trypsin: cuts C-term side of KR unless next residue is P.
**Fixed modifications:**	Carbamidomethyl (C)
**Variable modifications:**	Oxidation (M)
**Mass values searched:**	95
**Mass values matched:**	25

Protein sequence coverage: 33%

Matched peptides shown in ***bold red***.

```
  1 MRLAVGALLV CAVLGLCLAV PDKTVRWCAV SEHEATKCQS FRDHMKSVIP
 51 SDGPSVACVK KASYLDCIRA IAANEADAVT LDAGLVYDAY LAPNNLKPVV
101 AEFYGSKEDP QTFYYAVAVV KKDSGFQMNQ LRGKKSCHTG LGRSAGWNIP
151 IGLLYCDLPE PRKPLEKAVA NFFSGSCAPC ADGTDFPQLC QLCPGCGCST
201 LNQYFGYSGA FKCLKDGAGD VAFVKHSTIF ENLANKADRD QYELLCLDNT
251 RKPVDEYKDC HLAQVPSHTV VARSMGGKED LIWELLNQAQ EHFGKDKSKE
301 FQLFSSPHGK DLLFKDSAHG FLKVPPRMDA KMYLGYEYVT AIRNLREGTC
351 PEAPTDECKP VKWCALSHHE RLKCDEWSVN SVGKIECVSA ETTEDCIAKI
401 MNGEADAMSL DGGFVYIAGK CGLVPVLAEN YNKSDNCEDT PEAGYFAIAV
451 VKKSASDLTW DNLKGKKSCH TAVGRTAGWN IPMGLLYNKI NHCRFDEFFS
501 EGCAPGSKKD SSLCKLCMGS GLNLCEPNNK EGYYGYTGAF RCLVEKGDVA
551 FVKHQTVPQN TGGKNPDPWA KNLNEKDYEL LCLDGTRKPV EEYANCHLAR
601 APNHAVVTRK DKEACVHKIL RQQQHLFGSN VTDCSGNFCL FRSETKDLLF
651 RDDTVCLAKL HDRNTYEKYL GEEYVKAVGN LRKCSTSSLL EACTFRRP
```

Unformatted sequence string: 698 residues (for pasting into other applications).

Sort by ◉ residue number ○ increasing mass ○ decreasing mass
Show ◉ matched peptides only ○ predicted peptides also

Start – End	Observed	Mr(expt)	Mr(calc)	ppm	M	Peptide
24 – 37	1673.7763	1672.7690	1672.7991	-18.0	1	K.TVRWCAVSEHEATK.C
27 – 37	1317.5662	1316.5590	1316.5819	-17.4	0	R.WCAVSEHEATK.C
61 – 69	1125.5315	1124.5242	1124.5648	-36.1	1	K.KASYLDCIR.A
62 – 69	997.4542	996.4469	996.4698	-23.0	0	K.ASYLDCIR.A
122 – 132	1323.6261	1322.6188	1322.6401	-16.1	1	K.KDSGFQMNQLR.G
123 – 132	1211.5441	1210.5368	1210.5401	-2.70	0	K.DSGFQMNQLR.G + Oxidation (M)
144 – 162	2171.0721	2170.0649	2170.0881	-10.7	0	R.SAGWNIPIGLLYCDLPEPR.K
237 – 251	1881.9075	1880.9003	1880.8687	16.8	1	K.ADRDQYELLCLDNTR.K
240 – 251	1539.6842	1538.6769	1538.7035	-17.3	0	R.DQYELLCLDNTR.K
252 – 273	2549.2953	2548.2881	2548.2856	0.95	1	R.KPVDEYKDCHLAQVPSHTVVAR.S
298 – 310	1491.7170	1490.7097	1490.7518	-28.2	1	K.SKEFQLFSSPHGK.D
300 – 310	1276.6019	1275.5946	1275.6248	-23.7	0	K.EFQLFSSPHGK.D
328 – 343	1939.8748	1938.8675	1938.9219	-28.1	1	R.MDAKMYLGYEYVTAIR.N + Oxidation (M)
332 – 343	1478.7112	1477.7039	1477.7275	-16.0	0	K.MYLGYEYVTAIR.N
332 – 343	1494.6823	1493.6750	1493.7224	-31.7	0	K.MYLGYEYVTAIR.N + Oxidation (M)
363 – 371	1195.5207	1194.5134	1194.5352	-18.3	0	K.WCALSHHER.L
372 – 384	1521.6995	1520.6922	1520.7293	-24.4	1	R.LKCDEWSVNSVGK.I
454 – 464	1249.6074	1248.6001	1248.5986	1.22	0	K.SASDLTWDNLK.G

Start – End	Observed	Mr(expt)	Mr(calc)	ppm	M	Peptide
531 – 541	1283.5464	1282.5391	1282.5618	-17.7	0	K.EGYYGYTGAFR.C
572 – 587	1952.9306	1951.9233	1951.9309	-3.90	1	K.NLNEKDYELLCLDGTR.K
588 – 600	1586.7526	1585.7454	1585.7671	-13.7	0	R.KPVEEYANCHLAR.A
601 – 609	964.5057	963.4985	963.5250	-27.6	0	R.APNHAVVTR.K
647 – 659	1565.7719	1564.7646	1564.7919	-17.5	1	K.DLLFRDDTVCLAK.L
669 – 676	1000.4736	999.4664	999.4913	-24.9	0	K.YLGEEYVK.A
684 – 696	1531.6641	1530.6568	1530.6807	-15.6	0	K.CSTSSLLEACTFR.R

No match to: 806.3029, 833.4825, 836.3980, 864.4605, 896.4401, 900.5118, 909.4053, 949.4210, 1012.5311, 1017.5199, 1029.5627, 1033.5502, 1036.4978, 1061.5471, 1148.5492, 1162.5366, 1189.5506, 1209.5260, 1213.5953, 1220.5509, 1255.6044, 1263.6182, 1338.6423, 1350.6488, 1352.6613, 1372.6586, 1386.7772, 1398.5728, 1418.5731, 1442.4144, 1448.6320, 1464.7217, 1514.6448, 1545.6818, 1552.7351, 1561.7047, 1579.7796, 1591.7917, 1600.7638, 1616.7480, 1620.7910, 1625.7720, 1646.6860, 1660.7235, 1703.8285, 1730.8471, 1759.8666, 1773.9937, 1811.9185, 1851.9095, 1883.8668, 1895.9519, 1925.8401, 1963.8930, 1966.9351, 2001.9016, 2070.9587, 2081.0165, 2084.9664, 2110.0527, 2154.9435, 2185.0887, 2203.0696, 2212.0231, 2225.0772, 2232.2346, 2246.2482, 2265.0848, 2377.1333, 2563.3055

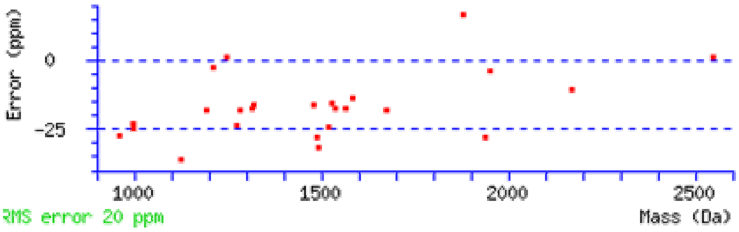

MASCOT Search Results

Protein View: A1AT_HUMAN

Alpha-1-antitrypsin OS=Homo sapiens OX=9606 GN=SERPINA1 PE=1 SV=3

**Database:**	SwissProt
**Score:**	98
**Expect:**	3.1e-06
**Monoisotopic mass (M_r):**	46878
**Calculated pI:**	5.37
**Taxonomy:**	Homo sapiens

Sequence similarity is available as an NCBI BLAST search of A1AT_HUMAN against nr.

Search parameters

**Enzyme:**	Trypsin: cuts C-term side of KR unless next residue is P.
**Fixed modifications:**	Carbamidomethyl (C)
**Variable modifications:**	Oxidation (M)
**Mass values searched:**	41
**Mass values matched:**	10

Protein sequence coverage: 27%

Matched peptides shown in ***bold red***.

```
  1 MPSSVSWGIL LLAGLCCLVP VSLAEDPQGD AAQKTDTSHH DQDHPTFNKI
 51 TPNLAEFAFS LYRQLAHQSN STNIFFSPVS IATAFAMLSL GTKADTHDEI
101 LEGLNFNLTE IPEAQIHEGF QELLRTLNQP DSQLQLTTGN GLFLSEGLKL
151 VDKFLEDVKK LYHSEAFTVN FGDTEEAKKQ INDYVEKGTQ GKIVDLVKEL
201 DRDTVFALVN YIFFKGKWER PFEVKDTEEE DFHVDQVTTV KVPMMKRLGM
251 FNIQHCKKLS SWVLLMKYLG NATAIFFLPD EGKLQHLENE LTHDIITKFL
301 ENEDRRSASL HLPKLSITGT YDLKSVLGQL GITKVFSNGA DLSGVTEEAP
351 LKLSKAVHKA VLTIDEKGTE AAGAMFLEAI PMSIPPEVKF NKPFVFLMIE
401 QNTKSPLFMG KVVNPTQK
```

Unformatted sequence string: 418 residues (for pasting into other applications).

Sort by ◉ residue number ○ increasing mass ○ decreasing mass
Show ◉ matched peptides only ○ predicted peptides also

Start – End	Observed	Mr(expt)	Mr(calc)	ppm	M	Peptide
50 – 63	1641.8278	1640.8205	1640.8562	-21.8	0	K.ITPNLAEFAFSLYR.Q
150 – 160	1333.7102	1332.7029	1332.7653	-46.8	2	K.LVDKFLEDVKK.L
161 – 179	2185.9936	2184.9864	2185.0327	-21.2	1	K.LYHSEAFTVNFGDTEEAKK.Q
193 – 215	2757.4331	2756.4259	2756.5153	-32.4	2	K.IVDLVKELDRDTVFALVNYIFFK.G
199 – 215	2090.0489	2089.0416	2089.0884	-22.4	1	K.ELDRDTVFALVNYIFFK.G
216 – 225	1275.6584	1274.6511	1274.6771	-20.4	1	K.GKWERPFEVK.D
247 – 257	1403.6731	1402.6658	1402.6962	-21.7	1	K.RLGMFNIQHCK.K
299 – 306	1078.5059	1077.4986	1077.5203	-20.1	1	K.FLENEDRR.S
315 – 324	1110.5552	1109.5479	1109.5968	-44.1	0	K.LSITGTYDLK.S
325 – 334	1015.5856	1014.5784	1014.6073	-28.6	0	K.SVLGQLGITK.V

No match to: 910.4660, 991.3701, 993.4395, 997.3814, 1019.3428, 1037.5817, 1047.3717, 1049.3650, 1057.3498, 1059.3851, 1132.5594, 1163.4403, 1166.4240, 1216.6149, 1220.4668, 1269.5595, 1307.6426, 1355.7092, 1663.7964, 1801.7064, 1825.8762, 1856.8854, 1877.9080, 1893.8750, 2112.0186, 2207.9732, 2596.2458, 2715.1913, 2731.2280, 3148.3646, 3180.3577

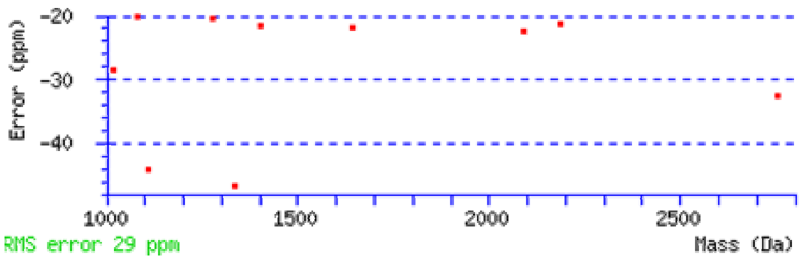
-20
-30
-40
Error (ppm)
1000
1500
2000
2500
RMS error 29 ppm
Mass (Da)

MATRIX SCIENCE MASCOT Search Results

Protein View: APOA4_HUMAN

Apolipoprotein A-IV OS=Homo sapiens OX=9606 GN=APOA4 PE=1 SV=3

**Database:**	SwissProt
**Score:**	127
**Expect:**	4.1e-09
**Monoisotopic mass (M_r):**	45371
**Calculated pI:**	5.28
**Taxonomy:**	Homo sapiens

Sequence similarity is available as an NCBI BLAST search of APOA4_HUMAN against nr.

Search parameters

**Enzyme:**	Trypsin: cuts C-term side of KR unless next residue is P.
**Fixed modifications:**	Carbamidomethyl (C)
**Variable modifications:**	Oxidation (M)
**Mass values searched:**	72
**Mass values matched:**	17

Protein sequence coverage: 37%

Matched peptides shown in ***bold red***.

```
  1 MFLKAVVLTL ALVAVAGARA EVSADQVATV MWDYFSQLSN NAKEAVEHLQ
 51 KSELTQQLNA LFQDKLGEVN TYAGDLQKKL VPFATELHER LAKDSEKLKE
101 EIGKELEELR ARLLPHANEV SQKIGDNLRE LQQRLEPYAD QLRTQVNTQA
151 EQLRRQLTPY AQRMERVLRE NADSLQASLR PHADELKAKI DQNVEELKGR
201 LTPYADEFKV KIDQTVEELR RSLAPYAQDT QEKLNHQLEG LTFQMKKNAE
251 ELKARISASA EELRQRLAPL AEDVRGNLRG NTEGLQKSLA ELGGHLDQQV
301 EEFRRRVEPY GENFNKALVQ QMEQLRQKLG PHAGDVEGHL SFLEKDLRDK
351 VNSFFSTFKE KESQDKTLSL PELEQQQEQQ QEQQQEQVQM LAPLES
```

Unformatted sequence string: 396 residues (for pasting into other applications).

Sort by (•) residue number ( ) increasing mass ( ) decreasing mass
Show (•) matched peptides only ( ) predicted peptides also

Start - End	Observed	Mr(expt)	Mr(calc)	ppm	M	Peptide
79 - 90	1439.8258	1438.8185	1438.7932	17.6	1	K.KLVPFATELHER.L
80 - 90	1311.7298	1310.7225	1310.6983	18.5	0	K.LVPFATELHER.L
124 - 134	1341.6693	1340.6620	1340.7160	-40.3	1	K.IGDNLRELQQR.L
135 - 143	1104.5936	1103.5863	1103.5611	22.8	0	R.LEPYADQLR.T
144 - 155	1443.7846	1442.7773	1442.7590	12.7	1	R.TQVNTQAEQLRR.Q
155 - 163	1132.6439	1131.6366	1131.6149	19.2	1	R.RQLTPYAQR.M
156 - 163	976.5419	975.5346	975.5138	21.4	0	R.QLTPYAQR.M
190 - 200	1300.6979	1299.6906	1299.6783	9.53	1	K.IDQNVEELKGR.L
199 - 209	1296.6480	1295.6407	1295.6510	-7.95	1	K.GRLTPYADEFK.V
201 - 209	1083.5429	1082.5356	1082.5284	6.67	0	R.LTPYADEFK.V
212 - 221	1258.6863	1257.6790	1257.6677	8.97	1	K.IDQTVEELRR.S
267 - 275	983.5670	982.5597	982.5447	15.2	0	R.LAPLAEDVR.G
288 - 304	1927.9782	1926.9709	1926.9435	14.2	0	K.SLAELGGHLDQQVEEFR.R
288 - 305	2084.0741	2083.0669	2083.0446	10.7	1	K.SLAELGGHLDQQVEEFRR.R
306 - 316	1352.6888	1351.6815	1351.6520	21.8	1	R.RVEPYGENFNK.A

Start - End	Observed	Mr(expt)	Mr(calc)	ppm	M	Peptide
317 - 326	1215.6702	1214.6629	1214.6441	15.5	0	K.ALVQQMEQLR.Q
329 - 345	1805.9297	1804.9225	1804.9108	6.47	0	K.LGPHAGDVEGHLSFLEK.D

No match to: 805.3172, 806.3257, 810.3573, 812.4041, 820.3092, 826.2984, 836.2974, 842.2797, 844.2874, 848.2890, 864.2745, 879.3837, 881.3443, 887.3934, 889.3875, 896.4195, 920.4506, 923.5308, 942.4642, 945.5263, 959.5158, 980.5180, 993.4652, 997.4929, 1012.5058, 1049.3988, 1057.3844, 1058.3915, 1125.5786, 1160.6169, 1164.5703, 1176.6023, 1192.6034, 1220.5182, 1242.4985, 1257.6756, 1283.5845, 1338.6445, 1374.6673, 1461.7965, 1478.7556, 1495.7034, 1508.7822, 1565.8029, 1585.9006, 1607.8789, 1656.8503, 1721.8523, 1742.8298, 1752.9820, 1773.9928, 1827.9204, 1834.9345, 1849.9194, 2517.1152

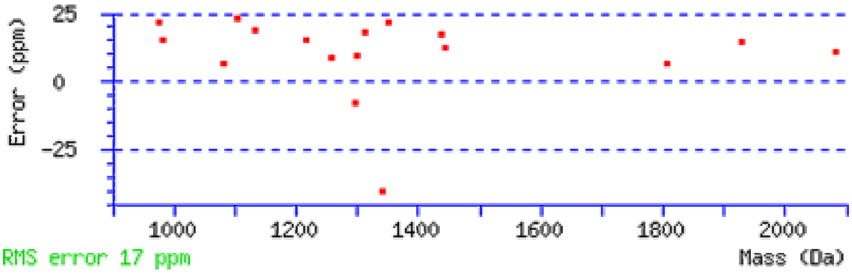

MASCOT Search Results

Protein View: HPT_HUMAN

Haptoglobin OS=Homo sapiens OX=9606 GN=HP PE=1 SV=1

**Database:**	SwissProt
**Score:**	109
**Expect:**	2.6e-07
**Monoisotopic mass (M_r):**	45861
**Calculated pI:**	6.13
**Taxonomy:**	Homo sapiens

Sequence similarity is available as an NCBI BLAST search of HPT_HUMAN against nr.

Search parameters

**Enzyme:**	Trypsin: cuts C-term side of KR unless next residue is P.
**Fixed modifications:**	Carbamidomethyl (C)
**Variable modifications:**	Oxidation (M)
**Mass values searched:**	85
**Mass values matched:**	14

Protein sequence coverage: 33%

Matched peptides shown in ***bold red***.

```
  1 MSALGAVIAL LLWGQLFAVD SGNDVTDIAD DGCPKPPEIA HGYVEHSVRY
 51 QCKNYYKLRT EGDGVYTLND KKQWINKAVG DKLPECEADD GCPKPPEIAH
101 GYVEHSVRYQ CKNYYKLRTE GDGVYTLNNE KQWINKAVGD KLPECEAVCG
151 KPKNPANPVQ RILGGHLDAK GSFPWQAKMV SHHNLTTGAT LINEQWLLTT
201 AKNLFLNHSE NATAKDIAPT LTLYVGKKQL VEIEKVVLHP NYSQVDIGLI
251 KLKQKVSVNE RVMPICLPSK DYAEVGRVGY VSGWGRNANF KFTDHLKYVM
301 LPVADQDQCI RHYEGSTVPE KKTPKSPVGV QPILNEHTFC AGMSKYQEDT
351 CYGDAGSAFA VHDLEEDTWY ATGILSFDKS CAVAEYGVYV KVTSIQDWVQ
401 KTIAEN
```

Unformatted sequence string: 406 residues (for pasting into other applications).

Sort by ◉ residue number ○ increasing mass ○ decreasing mass
Show ◉ matched peptides only ○ predicted peptides also

Start - End	Observed	Mr(expt)	Mr(calc)	ppm	M	Peptide
162 - 170	923.5485	922.5413	922.5236	19.1	0	R.ILGGHLDAK.G
171 - 178	920.4769	919.4696	919.4552	15.7	0	K.GSFPWQAK.M
216 - 227	1290.7392	1289.7319	1289.7231	6.85	0	K.DIAPTLTLYVGK.K
216 - 228	1418.8465	1417.8392	1417.8181	14.9	1	K.DIAPTLTLYVGKK.Q
229 - 235	858.4943	857.4870	857.4858	1.37	0	K.QLVEIEK.V
254 - 261	959.5478	958.5405	958.5196	21.9	1	K.QKVSVNER.V
262 - 277	1834.9734	1833.9661	1833.9117	29.6	1	R.VMPICLPSKDYAEVGR.V
271 - 277	809.4102	808.4030	808.3715	38.9	0	K.DYAEVGR.V
278 - 286	980.5336	979.5263	979.4876	39.5	0	R.VGYVSGWGR.N
298 - 311	1707.8687	1706.8614	1706.8120	29.0	0	K.YVMLPVADQDQCIR.H
312 - 322	1274.6685	1273.6612	1273.6302	24.3	1	R.HYEGSTVPEKK.T
326 - 345	2172.0981	2171.0908	2171.0504	18.6	0	K.SPVGVQPILNEHTFCAGMSK.Y
380 - 391	1345.6648	1344.6575	1344.6384	14.2	0	K.SCAVAEYGVYVK.V
392 - 401	1203.6541	1202.6469	1202.6295	14.4	0	K.VTSIQDWVQK.T

No match to: 806.3576, 820.3330, 826.3276, 828.3517, 829.3691, 831.3781, 836.3084, 842.2846, 844.3085, 848.3047, 860.3589, 864.2837, 880.4994, 887.3919, 888.3954, 889.4263, 896.4505, 899.3928, 926.5110, 942.4867, 945.5466, 958.4582, 961.5135, 974.5148, 976.5354, 994.5275, 1002.5139, 1012.5193, 1018.4898, 1057.4071, 1058.4071, 1060.4188, 1066.5704, 1069.4091, 1127.6075, 1160.6310, 1164.6120, 1168.5647, 1176.6222, 1182.6026, 1192.6205, 1215.6439, 1225.6653, 1234.7393, 1241.6298, 1296.6677, 1312.7538, 1328.7313, 1352.6991, 1367.6808, 1381.6896, 1440.8543, 1495.7257, 1532.8276, 1627.8444, 1659.8698, 1673.8749, 1721.8847, 1729.8525, 1737.8567, 1742.8559, 1745.8273, 1753.0072, 1814.9542, 1848.9763, 1856.9516, 2063.9987, 2095.9860, 2127.9858, 2194.1005, 2209.0901

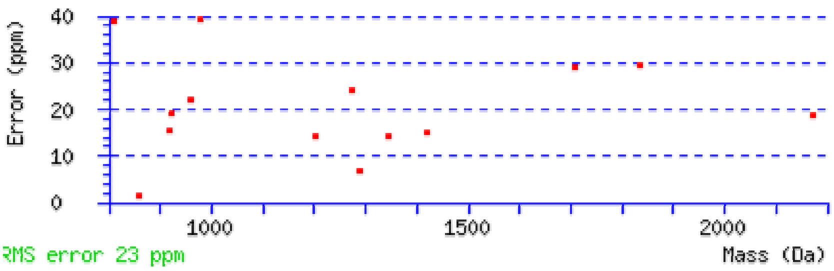

MASCOT Search Results

Protein View: HPT_HUMAN

Haptoglobin OS=Homo sapiens OX=9606 GN=HP PE=1 SV=1

**Database:**	SwissProt
**Score:**	123
**Expect:**	1e-08
**Monoisotopic mass (M_r):**	45861
**Calculated pI:**	6.13
**Taxonomy:**	Homo sapiens

Sequence similarity is available as an NCBI BLAST search of HPT_HUMAN against nr.

Search parameters

**Enzyme:**	Trypsin: cuts C-term side of KR unless next residue is P.
**Fixed modifications:**	Carbamidomethyl (C)
**Variable modifications:**	Oxidation (M)
**Mass values searched:**	29
**Mass values matched:**	13

Protein sequence coverage: 33%

Matched peptides shown in ***bold red***.

```
  1 MSALGAVIAL LLWGQLFAVD SGNDVTDIAD DGCPKPPEIA HGYVEHSVRY
 51 QCKNYYKLRT EGDGVYTLND KKQWINKAVG DKLPECEADD GCPKPPEIAH
101 GYVEHSVRYQ CKNYYKLRTE GDGVYTLNNE KQWINKAVGD KLPECEAVCG
151 KPKNPANPVQ RILGGHLDAK GSFPWQAKMV SHHNLTTGAT LINEQWLLTT
201 AKNLFLNHSE NATAKDIAPT LTLYVGKKQL VEIEKVVLHP NYSQVDIGLI
251 KLKQKVSVNE RVMPICLPSK DYAEVGRVGY VSGWGRNANF KFTDHLKYVM
301 LPVADQDQCI RHYEGSTVPE KKTPKSPVGV QPILNEHTFC AGMSKYQEDT
351 CYGDAGSAFA VHDLEEDTWY ATGILSFDKS CAVAEYGVYV KVTSIQDWVQ
401 KTIAEN
```

Unformatted sequence string: 406 residues (for pasting into other applications).

Sort by (•) residue number ( ) increasing mass ( ) decreasing mass
Show (•) matched peptides only ( ) predicted peptides also

Start – End	Observed	Mr(expt)	Mr(calc)	ppm	M	Peptide
162 – 170	923.5187	922.5114	922.5236	-13.2	0	R.ILGGHLDAK.G
171 – 178	920.4445	919.4372	919.4552	-19.5	0	K.GSFPWQAK.M
216 – 228	1418.7999	1417.7926	1417.8181	-17.9	1	K.DIAPTLTLYVGKK.Q
236 – 251	1794.9926	1793.9853	1794.0040	-10.4	0	K.VVLHPNYSQVDIGLIK.L
262 – 277	1834.9145	1833.9072	1833.9117	-2.47	1	R.VMPICLPSKDYAEVGR.V
262 – 277	1850.9047	1849.8974	1849.9066	-4.97	1	R.VMPICLPSKDYAEVGR.V + Oxidation (M)
278 – 286	980.4961	979.4888	979.4876	1.30	0	R.VGYVSGWGR.N
298 – 311	1707.8112	1706.8040	1706.8120	-4.72	0	K.YVMLPVADQDQCIR.H
298 – 311	1723.7788	1722.7715	1722.8069	-20.6	0	K.YVMLPVADQDQCIR.H + Oxidation (M)
312 – 322	1274.6241	1273.6169	1273.6302	-10.5	1	R.HYEGSTVPEKK.T
326 – 345	2172.0412	2171.0339	2171.0504	-7.59	0	K.SPVGVQPILNEHTFCAGMSK.Y
380 – 391	1345.6297	1344.6224	1344.6384	-11.9	0	K.SCAVAEYGVYVK.V
392 – 401	1203.6215	1202.6142	1202.6295	-12.8	0	K.VTSIQDWVQK.T

No match to: 1012.4791, 1160.5889, 1164.5811, 1192.5747, 1234.6895, 1495.6757, 1509.6839, 1685.7507, 1721.8256, 1737.8151, 1742.7969, 1752.9457, 1774.9204, 1816.9740, 1845.0189, 1874.8669

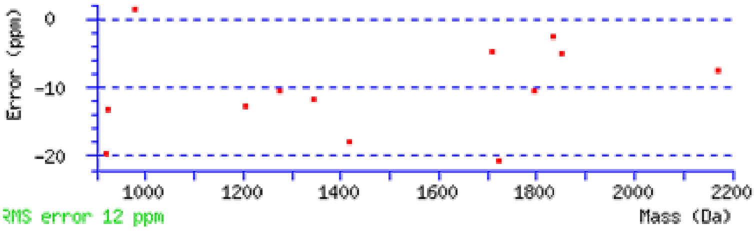
Error (ppm)
0
-10
-20
1000
1200
1400
1600
1800
2000
2200
RMS error 12 ppm
Mass (Da)

MATRIX SCIENCE MASCOT Search Results

Protein View: APOA1_HUMAN

Apolipoprotein A-I OS=Homo sapiens OX=9606 GN=APOA1 PE=1 SV=1

**Database:**	SwissProt
**Score:**	191
**Expect:**	1.6e-15
**Monoisotopic mass (M_r):**	30759
**Calculated pI:**	5.56
**Taxonomy:**	Homo sapiens

Sequence similarity is available as an NCBI BLAST search of APOA1_HUMAN against nr.

Search parameters

**Enzyme:**	Trypsin: cuts C-term side of KR unless next residue is P.
**Fixed modifications:**	Carbamidomethyl (C)
**Variable modifications:**	Oxidation (M)
**Mass values searched:**	98
**Mass values matched:**	23

Protein sequence coverage: 71%

Matched peptides shown in ***bold red***.

```
  1 MKAAVLTLAV LFLTGSQARH FWQQDEPPQS PWDRVKDLAT VYVDVLKDSG
 51 RDYVSQFEGS ALGKQLNLKL LDNWDSVTST FSKLREQLGP VTQEFWDNLE
101 KETEGLRQEM SKDLEEVKAK VQPYLDDFQK KWQEEMELYR QKVEPLRAEL
151 QEGARQKLHE LQEKLSPLGE EMRDRARAHV DALRTHLAPY SDELRQRLAA
201 RLEALKENGG ARLAEYHAKA TEHLSTLSEK AKPALEDLRQ GLLPVLESFK
251 VSFLSALEEY TKKLNTQ
```

Unformatted sequence string: 267 residues (for pasting into other applications).

Sort by ◉ residue number ○ increasing mass ○ decreasing mass
Show ◉ matched peptides only ○ predicted peptides also

Start – End	Observed	Mr(expt)	Mr(calc)	ppm	M	Peptide
35 – 47	1462.8248	1461.8175	1461.8443	-18.3	1	R.VKDLATVYVDVLK.D
37 – 47	1235.6406	1234.6334	1234.6809	-38.5	0	K.DLATVYVDVLK.D
48 – 64	1815.8664	1814.8591	1814.8435	8.60	1	K.DSGRDYVSQFEGSALGK.Q
52 – 64	1400.6506	1399.6433	1399.6620	-13.3	0	R.DYVSQFEGSALGK.Q
70 – 83	1612.7816	1611.7743	1611.7781	-2.31	0	K.LLDNWDSVTSTFSK.L
84 – 101	2202.1243	2201.1170	2201.1117	2.42	1	K.LREQLGPVTQEFWDNLEK.E
102 – 112	1323.6061	1322.5988	1322.6136	-11.2	1	K.ETEGLRQEMSK.D + Oxidation (M)
121 – 130	1252.6167	1251.6094	1251.6136	-3.28	0	K.VQPYLDDFQK.K
121 – 131	1380.7031	1379.6959	1379.7085	-9.16	1	K.VQPYLDDFQKK.W
131 – 140	1411.6716	1410.6643	1410.6601	2.93	1	K.KWQEEMELYR.Q
132 – 140	1283.5738	1282.5665	1282.5652	1.05	0	K.WQEEMELYR.Q
143 – 155	1467.7810	1466.7737	1466.7841	-7.06	1	K.VEPLRAELQEGAR.Q
148 – 155	873.4464	872.4392	872.4352	4.59	0	R.AELQEGAR.Q
165 – 173	1047.4918	1046.4845	1046.5066	-21.1	0	K.LSPLGEEMR.D + Oxidation (M)
165 – 175	1318.6222	1317.6149	1317.6347	-15.0	1	K.LSPLGEEMRDR.A + Oxidation (M)
185 – 195	1301.6477	1300.6404	1300.6411	-0.57	0	R.THLAPYSDELR.Q
185 – 197	1585.8026	1584.7954	1584.8008	-3.45	1	R.THLAPYSDELRQR.L
202 – 212	1157.6162	1156.6089	1156.6200	-9.56	1	R.LEALKENGGAR.L
213 – 219	831.4185	830.4112	830.4286	-21.0	0	R.LAEYHAK.A
220 – 230	1215.6289	1214.6216	1214.6143	6.04	0	K.ATEHLSTLSEK.A
231 – 239	1012.5782	1011.5709	1011.5713	-0.31	0	K.AKPALEDLR.Q
240 – 250	1230.6805	1229.6732	1229.7020	-23.4	0	R.QGLLPVLESFK.V
251 – 262	1386.6931	1385.6858	1385.7078	-15.9	0	K.VSFLSALEEYTK.K

No match to: 806.3176, 820.3073, 826.2799, 842.2619, 855.5099, 859.3318, 861.3599, 869.3898, 879.3716, 887.3625, 889.3674, 979.5530, 993.5614, 1007.5780, 1057.3568, 1059.3701, 1158.5947, 1219.6572, 1226.5387, 1266.5546, 1268.6157, 1315.5574, 1405.6679, 1408.6805, 1484.8199, 1495.6729, 1580.8064, 1602.7801, 1632.7889, 1645.7979, 1653.7629, 1661.9146, 1675.9292, 1689.8772, 1705.8905, 1723.9390, 1733.9008, 1743.8551, 1765.8481, 1775.8486, 1797.9000, 1811.9147, 1819.9022, 1870.9907, 1878.9977, 1884.0078, 1946.0107, 1968.0051, 2037.9130, 2051.9329, 2065.0389, 2084.8869, 2094.9423, 2102.1329, 2108.9815, 2137.1176, 2141.0338, 2169.1222, 2206.1028, 2234.1261, 2303.1680, 2309.0439, 2323.0749, 2352.1872, 2380.2003, 2409.2121, 2423.2270, 2439.2305, 2450.2495, 2453.2422, 2466.2508, 2481.2564, 2494.2630, 2624.3350, 2677.3036

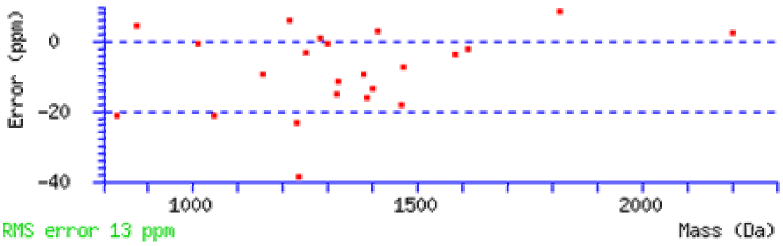

MATRIX SCIENCE MASCOT Search Results

Protein View: RET4_HUMAN

Retinol-binding protein 4 OS=Homo sapiens OX=9606 GN=RBP4 PE=1 SV=3

**Database:**	SwissProt
**Score:**	70
**Expect:**	0.0019
**Monoisotopic mass (M_r):**	23337
**Calculated pI:**	5.76
**Taxonomy:**	Homo sapiens

Sequence similarity is available as an NCBI BLAST search of RET4_HUMAN against nr.

Search parameters

**Enzyme:**	Trypsin: cuts C-term side of KR unless next residue is P.
**Fixed modifications:**	Carbamidomethyl (C)
**Variable modifications:**	Oxidation (M)
**Mass values searched:**	13
**Mass values matched:**	5

Protein sequence coverage: 33%

Matched peptides shown in ***bold red***.

1 MKWVWALLLL AALGSGRAER DCRVSSFRVK **ENFDKAR**FSG TWYAMAKKDP
51 EGLFLQDNIV AEFSVDETGQ MSATAKGRVR LLNNWDVCAD MVGTFTDTED
101 PAKFKMKYWG VASFLQK**GND** **DHWIVDTDYD** **TYAVQYSCRL** **LNLDGTCADS**
151 **YSFVFSR**DPN GLPPEAQKIV R**QRQEELCLA** **R**QYR**LIVHNG** **YCDGR**SERNL
201 L

Unformatted sequence string: 201 residues (for pasting into other applications).

Sort by ◉ residue number ○ increasing mass ○ decreasing mass
Show ◉ matched peptides only ○ predicted peptides also

Start – End	Observed	Mr(expt)	Mr(calc)	ppm	M	Peptide
31 – 37	879.4060	878.3987	878.4246	-29.5	1	K.ENFDKAR.F
118 – 139	2693.1786	2692.1713	2692.1136	21.4	0	K.GNDDHWIVDTDYDTYAVQYSCR.L
140 – 157	2064.9919	2063.9847	2063.9623	10.9	0	R.LLNLDGTCADSYSFVFSR.D
172 – 181	1302.6585	1301.6513	1301.6510	0.21	1	R.QRQEELCLAR.Q
185 – 195	1303.6413	1302.6341	1302.6139	15.5	0	R.LIVHNGYCDGR.S

No match to: 803.3802, 887.3827, 895.3929, 2676.1381, 2707.1841, 2715.1574, 2725.1726, 2845.1762

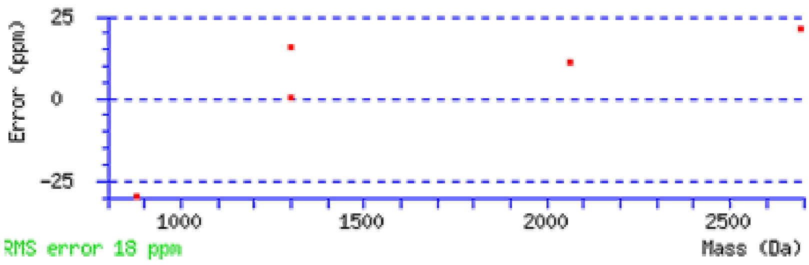

MATRIX SCIENCE MASCOT Search Results

Protein View: HPT_HUMAN

Haptoglobin OS=Homo sapiens OX=9606 GN=HP PE=1 SV=1

**Database:**	SwissProt
**Score:**	62
**Expect:**	0.011
**Monoisotopic mass (M_r):**	45861
**Calculated pI:**	6.13
**Taxonomy:**	Homo sapiens

Sequence similarity is available as an NCBI BLAST search of HPT_HUMAN against nr.

Search parameters

**Enzyme:**	Trypsin: cuts C-term side of KR unless next residue is P.
**Fixed modifications:**	Carbamidomethyl (C)
**Variable modifications:**	Oxidation (M)
**Mass values searched:**	52
**Mass values matched:**	8

Protein sequence coverage: 21%

Matched peptides shown in ***bold red***.

```
  1 MSALGAVIAL LLWGQLFAVD SGNDVTDIAD DGCPKPPEIA HGYVEHSVRY
 51 QCKNYYKLRT EGDGVYTLND KKQWINKAVG DKLPECEADD GCPKPPEIAH
101 GYVEHSVRYQ CKNYYKLRTE GDGVYTLNNE KQWINKAVGD KLPECEAVCG
151 KPKNPANPVQ RILGGHLDAK GSFPWQAKMV SHHNLTTGAT LINEQWLLTT
201 AKNLFLNHSE NATAKDIAPT LTLYVGKKQL VEIEKVVLHP NYSQVDIGLI
251 KLKQKVSVNE RVMPICLPSK DYAEVGRVGY VSGWGRNANF KFTDHLKYVM
301 LPVADQDQCI RHYEGSTVPE KKTPKSPVGV QPILNEHTFC AGMSKYQEDT
351 CYGDAGSAFA VHDLEEDTWY ATGILSFDKS CAVAEYGVYV KVTSIQDWVQ
401 KTIAEN
```

Unformatted sequence string: 406 residues (for pasting into other applications).

Sort by ◉ residue number ○ increasing mass ○ decreasing mass
Show ◉ matched peptides only ○ predicted peptides also

Start – End	Observed	Mr(expt)	Mr(calc)	ppm	M	Peptide
58 – 71	1580.7821	1579.7748	1579.7842	-5.96	1	K.LRTEGDGVYTLNDK.K
78 – 108	3432.6397	3431.6325	3431.5874	13.1	1	K.AVGDKLPECEADDGCPKPPEIAHGYVEHSVR.Y
83 – 108	2962.3669	2961.3596	2961.3385	7.12	0	K.LPECEADDGCPKPPEIAHGYVEHSVR.Y
117 – 131	1708.8695	1707.8622	1707.8428	11.4	1	K.LRTEGDGVYTLNNEK.Q
119 – 131	1439.6740	1438.6667	1438.6576	6.35	0	R.TEGDGVYTLNNEK.Q
119 – 136	2109.0464	2108.0391	2108.0174	10.3	1	R.TEGDGVYTLNNEKQWINK.A
154 – 161	895.4694	894.4621	894.4671	-5.66	0	K.NPANPVQR.I
298 – 311	1723.8001	1722.7928	1722.8069	-8.21	0	K.YVMLPVADQDQCIR.H + Oxidation (M)

No match to: 897.3565, 899.3691, 901.3695, 903.3802, 993.4297, 1057.3665, 1058.3675, 1060.3780, 1069.3631, 1106.5854, 1161.5031, 1163.4464, 1198.6184, 1220.4959, 1230.5978, 1285.6265, 1303.6282, 1304.5689, 1317.6233, 1347.5372, 1505.7258, 1675.7664, 1682.7634, 1717.2898, 1730.8297, 2064.9828, 2078.9959, 2378.2329, 2410.2200, 2636.1222, 2649.1358, 2677.1207, 2679.8777, 2693.1589, 2707.1545, 2716.1171, 2725.1448, 2739.1459, 2976.3878, 3349.5677, 3363.5906, 3417.5049, 3446.6574, 3460.6543

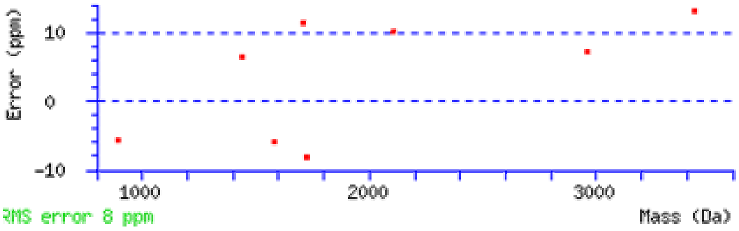

Error (ppm)
10
0
-10
1000
2000
3000
RMS error 8 ppm
Mass (Da)

MATRIX SCIENCE MASCOT Search Results

Protein View: HPT_HUMAN

Haptoglobin OS=Homo sapiens OX=9606 GN=HP PE=1 SV=1

**Database:**	SwissProt
**Score:**	60
**Expect:**	0.022
**Monoisotopic mass (M_r):**	45861
**Calculated pI:**	6.13
**Taxonomy:**	Homo sapiens

Sequence similarity is available as an NCBI BLAST search of HPT_HUMAN against nr.

Search parameters

**Enzyme:**	Trypsin: cuts C-term side of KR unless next residue is P.
**Fixed modifications:**	Carbamidomethyl (C)
**Variable modifications:**	Oxidation (M)
**Mass values searched:**	38
**Mass values matched:**	6

Protein sequence coverage: 16%

Matched peptides shown in ***bold red***.

1 MSALGAVIAL LLWGQLFAVD SGNDVTDIAD DGCPKPPEIA HGYVEHSVRY
51 QCKNYYK**LRT EGDGVYTLND** K**K**QWINK**AVG DKLPECEADD GCPKPPEIAH**
101 **GYVEHSVR**YQ CKNYYK**LRTE GDGVYTLNNE K**QWINKAVGD KLPECEAVCG
151 KPKNPANPVQ RILGGHLDAK GSFPWQAKMV SHHNLTTGAT LINEQWLLTT
201 AKNLFLNHSE NATAKDIAPT LTLYVGKKQL VEIEKVVLHP NYSQVDIGLI
251 KLKQKVSVNE RVMPICLPSK **DYAEVGR**VGY VSGWGRNANF KFTDHLKYVM
301 LPVADQDQCI RHYEGSTVPE KKTPKSPVGV QPILNEHTFC AGMSKYQEDT
351 CYGDAGSAFA VHDLEEDTWY ATGILSFDKS CAVAEYGVYV KVTSIQDWVQ
401 KTIAEN

Unformatted sequence string: 406 residues (for pasting into other applications).

Sort by ◉ residue number ○ increasing mass ○ decreasing mass
Show ◉ matched peptides only ○ predicted peptides also

Start – End	Observed	Mr(expt)	Mr(calc)	ppm	M	Peptide
58 – 71	1580.7589	1579.7516	1579.7842	-20.6	1	K.LRTEGDGVYTLNDK.K
60 – 71	1311.5977	1310.5905	1310.5990	-6.54	0	R.TEGDGVYTLNDK.K
78 – 108	3432.6286	3431.6213	3431.5874	9.88	1	K.AVGDKLPECEADDGCPKPPEIAHGYVEHSVR.Y
83 – 108	2962.3461	2961.3389	2961.3385	0.11	0	K.LPECEADDGCPKPPEIAHGYVEHSVR.Y
117 – 131	1708.8414	1707.8342	1707.8428	-5.03	1	K.LRTEGDGVYTLNNEK.Q
271 – 277	809.3566	808.3493	808.3715	-27.5	0	K.DYAEVGR.V

No match to: 800.3451, 801.3705, 803.3641, 805.3823, 807.3957, 819.3379, 825.3708, 830.3505, 845.3537, 852.3576, 879.3788, 887.3705, 889.3759, 895.3855, 899.3863, 919.4034, 1057.3584, 1058.3607, 1267.6405, 1289.6242, 1684.8375, 1730.8151, 1964.9382, 2378.1887, 2394.1799, 2415.1472, 2976.3607, 3349.5558, 3361.0096, 3363.5637, 3403.5543, 3446.6305

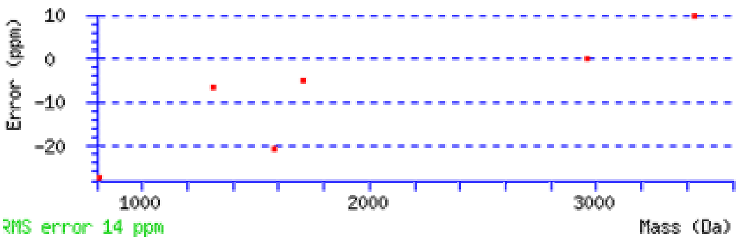

MATRIX SCIENCE MASCOT Search Results

Protein View: TTHY_HUMAN

Transthyretin OS=Homo sapiens OX=9606 GN=TTR PE=1 SV=1

**Database:**	SwissProt
**Score:**	64
**Expect:**	0.0081
**Monoisotopic mass (M_r):**	15991
**Calculated pI:**	5.52
**Taxonomy:**	Homo sapiens

Sequence similarity is available as an NCBI BLAST search of TTHY_HUMAN against nr.

Search parameters

**Enzyme:**	Trypsin: cuts C-term side of KR unless next residue is P.
**Fixed modifications:**	Carbamidomethyl (C)
**Variable modifications:**	Oxidation (M)
**Mass values searched:**	65
**Mass values matched:**	6

Protein sequence coverage: 61%

Matched peptides shown in ***bold red***.

```
  1 MASHRLLLLC LAGLVFVSEA GPTGTGESKC PLMVKVLDAV RGSPAINVAV
 51 HVFRKAADDT WEPFASGKTS ESGELHGLTT EEEFVEGIYK VEIDTKSYWK
101 ALGISPFHEH AEVVFTANDS GPRRYTIAAL LSPYSYSTTA VVTNPKE
```

Unformatted sequence string: 147 residues (for pasting into other applications).

Sort by ⦿ residue number ○ increasing mass ○ decreasing mass
Show ⦿ matched peptides only ○ predicted peptides also

Start – End	Observed	Mr(expt)	Mr(calc)	ppm	M	Peptide
30 – 41	1416.6379	1415.6306	1415.7629	-93.4	1	K.CPLMVKVLDAVR.G + Oxidation (M)
42 – 54	1366.7816	1365.7743	1365.7517	16.5	0	R.GSPAINVAVHVFR.K
55 – 68	1522.7470	1521.7397	1521.7100	19.6	1	R.KAADDTWEPFASGK.T
56 – 68	1394.6468	1393.6395	1393.6150	17.6	0	K.AADDTWEPFASGK.T
69 – 96	3140.5632	3139.5559	3139.5085	15.1	1	K.TSESGELHGLTTEEEFVEGIYKVEIDTK.S
101 – 123	2451.2658	2450.2586	2450.1979	24.8	0	K.ALGISPFHEHAEVVFTANDSGPR.R

No match to: 820.3859, 822.3767, 824.3827, 829.4568, 833.3989, 838.4381, 846.3893, 856.5263, 864.4590, 870.5501, 895.4615, 897.4285, 908.4467, 929.4930, 934.4477, 951.4799, 962.4752, 982.4537, 993.5146, 1002.5493, 1016.5464, 1033.5290, 1037.5285, 1046.5618, 1060.5584, 1065.5226, 1068.5239, 1107.5627, 1118.5271, 1121.5817, 1131.6564, 1157.5929, 1189.6149, 1194.6238, 1229.6773, 1234.6904, 1265.6520, 1284.5922, 1307.7057, 1311.6276, 1317.6457, 1357.7376, 1411.7805, 1432.6300, 1434.7149, 1439.7327, 1450.7778, 1464.7760, 1537.7588, 1580.8267, 1608.7958, 1612.8750, 1708.9214, 1751.8887, 1791.7799, 1794.8374, 1851.9701, 1857.9608, 2065.9970

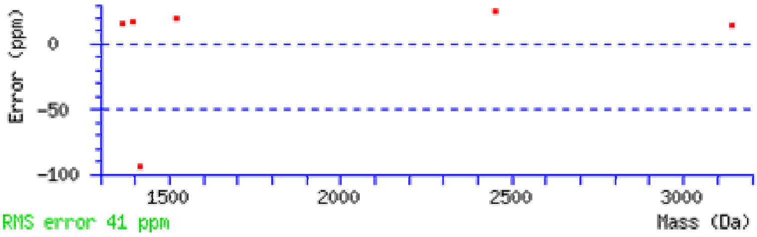

RMS error 41 ppm

MASCOT Search Results

Protein View: HPT_HUMAN

Haptoglobin OS=Homo sapiens GN=HP PE=1 SV=1

**Database:**	SwissProt
**Score:**	64
**Expect:**	0.008
**Nominal mass (M_r):**	45861
**Calculated pI:**	6.13
**Taxonomy:**	Homo sapiens

Sequence similarity is available as an NCBI BLAST search of HPT_HUMAN against nr.

Search parameters

**Enzyme:**	Trypsin: cuts C-term side of KR unless next residue is P.
**Fixed modifications:**	Carbamidomethyl (C)
**Variable modifications:**	Oxidation (M)
**Mass values searched:**	81
**Mass values matched:**	10

Protein sequence coverage: 19%

Matched peptides shown in ***bold red***.

```
  1 MSALGAVIAL LLWGQLFAVD SGNDVTDIAD DGCPKPPEIA HGYVEHSVRY
 51 QCKNYYKLRT EGDGVYTLND KKQWINKAVG DKLPECEADD GCPKPPEIAH
101 GYVEHSVRYQ CKNYYKLRTE GDGVYTLNNE KQWINKAVGD KLPECEAVCG
151 KPKNPANPVQ RILGGHLDAK GSFPWQAKMV SHHNLTTGAT LINEQWLLTT
201 AKNLFLNHSE NATAKDIAPT LTLYVGKKQL VEIEKVVLHP NYSQVDIGLI
251 KLKQKVSVNE RVMPICLPSK DYAEVGRVGY VSGWGRNANF KFTDHLKYVM
301 LPVADQDQCI RHYEGSTVPE KKTPKSPVGV QPILNEHTFC AGMSKYQEDT
351 CYGDAGSAFA VHDLEEDTWY ATGILSFDKS CAVAEYGVYV KVTSIQDWVQ
401 KTIAEN
```

Unformatted sequence string: 406 residues (for pasting into other applications).

Sort peptides by ◉ Residue Number ○ Increasing Mass ○ Decreasing Mass

Show predicted peptides also

Start – End	Observed	Mr(expt)	Mr(calc)	ppm	M	Peptide
58 – 71	1580.8454	1579.8381	1579.7842	34.1	1	K.LRTEGDGVYTLNDK.K
60 – 71	1311.6498	1310.6425	1310.5990	33.2	0	R.TEGDGVYTLNDK.K
60 – 72	1439.7408	1438.7335	1438.6940	27.5	1	R.TEGDGVYTLNDKK.Q
113 – 118	856.5123	855.5050	855.4603	52.3	1	K.NYYKLR.T
117 – 131	1708.9585	1707.9512	1707.8428	63.5	1	K.LRTEGDGVYTLNNEK.Q
137 – 153	1857.9926	1856.9853	1856.9124	39.2	1	K.AVGDKLPECEAVCGKPK.N
142 – 153	1387.7088	1386.7015	1386.6635	27.4	0	K.LPECEAVCGKPK.N
154 – 161	895.5063	894.4990	894.4671	35.7	0	K.NPANPVQR.I
228 – 235	986.5370	985.5298	985.5808	-51.8	1	K.KQLVEIEK.V
312 – 322	1274.7130	1273.7058	1273.6302	59.3	1	R.HYEGSTVPEKK.T

No match to: 820.3984, 822.4516, 824.4226, 830.4521, 833.4226, 836.4647, 838.4726, 839.3575, 848.4429, 869.3826, 870.5281, 873.4573, 880.4565, 881.4837, 887.4361, 899.4657, 913.5113, 917.4760, 921.5032, 927.5120, 929.5450, 945.4888, 946.5040, 964.5340, 968.5782, 998.5432, 1033.5704, 1108.5862, 1117.5409, 1132.5793, 1138.5461, 1149.6068, 1152.5638, 1205.5870, 1286.7040, 1287.6882, 1313.7879, 1314.7267, 1318.6225, 1331.7396, 1333.6300, 1366.7941, 1379.2050, 1394.6691, 1461.7405, 1497.8438, 1522.7508, 1529.7794, 1545.7805, 1604.9875, 1693.8878, 1797.9501, 1812.9856, 1848.9073, 1872.0110, 1880.0068, 1894.9975, 1911.0051, 2018.5006, 2262.1784, 2276.0221, 2355.5774, 2369.8066, 2451.3325, 2516.5968, 2615.8135, 2630.0413, 3174.5680, 3334.7752, 3349.7638, 3363.7276

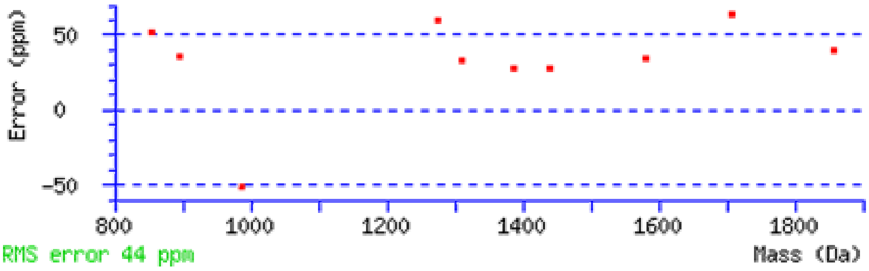

MATRIX SCIENCE MASCOT Search Results

Protein View: SAA1_HUMAN

Serum amyloid A-1 protein OS=Homo sapiens OX=9606 GN=SAA1 PE=1 SV=1

**Database:**	SwissProt
**Score:**	63
**Expect:**	0.01
**Monoisotopic mass (M_r):**	13581
**Calculated pI:**	6.28
**Taxonomy:**	Homo sapiens

Sequence similarity is available as an NCBI BLAST search of SAA1_HUMAN against nr.

Search parameters

**Enzyme:**	Trypsin: cuts C-term side of KR unless next residue is P.
**Fixed modifications:**	Carbamidomethyl (C)
**Variable modifications:**	Oxidation (M)
**Mass values searched:**	39
**Mass values matched:**	5

Protein sequence coverage: 55%

Matched peptides shown in ***bold red***.

```
  1 MKLLTGLVFC SLVLGVSSRS FFSFLGEAFD GARDMWRAYS DMREANYIGS
 51 DKYFHARGNY DAAKRGPGGV WAAEAISDAR ENIQRFFGHG AEDSLADQAA
101 NEWGRSGKDP NHFRPAGLPE KY
```

Unformatted sequence string: 122 residues (for pasting into other applications).

Sort by (•) residue number ( ) increasing mass ( ) decreasing mass
Show (•) matched peptides only ( ) predicted peptides also

Start - End	Observed	Mr(expt)	Mr(calc)	ppm	M	Peptide
20 - 33	1550.7146	1549.7073	1549.7202	-8.28	0	R.SFFSFLGEAFDGAR.D
20 - 37	2138.9551	2137.9478	2137.9680	-9.45	1	R.SFFSFLGEAFDGARDMWR.A
44 - 57	1670.7946	1669.7874	1669.7848	1.52	1	R.EANYIGSDKYFHAR.G
65 - 80	1612.7949	1611.7876	1611.8117	-14.9	1	K.RGPGGVWAAEAISDAR.E
86 - 105	2177.9472	2176.9400	2176.9562	-7.48	0	R.FFGHGAEDSLADQAANEWGR.S

No match to: 915.4331, 919.4010, 1057.3753, 1274.7195, 1290.6976, 1400.6719, 1601.5489, 1639.8174, 1684.8545, 1706.8060, 1791.7161, 1848.8244, 1864.8034, 1892.9673, 1897.8046, 1912.9490, 1934.9070, 1940.9421, 2090.9553, 2102.2195, 2104.9846, 2116.2476, 2153.9570, 2155.9506, 2168.9625, 2185.9487, 2193.9496, 2200.9420, 2209.9841, 2318.9566, 2342.9688, 2705.1554, 2717.1025, 3312.2954

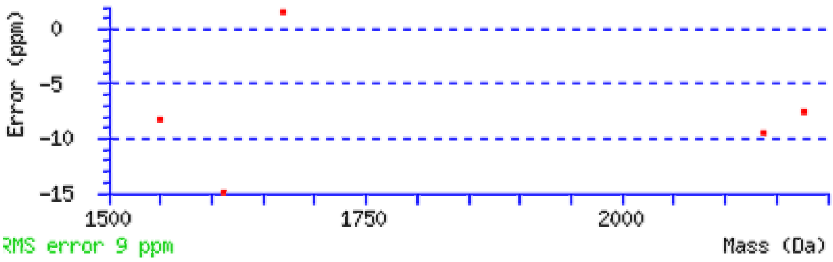

17. MALDI LIF-TOF/TOF MS spectra and Mascot searches

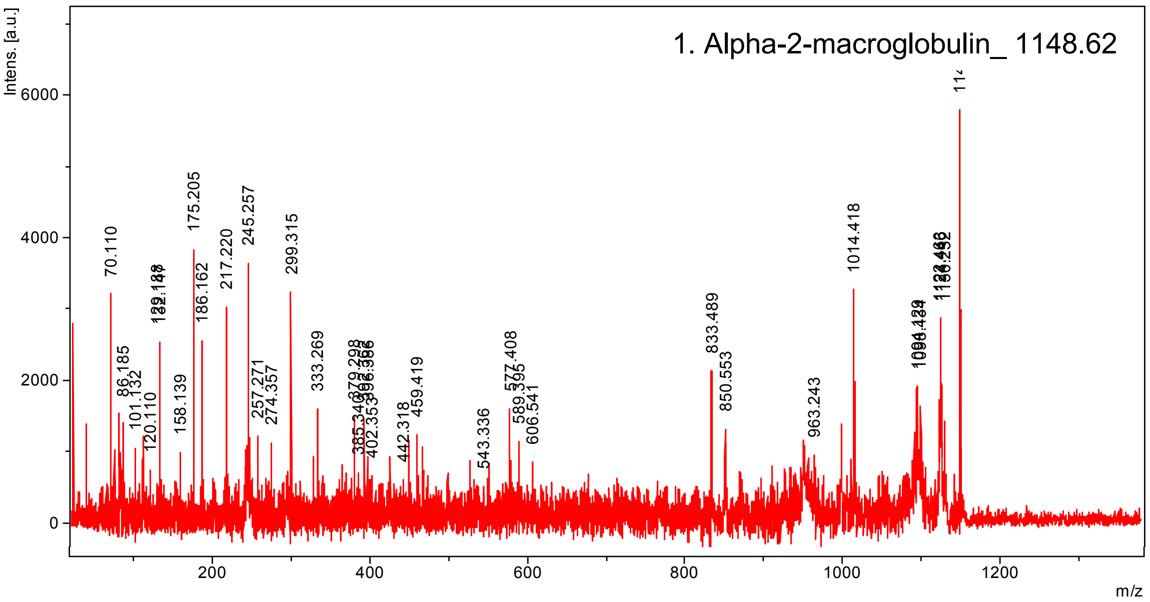

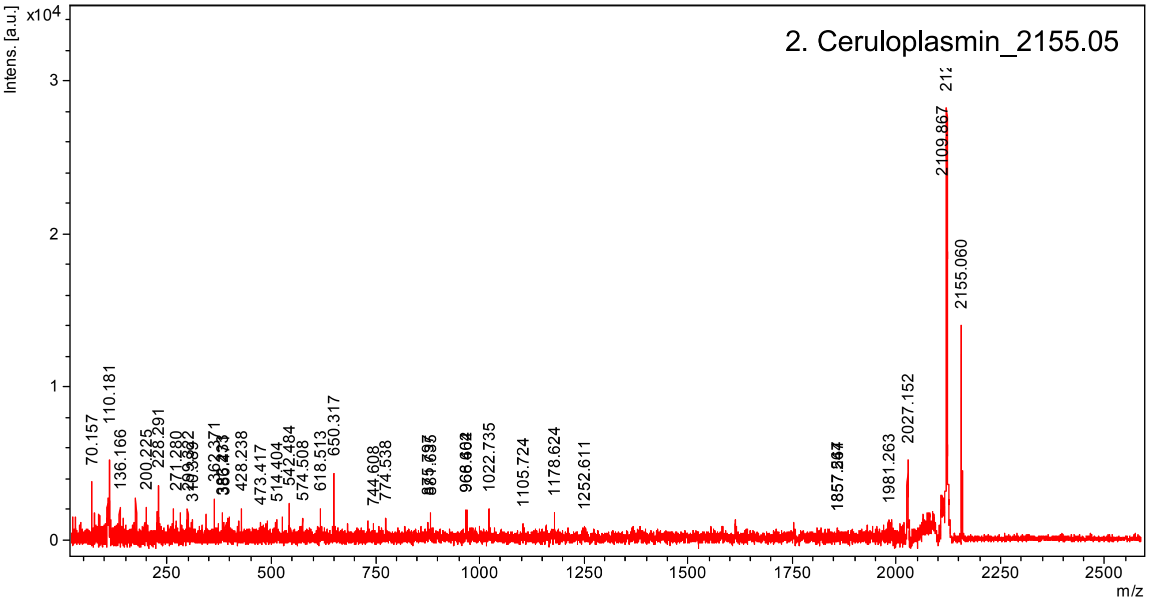

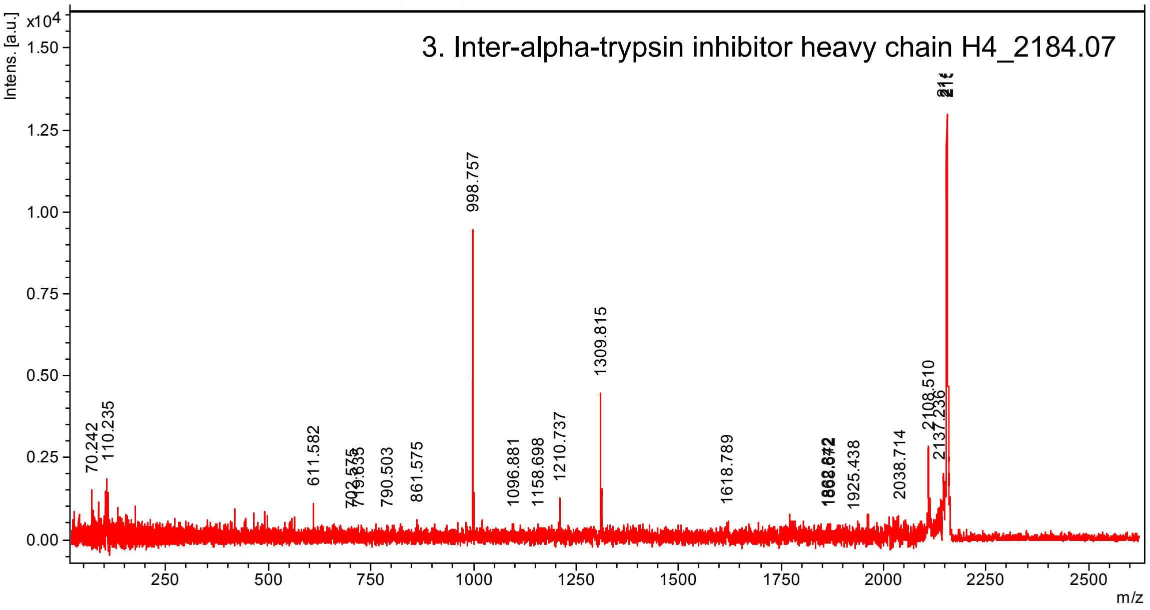

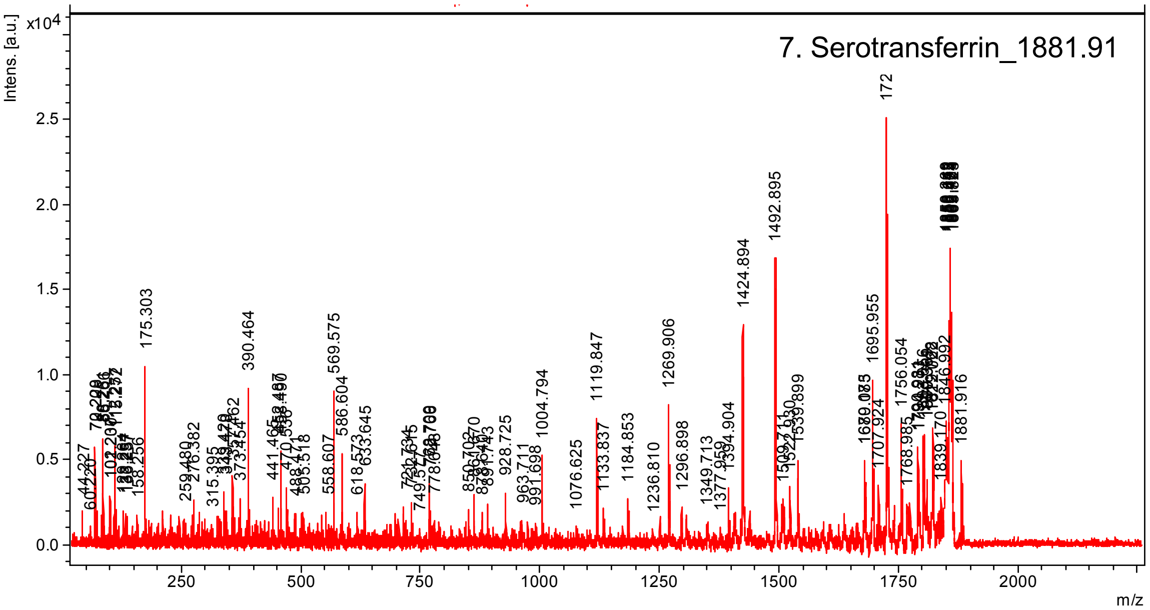
7. Serotransferrin_1881.91
Intens. [a.u.]
x10^4
m/z
175.303
390.464
569.575
1004.794
1119.847
1269.906
1424.894
1492.895
1695.955
1756.054
1881.916

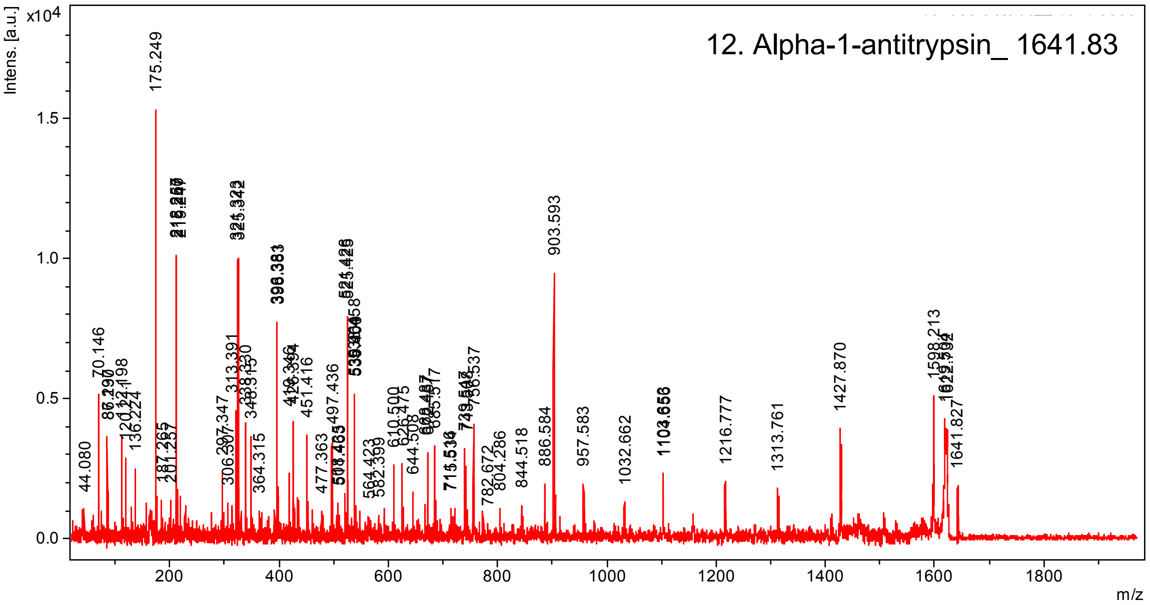
12. Alpha-1-antitrypsin_ 1641.83
Intens. [a.u.]
x10^4
m/z
175.249
903.593
1216.777
1313.761
1427.870
1598.213
1641.827

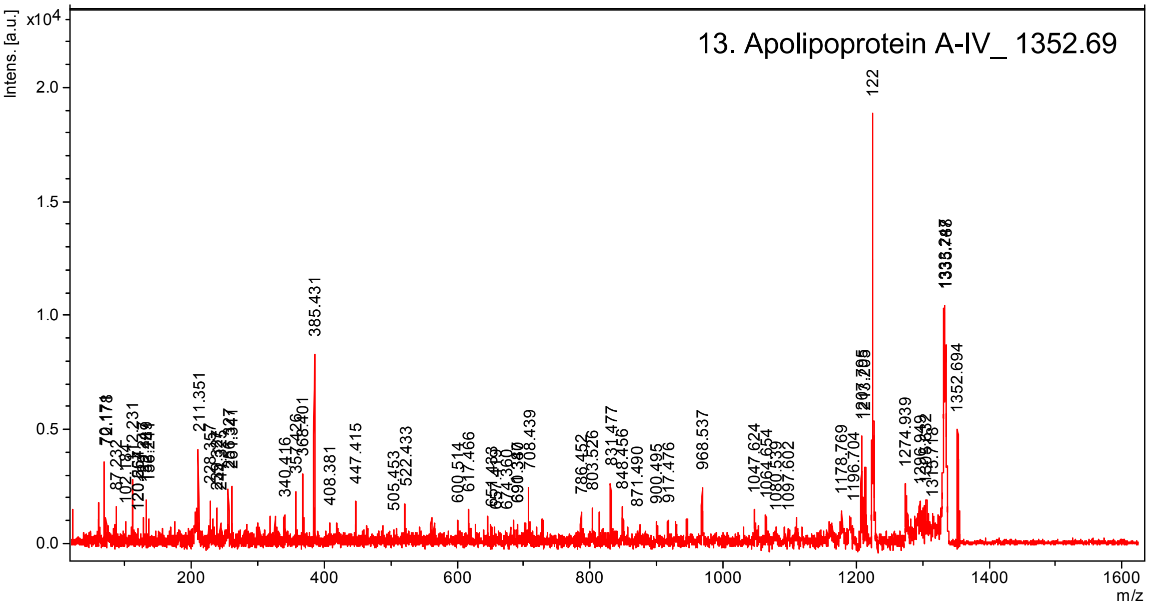
13. Apolipoprotein A-IV_ 1352.69
Intens. [a.u.]
x10^4
m/z
385.431
211.351
968.537
1352.694

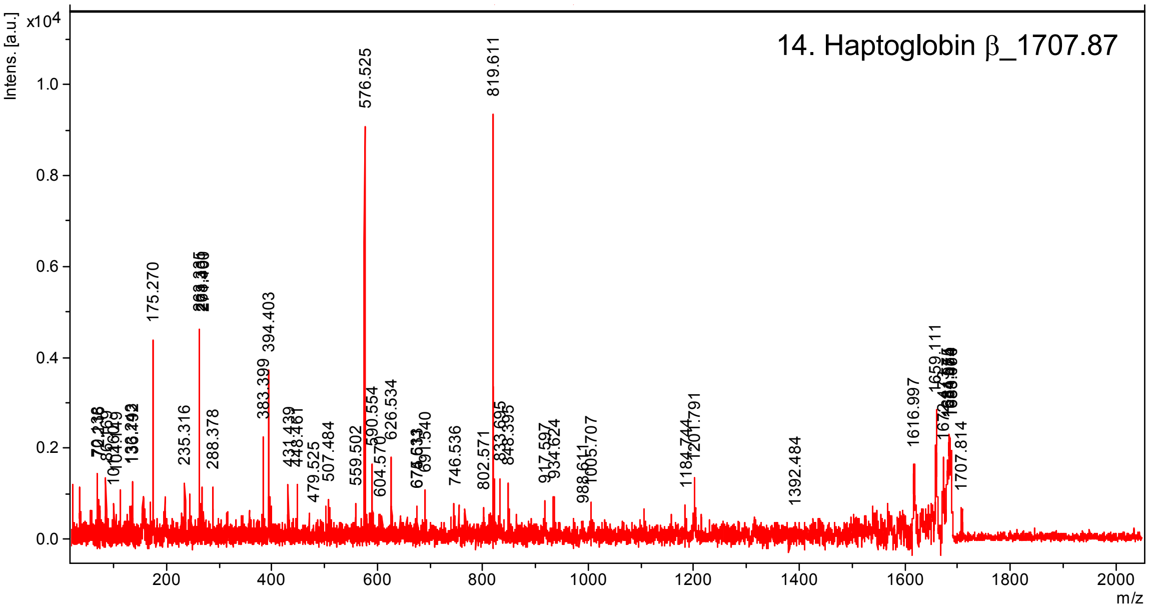
14. Haptoglobin β_1707.87
Intens. [a.u.]
x10^4
m/z
576.525
819.611
175.270
394.403
383.399
1616.997
1707.814
1392.484
1201.791

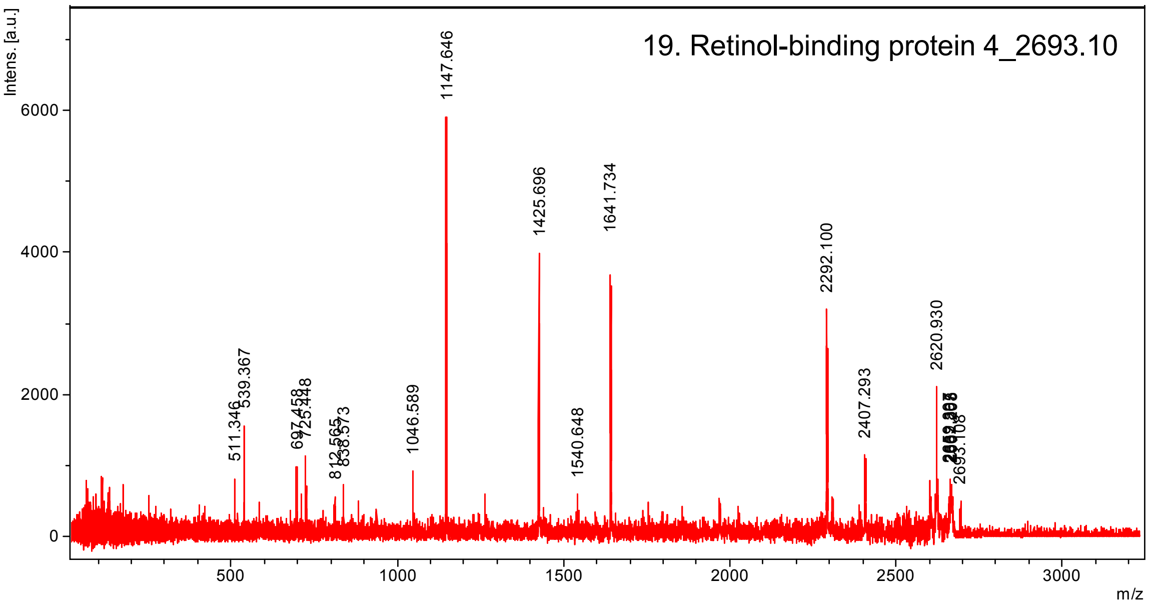
19. Retinol-binding protein 4_2693.10
Intens. [a.u.]
m/z
1147.646
1425.696
1641.734
2292.100
2620.930
2407.293
1540.648
1046.589
539.367
511.346
2693.108

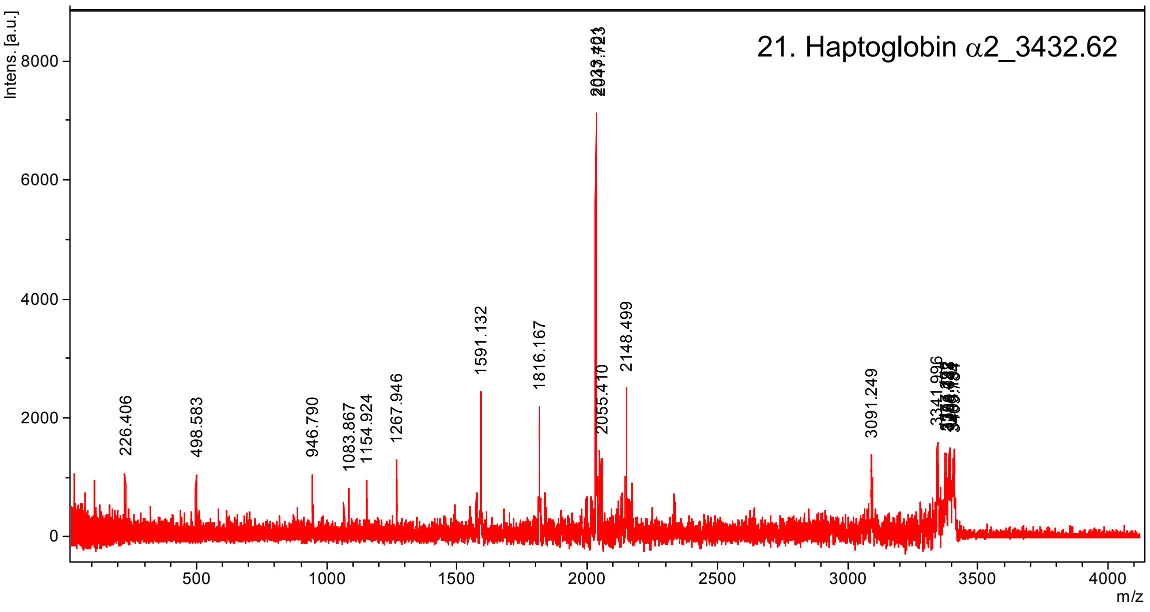
21. Haptoglobin α2_3432.62
Intens. [a.u.]
m/z
2148.499
2055.410
1816.167
1591.132
1267.946
1154.924
1083.867
946.790
498.583
226.406
3091.249

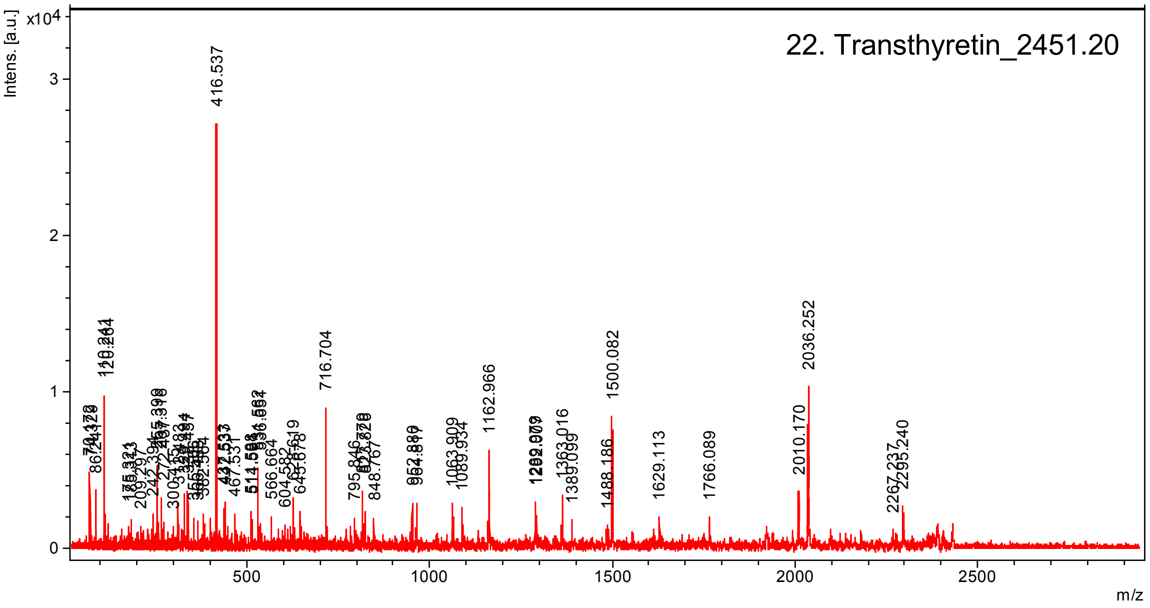
22. Transthyretin_2451.20
Intens. [a.u.]
x10^4
m/z
416.537
716.704
1162.966
1500.082
2036.252
2010.170
1629.113
1766.089
1363.016
2295.240

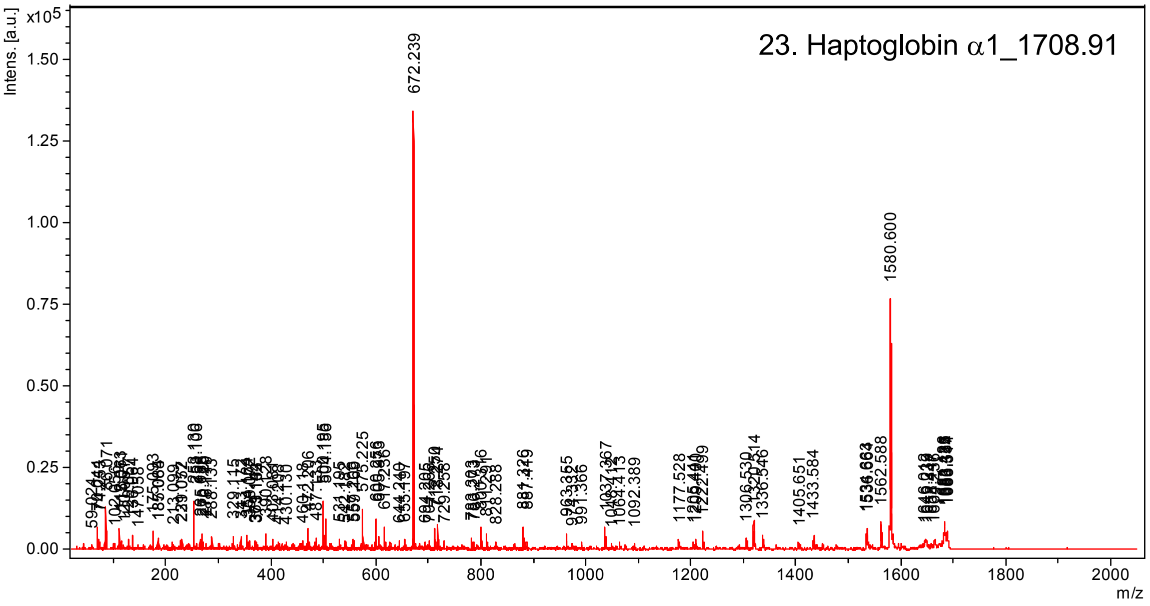
23. Haptoglobin α1_1708.91
Intens. [a.u.]
x10^5
m/z
672.239
1580.600
1562.588

MATRIX SCIENCE MASCOT Search Results

Protein View: A2MG_HUMAN

Alpha-2-macroglobulin OS=Homo sapiens OX=9606 GN=A2M PE=1 SV=3

**Database:**	SwissProt
**Score:**	63
**Monoisotopic mass (M_r):**	164613
**Calculated pI:**	6.03
**Taxonomy:**	Homo sapiens

Sequence similarity is available as an NCBI BLAST search of A2MG_HUMAN against nr.

Search parameters

**MS data file:**	DATA.TXT
**Enzyme:**	Trypsin: cuts C-term side of KR unless next residue is P.
**Fixed modifications:**	Carbamidomethyl (C)
**Variable modifications:**	Oxidation (M)

Protein sequence coverage: 0%

Matched peptides shown in ***bold red***.

**1**	MGKNKLLHPS	LVLLLLVLLP	TDASVSGKPQ	YMVLVPSLLH	TETTEKGCVL
**51**	LSYLNETVTV	SASLESVRGN	RSLFTDLEAE	NDVLHCVAFA	VPKSSSNEEV
**101**	MFLTVQVKGP	TQEFKKRTTV	MVKNEDSLVF	VQTDKSIYKP	GQTVKFRVVS
**151**	MDENFHPLNE	LIPLVYIQDP	KGNRIAQWQS	FQLEGGLKQF	SFPLSSEPFQ
**201**	GSYKVVVQKK	SGGRTEHPFT	VEEFVLPKFE	VQVTVPKIIT	ILEEEMNVSV
**251**	CGLYTYGKPV	PGHVTVSICR	KYSDASDCHG	EDSQAFCEKF	SGQLNSHGCF
**301**	YQQVKTKVFQ	LKRKEYEMKL	HTEAQIQEEG	TVVELTGRQS	SEITRTITKL
**351**	SFVKVDSHFR	**QGIPFFGQVR**	LVDGKGVPIP	NKVIFIRGNE	ANYYSNATTD
**401**	EHGLVQFSIN	TTNVMGTSLT	VRVNYKDRSP	CYGYQWVSEE	HEEAHHTAYL
**451**	VFSPSKSFVH	LEPMSHELPC	GHTQTVQAHY	ILNGGTLLGL	KKLSFYYLIM
**501**	AKGGIVRTGT	HGLLVKQEDM	KGHFSISIPV	KSDIAPVARL	LIYAVLPTGD
**551**	VIGDSAKYDV	ENCLANKVDL	SFSPSQSLPA	SHAHLRVTAA	PQSVCALRAV
**601**	DQSVLLMKPD	AELSASSVYN	LLPEKDLTGF	PGPLNDQDNE	DCINRHNVYI
**651**	NGITYTPVSS	TNEKDMYSFL	EDMGLKAFTN	SKIRKPKMCP	QLQQYEMHGP
**701**	EGLRVGFYES	DVMGRGHARL	VHVEEPHTET	VRKYFPETWI	WDLVVVNSAG
**751**	VAEVGVTVPD	TITEWKAGAF	CLSEDAGLGI	SSTASLRAFQ	PFFVELTMPY
**801**	SVIRGEAFTL	KATVLNYLPK	CIRVSVQLEA	SPAFLAVPVE	KEQAPHCICA
**851**	NGRQTVSWAV	TPKSLGNVNF	TVSAEALESQ	ELCGTEVPSV	PEHGRKDTVI
**901**	KPLLVEPEGL	EKETTFNSLL	CPSGGEVSEE	LSLKLPPNVV	EESARASVSV
**951**	LGDILGSAMQ	NTQNLLQMPY	GCGEQNMVLF	APNIYVLDYL	NETQQLTPEI
**1001**	KSKAIGYLNT	GYQRQLNYKH	YDGSYSTFGE	RYGRNQGNTW	LTAFVLKTFA
**1051**	QARAYIFIDE	AHITQALIWL	SQRQKDNGCF	RSSGSLLNNA	IKGGVEDEVT
**1101**	LSAYITIALL	EIPLTVTHPV	VRNALFCLES	AWKTAQEGDH	GSHVYTKALL
**1151**	AYAFALAGNQ	DKRKEVLKSL	NEEAVKKDNS	VHWERPQKPK	APVGHFYEPQ
**1201**	APSAEVEMTS	YVLLAYLTAQ	PAPTSEDLTS	ATNIVKWITK	QQNAQGGFSS
**1251**	TQDTVVALHA	LSKYGAATFT	RTGKAAQVTI	QSSGTFSSKF	QVDNNNRLLL

1301 QQVSLPELPG EYSMKVTGEG CVYLQTSLKY NILPEKEEFP FALGVQTLPQ
1351 TCDEPKAHTS FQISLSVSYT GSRSASNMAI VDVKMVSGFI PLKPTVKMLE
1401 RSNHVSRTEV SSNHVLIYLD KVSNQTLSLF FTVLQDVPVR DLKPAIVKVY
1451 DYYETDEFAI AEYNAPCSKD LGNA

Unformatted sequence string: **1474 residues** (for pasting into other applications).

Sort by ◉ residue number ○ increasing mass ○ decreasing mass
Show ◉ matched peptides only ○ predicted peptides also

Query	Start - End	Observed	Mr(expt)	Mr(calc)	ppm	M	Score	Expe
1	361 - 370	1148.6210	1147.6137	1147.6138	-0.11	0	63	1.8e-

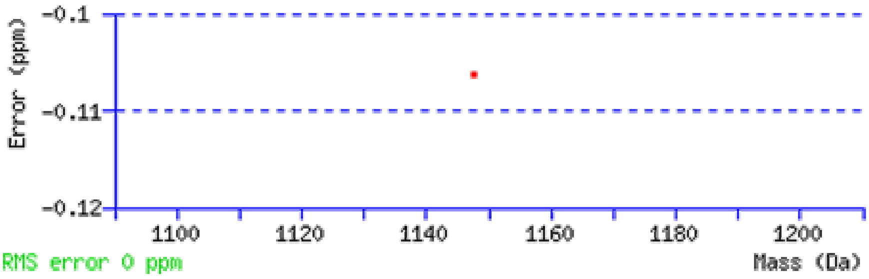

I

MATRIX SCIENCE MASCOT Search Results

Protein View: CERU_HUMAN

Ceruloplasmin OS=Homo sapiens OX=9606 GN=CP PE=1 SV=1

**Database:**	SwissProt
**Score:**	80
**Monoisotopic mass (M_r):**	122983
**Calculated pI:**	5.44
**Taxonomy:**	Homo sapiens

Sequence similarity is available as an NCBI BLAST search of CERU_HUMAN against nr.

Search parameters

**MS data file:**	DATA.TXT
**Enzyme:**	Trypsin: cuts C-term side of KR unless next residue is P.
**Fixed modifications:**	Carbamidomethyl (C)
**Variable modifications:**	Oxidation (M)

Protein sequence coverage: 1%

Matched peptides shown in ***bold red***.

```
   1 MKILILGIFL FLCSTPAWAK EKHYYIGIIE TTWDYASDHG EKKLISVDTE
  51 HSNIYLQNGP DRIGRLYKKA LYLQYTDETF RTTIEKPVWL GFLGPIIKAE
 101 TGDKVYVHLK NLASRPYTFH SHGITYYKEH EGAIYPDNTT DFQRADDKVY
 151 PGEQYTYMLL ATEEQSPGEG DGNCVTRIYH SHIDAPKDIA SGLIGPLIIC
 201 KKDSLDKEKE KHIDREFVVM FSVVDENFSW YLEDNIKTYC SEPEKVDKDN
 251 EDFQESNRMY SVNGYTFGSL PGLSMCAEDR VKWYLFGMGN EVDVHAAFFH
 301 GQALTNKNYR IDTINLFPAT LFDAYMVAQN PGEWMLSCQN LNHLKAGLQA
 351 FFQVQECNKS SSKDNIRGKH VRHYYIAAEE IIWNYAPSGI DIFTKENLTA
 401 PGSDSAVFFE QGTTRIGGSY KKLVYREYTD ASFTNRKERG PEEEHLGILG
 451 PVIWAEVGDT IRVTFHNKGA YPLSIEPIGV RFNKNNEGTY YSPNYNPQSR
 501 SVPPSASHVA PTETFTYEWT VPKEVGPTNA DPVCLAKMYY SAVDPTKDIF
 551 TGLIGPMKIC KKGSLHANGR QKDVDKEFYL FPTVFDENES LLLEDNIRMF
 601 TTAPDQVDKE DEDFQESNKM HSMNGFMYGN QPGLTMCKGD SVVWYLFSAG
 651 NEADVHGIYF SGNTYLWRGE RRDTANLFPQ TSLTLHMWPD TEGTFNVECL
 701 TTDHYTGGMK QKYTVNQCRR QSEDSTFYLG ERTYYIAAVE VEWDYSPQRE
 751 WEKELHHLQE QNVSNAFLDK GEFYIGSKYK KVVYRQYTDS TFRVPVERKA
 801 EEEHLGILGP QLHADVGDKV KIIFKNMATR PYSIHAHGVQ TESSTVTPTL
 851 PGETLTYVWK IPERSGAGTE DSACIPWAYY STVDQVKDLY SGLIGPLIVC
 901 RRPYLKVFNP RRKLEFALLF LVFDENESWY LDDNIKTYSD HPEKVNKDDE
 951 EFIESNKMHA INGRMFGNLQ GLTMHVGDEV NWYLMGMGNE IDLHTVHFHG
1001 HSFQYKHRGV YSSDVFDIFP GTYQTLEMFP RTPGIWLLHC HVTDHIHAGM
1051 ETTYTVLQNE DTKSG
```

Unformatted sequence string: 1065 residues (for pasting into other applications).

Sort by ◉ residue number ○ increasing mass ○ decreasing mass
Show ◉ matched peptides only ○ predicted peptides also

Query	Start - End	Observed	Mr(expt)	Mr(calc)	ppm	M	Score	Expe
1	111 - 128	2155.0510	2154.0437	2154.0647	-9.72	0	80	2.3e-

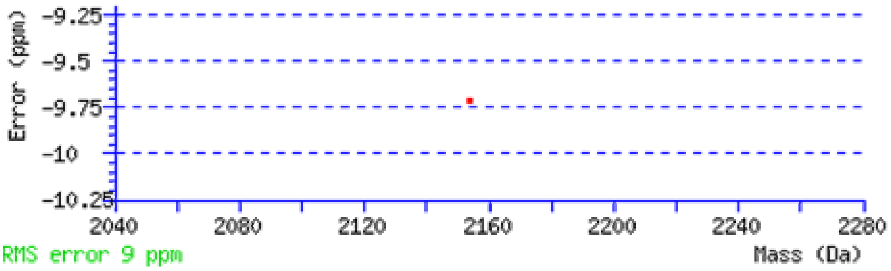

MATRIX SCIENCE MASCOT Search Results

Protein View: ITIH4_HUMAN

Inter-alpha-trypsin inhibitor heavy chain H4 OS=Homo sapiens OX=9606 GN=ITIH4 PE=1 SV=4

**Database:**	SwissProt
**Score:**	50
**Monoisotopic mass (M_r):**	103521
**Calculated pI:**	6.51
**Taxonomy:**	Homo sapiens

Sequence similarity is available as an NCBI BLAST search of ITIH4_HUMAN against nr.

Search parameters

**MS data file:**	DATA.TXT
**Enzyme:**	Trypsin: cuts C-term side of KR unless next residue is P.
**Fixed modifications:**	Carbamidomethyl (C)
**Variable modifications:**	Oxidation (M)

Protein sequence coverage: 2%

Matched peptides shown in ***bold red***.

```
  1 MKPPRPVRTC SKVLVLLSLL AIHQTTTAEK NGIDIYSLTV DSRVSSRFAH
 51 TVVTSRVVNR ANTVQEATFQ MELPKKAFIT NFSMIIDGMT YPGIIKEKAE
101 AQAQYSAAVA KGKSAGLVKA TGRNMEQFQV SVSVAPNAKI TFELVYEELL
151 KRRLGVYELL LKVRPQQLVK HLQMDIHIFE PQGISFLETE STFMTNQLVD
201 ALTTWQNKTK AHIRFKPTLS QQQKSPEQQE TVLDGNLIIR YDVDRAISGG
251 SIQIENGYFV HYFAPEGLTT MPKNVVFVID KSGSMSGRKI QQTREALIKI
301 LDDLSPRDQF NLIVFSTEAT QWRPSLVPAS AENVNKARSF AAGIQALGGT
351 NINDAMLMAV QLLDSSNQEE RLPEGSVSLI ILLTDGDPTV GETNPRSIQN
401 NVREAVSGRY SLFCLGFGFD VSYAFLEKLA LDNGGLARRI HEDSDSALQL
451 QDFYQEVANP LLTAVTFEYP SNAVEEVTQN NFRLLFKGSE MVVAGKLQDR
501 GPDVLTATVS GKLPTQNITF QTESSVAEQE AEFQSPKYIF HNFMERLWAY
551 LTIQQLLEQT VSASDADQQA LRNQALNLSL AYSFVTPLTS MVVTKPDDQE
601 QSQVAEKPME GESRNRNVHS GSTFFKYYLQ GAKIPKPEAS FSPRRGWNRQ
651 AGAAGSRMNF RPGVLSSRQL GLPGPPDVPD HAAYHPFRRL AILPASAPPA
701 TSNPDPAVSR VMNMKIEETT MTTQTPAPIQ APSAILPLPG QSVERLCVDP
751 RHRQGPVNLL SDPEQGVEVT GQYEREKAGF SWIEVTFKNP LVWVHASPEH
801 VVVTRNRRSS AYKWKETLFS VMPGLKMTMD KTGLLLLSDP DKVTIGLLFW
851 DGRGEGLRLL LRDTDRFSSH VGGTLGQFYQ EVLWGSPAAS DDGRRTLRVQ
901 GNDHSATRER RLDYQEGPPG VEISCWSVEL
```

Unformatted sequence string: 930 residues (for pasting into other applications).

Sort by	◉	residue number	○ increasing mass	○	decreasing mass
Show	◉	matched peptides only	○ predicted peptides also		

Query	Start – End	Observed	Mr(expt)	Mr(calc)	ppm	M	Score	Expect	Rank	U	Peptide
1	669 – 688	2184.0740	2183.0667	2183.0912	-11.2	0	50	0.00025	1	U	R.QLGLPGPPDVPDHAAYHPFR.R

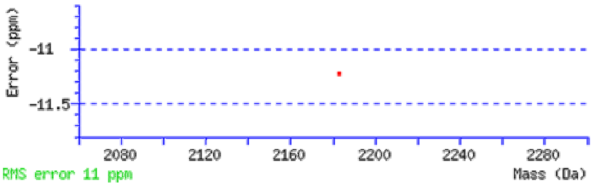

MATRIX SCIENCE MASCOT Search Results

Protein View: TRFE_HUMAN

Serotransferrin OS=Homo sapiens OX=9606 GN=TF PE=1 SV=3

**Database:**	SwissProt
**Score:**	91
**Monoisotopic mass (M_r):**	79294
**Calculated pI:**	6.81
**Taxonomy:**	Homo sapiens

Sequence similarity is available as an NCBI BLAST search of TRFE_HUMAN against nr.

Search parameters

**MS data file:**	DATA.TXT
**Enzyme:**	Trypsin: cuts C-term side of KR unless next residue is P.
**Fixed modifications:**	Carbamidomethyl (C)
**Variable modifications:**	Oxidation (M)

Protein sequence coverage: 2%

Matched peptides shown in ***bold red***.

1 MRLAVGALLV CAVLGLCLAV PDKTVRWCAV SEHEATKCQS FRDHMKSVIP
51 SDGPSVACVK KASYLDCIRA IAANEADAVT LDAGLVYDAY LAPNNLKPVV
101 AEFYGSKEDP QTFYYAVAVV KKDSGFQMNQ LRGKKSCHTG LGRSAGWNIP
151 IGLLYCDLPE PRKPLEKAVA NFFSGSCAPC ADGTDFPQLC QLCPGCGCST
201 LNQYFGYSGA FKCLKDGAGD VAFVKHSTIF ENLANK**ADRD** **QYELLCLDNT**
251 **R**KPVDEYKDC HLAQVPSHTV VARSMGGKED LIWELLNQAQ EHFGKDKSKE
301 FQLFSSPHGK DLLFKDSAHG FLKVPPRMDA KMYLGYEYVT AIRNLREGTC
351 PEAPTDECKP VKWCALSHHE RLKCDEWSVN SVGKIECVSA ETTEDCIAKI
401 MNGEADAMSL DGGFVYIAGK CGLVPVLAEN YNKSDNCEDT PEAGYFAIAV
451 VKKSASDLTW DNLKGKKSCH TAVGRTAGWN IPMGLLYNKI NHCRFDEFFS
501 EGCAPGSKKD SSLCKLCMGS GLNLCEPNNK EGYYGYTGAF RCLVEKGDVA
551 FVKHQTVPQN TGGKNPDPWA KNLNEKDYEL LCLDGTRKPV EEYANCHLAR
601 APNHAVVTRK DKEACVHKIL RQQQHLFGSN VTDCSGNFCL FRSETKDLLF
651 RDDTVCLAKL HDRNTYEKYL GEEYVKAVGN LRKCSTSSLL EACTFRRP

Unformatted sequence string: 698 residues (for pasting into other applications).

Sort by ◉ residue number ○ increasing mass ○ decreasing mass
Show ◉ matched peptides only ○ predicted peptides also

Query	Start – End	Observed	Mr(expt)	Mr(calc)	ppm	M	Score	Expect	Rank	U	Peptide
1	237 – 251	1881.9080	1880.9007	1880.8687	17.1	1	91	2e-08	1	U	K.ADRDQYELLCLDNTR.K

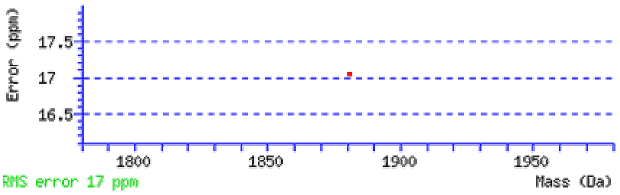

MATRIX SCIENCE MASCOT Search Results

Protein View: A1AT_HUMAN

Alpha-1-antitrypsin OS=Homo sapiens OX=9606 GN=SERPINA1 PE=1 SV=3

**Database:**	SwissProt
**Score:**	99
**Monoisotopic mass (M_r):**	46878
**Calculated pI:**	5.37
**Taxonomy:**	Homo sapiens

Sequence similarity is available as an NCBI BLAST search of A1AT_HUMAN against nr.

Search parameters

**MS data file:**	DATA.TXT
**Enzyme:**	Trypsin: cuts C-term side of KR unless next residue is P.
**Fixed modifications:**	Carbamidomethyl (C)
**Variable modifications:**	Oxidation (M)

Protein sequence coverage: 3%

Matched peptides shown in ***bold red***.

```
  1 MPSSVSWGIL LLAGLCCLVP VSLAEDPQGD AAQKTDTSHH DQDHPTFNKI
 51 TPNLAEFAFS LYRQLAHQSN STNIFFSPVS IATAFAMLSL GTKADTHDEI
101 LEGLNFNLTE IPEAQIHEGF QELLRTLNQP DSQLQLTTGN GLFLSEGLKL
151 VDKFLEDVKK LYHSEAFTVN FGDTEEAKKQ INDYVEKGTQ GKIVDLVKEL
201 DRDTVFALVN YIFFKGKWER PFEVKDTEEE DFHVDQVTTV KVPMMKRLGM
251 FNIQHCKKLS SWVLLMKYLG NATAIFFLPD EGKLQHLENE LTHDIITKFL
301 ENEDRRSASL HLPKLSITGT YDLKSVLGQL GITKVFSNGA DLSGVTEEAP
351 LKLSKAVHKA VLTIDEKGTE AAGAMFLEAI PMSIPPEVKF NKPFVFLMIE
401 QNTKSPLFMG KVVNPTQK
```

Unformatted sequence string: 418 residues (for pasting into other applications).

Sort by ◉ residue number ○ increasing mass ○ decreasing mass
Show ◉ matched peptides only ○ predicted peptides also

Query	Start - End	Observed	Mr(expt)	Mr(calc)	ppm	M	Score	Expect	Rank	U	Peptide
1	50 - 63	1641.8280	1640.8207	1640.8562	-21.6	0	99	3.8e-09	1	U	K.ITPNLAEFAFSLYR.Q

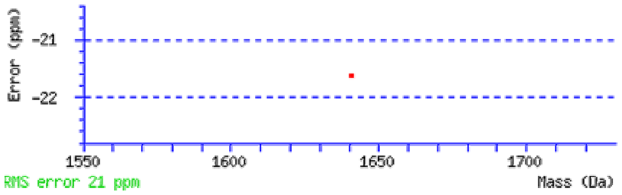

MATRIX SCIENCE MASCOT Search Results

Protein View: APOA4_HUMAN

Apolipoprotein A-IV OS=Homo sapiens OX=9606 GN=APOA4 PE=1 SV=3

**Database:**	SwissProt
**Score:**	76
**Monoisotopic mass (M_r):**	45371
**Calculated pI:**	5.28
**Taxonomy:**	Homo sapiens

Sequence similarity is available as an NCBI BLAST search of APOA4_HUMAN against nr.

Search parameters

**MS data file:**	DATA.TXT
**Enzyme:**	Trypsin: cuts C-term side of KR unless next residue is P.
**Fixed modifications:**	Carbamidomethyl (C)
**Variable modifications:**	Oxidation (M)

Protein sequence coverage: 2%

Matched peptides shown in ***bold red***.

```
  1 MFLKAVVLTL ALVAVAGARA EVSADQVATV MWDYFSQLSN NAKEAVEHLQ
 51 KSELTQQLNA LFQDKLGEVN TYAGDLQKKL VPFATELHER LAKDSEKLKE
101 EIGKELEELR ARLLPHANEV SQKIGDNLRE LQQRLEPYAD QLRTQVNTQA
151 EQLRRQLTPY AQRMERVLRE NADSLQASLR PHADELKAKI DQNVEELKGR
201 LTPYADEFKV KIDQTVEELR RSLAPYAQDT QEKLNHQLEG LTFQMKKNAE
251 ELKARISASA EELRQRLAPL AEDVRGNLRG NTEGLQKSLA ELGGHLDQQV
301 EEFRRRVEPY GENFNKALVQ QMEQLRQKLG PHAGDVEGHL SFLEKDLRDK
351 VNSFFSTFKE KESQDKTLSL PELEQQQEQQ QEQQQEQVQM LAPLES
```

Unformatted sequence string: 396 residues (for pasting into other applications).

Sort by ◉ residue number ○ increasing mass ○ decreasing mass
Show ◉ matched peptides only ○ predicted peptides also

Query	Start – End	Observed	Mr(expt)	Mr(calc)	ppm	M	Score	Expect	Rank	U	Peptide
1	306 – 316	1352.6890	1351.6817	1351.6520	22.0	1	76	7.9e-07	1	U	R.RVEPYGENFNK.A

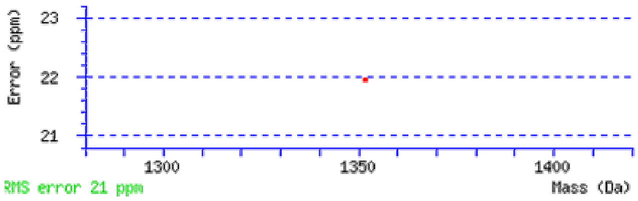

MATRIX SCIENCE MASCOT Search Results

Protein View: HPT_HUMAN

Haptoglobin OS=Homo sapiens OX=9606 GN=HP PE=1 SV=1

**Database:**	SwissProt
**Score:**	75
**Monoisotopic mass (M_r):**	45861
**Calculated pI:**	6.13
**Taxonomy:**	Homo sapiens

Sequence similarity is available as an NCBI BLAST search of HPT_HUMAN against nr.

Search parameters

**MS data file:**	DATA.TXT
**Enzyme:**	Trypsin: cuts C-term side of KR unless next residue is P.
**Fixed modifications:**	Carbamidomethyl (C)
**Variable modifications:**	Oxidation (M)

Protein sequence coverage: 3%

Matched peptides shown in ***bold red***.

```
  1 MSALGAVIAL LLWGQLFAVD SGNDVTDIAD DGCPKPPEIA HGYVEHSVRY
 51 QCKNYYKLRT EGDGVYTLND KKQWINKAVG DKLPECEADD GCPKPPEIAH
101 GYVEHSVRYQ CKNYYKLRTE GDGVYTLNNE KQWINKAVGD KLPECEAVCG
151 KPKNPANPVQ RILGGHLDAK GSFPWQAKMV SHHNLTTGAT LINEQWLLTT
201 AKNLFLNHSE NATAKDIAPT LTLYVGKKQL VEIEKVVLHP NYSQVDIGLI
251 KLKQKVSVNE RVMPICLPSK DYAEVGRVGY VSGWGRNANF KFTDHLKYVM
301 LPVADQDQCI RHYEGSTVPE KKTPKSPVGV QPILNEHTFC AGMSKYQEDT
351 CYGDAGSAFA VHDLEEDTWY ATGILSFDKS CAVAEYGVYV KVTSIQDWVQ
401 KTIAEN
```

Unformatted sequence string: 406 residues (for pasting into other applications).

Sort by ◉ residue number ○ increasing mass ○ decreasing mass
Show ◉ matched peptides only ○ predicted peptides also

Query	Start - End	Observed	Mr(expt)	Mr(calc)	ppm	M	Score	Expect	Rank	U	Peptide
1	298 - 311	1707.8690	1706.8617	1706.8120	29.1	0	75	9.6e-07	1	U	K.YVMLPVADQDQCIR.H

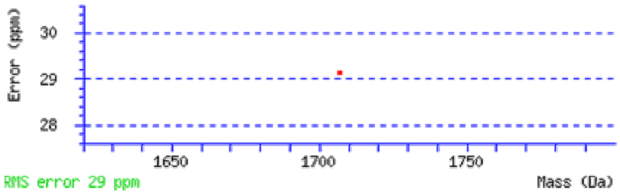

MATRIX SCIENCE MASCOT Search Results

Protein View: RET4_HUMAN

Retinol-binding protein 4 OS=Homo sapiens OX=9606 GN=RBP4 PE=1 SV=3

**Database:**	SwissProt
**Score:**	65
**Monoisotopic mass (M_r):**	23337
**Calculated pI:**	5.76
**Taxonomy:**	Homo sapiens

Sequence similarity is available as an NCBI BLAST search of RET4_HUMAN against nr.

Search parameters

**MS data file:**	DATA.TXT
**Enzyme:**	Trypsin: cuts C-term side of KR unless next residue is P.
**Fixed modifications:**	Carbamidomethyl (C)
**Variable modifications:**	Oxidation (M)

Protein sequence coverage: 10%

Matched peptides shown in ***bold red***.

```
  1 MKWVWALLLL AALGSGRAER DCRVSSFRVK ENFDKARFSG TWYAMAKKDP
 51 EGLFLQDNIV AEFSVDETGQ MSATAKGRVR LLNNWDVCAD MVGTFTDTED
101 PAKFKMKYWG VASFLQKGND DHWIVDTDYD TYAVQYSCRL LNLDGTCADS
151 YSFVFSRDPN GLPPEAQKIV RQRQEELCLA RQYRLIVHNG YCDGRSERNL
201 L
```

Unformatted sequence string: 201 residues (for pasting into other applications).

Sort by ◉ residue number ○ increasing mass ○ decreasing mass
Show ◉ matched peptides only ○ predicted peptides also

Query	Start - End	Observed	Mr(expt)	Mr(calc)	ppm	M	Score	Expect	Rank	U	Peptide
1	118 - 139	2693.1040	2692.0967	2692.1136	-6.28	0	65	1.3e-06	1	U	K.GNDDHWIVDTDYDTYAVQYSCR.L

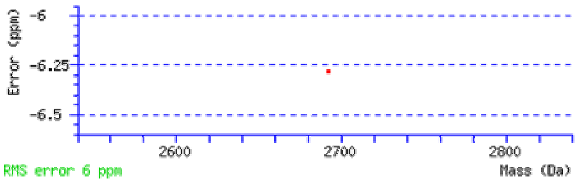

MATRIX SCIENCE MASCOT Search Results

Protein View: HPT_HUMAN

Haptoglobin OS=Homo sapiens OX=9606 GN=HP PE=1 SV=1

**Database:**	SwissProt
**Score:**	70
**Monoisotopic mass (M_r):**	45861
**Calculated pI:**	6.13
**Taxonomy:**	Homo sapiens

Sequence similarity is available as an NCBI BLAST search of HPT_HUMAN against nr.

Search parameters

**MS data file:**	DATA.TXT
**Enzyme:**	Trypsin: cuts C-term side of KR unless next residue is P.
**Fixed modifications:**	Carbamidomethyl (C)
**Variable modifications:**	Oxidation (M)

Protein sequence coverage: 7%

Matched peptides shown in ***bold red***.

1 MSALGAVIAL LLWGQLFAVD SGNDVTDIAD DGCPKPPEIA HGYVEHSVRY
51 QCKNYYKLRT EGDGVYTLND KKQWINK**AVG** **DKLPECEADD** **GCPKPPEIAH**
101 **GYVEHSVR**YQ CKNYYKLRTE GDGVYTLNNE KQWINKAVGD KLPECEAVCG
151 KPKNPANPVQ RILGGHLDAK GSFPWQAKMV SHHNLTTGAT LINEQWLLTT
201 AKNLFLNHSE NATAKDIAPT LTLYVGKKQL VEIEKVVLHP NYSQVDIGLI
251 KLKQKVSVNE RVMPICLPSK DYAEVGRVGY VSGWGRNANF KFTDHLKYVM
301 LPVADQDQCI RHYEGSTVPE KKTPKSPVGV QPILNEHTFC AGMSKYQEDT
351 CYGDAGSAFA VHDLEEDTWY ATGILSFDKS CAVAEYGVYV KVTSIQDWVQ
401 KTIAEN

Unformatted sequence string: 406 residues (for pasting into other applications).

Sort by ◉ residue number ○ increasing mass ○ decreasing mass
Show ◉ matched peptides only ○ predicted peptides also

Query	Start - End	Observed	Mr(expt)	Mr(calc)	ppm	M	Score	Expect	Rank	U	Peptide
1	78 - 108	3432.6189	3431.6116	3431.5874	7.05	1	70	1.2e-06	1	U	K.AVGDKLPECEADDGCPKPPEIAHGYVEHSVR.Y

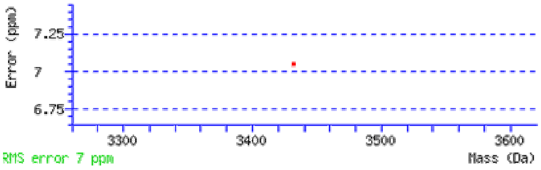

RMS error 7 ppm

MATRIX SCIENCE MASCOT Search Results

Protein View: TTHY_HUMAN

Transthyretin OS=Homo sapiens OX=9606 GN=TTR PE=1 SV=1

**Database:**	SwissProt
**Score:**	189
**Monoisotopic mass (M_r):**	15991
**Calculated pI:**	5.52
**Taxonomy:**	Homo sapiens

Sequence similarity is available as an NCBI BLAST search of TTHY_HUMAN against nr.

Search parameters

**MS data file:**	DATA.TXT
**Enzyme:**	Trypsin: cuts C-term side of KR unless next residue is P.
**Fixed modifications:**	Carbamidomethyl (C)
**Variable modifications:**	Oxidation (M)

Protein sequence coverage: 15%

Matched peptides shown in ***bold red***.

```
  1 MASHRLLLLC LAGLVFVSEA GPTGTGESKC PLMVKVLDAV RGSPAINVAV
 51 HVFRKAADDT WEPFASGKTS ESGELHGLTT EEEFVEGIYK VEIDTKSYWK
101 ALGISPFHEH AEVVFTANDS GPRRYTIAAL LSPYSYSTTA VVTNPKE
```

Unformatted sequence string: 147 residues (for pasting into other applications).

Sort by ◉ residue number ○ increasing mass ○ decreasing mass
Show ◉ matched peptides only ○ predicted peptides also

Query	Start - End	Observed	Mr(expt)	Mr(calc)	ppm	M	Score	Expect	Rank	U	Peptide
1	101 - 123	2451.2000	2450.1927	2450.1979	-2.10	0	189	2.5e-18	1	U	K.ALGISPFHEHAEVVFTANDSGPR.R

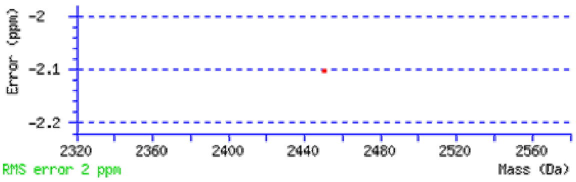

MATRIX SCIENCE MASCOT Search Results

Protein View: HPT_HUMAN

Haptoglobin OS=Homo sapiens OX=9606 GN=HP PE=1 SV=1

**Database:**	SwissProt
**Score:**	91
**Monoisotopic mass (M_r):**	45861
**Calculated pI:**	6.13
**Taxonomy:**	Homo sapiens

Sequence similarity is available as an NCBI BLAST search of HPT_HUMAN against nr.

Search parameters

**MS data file:**	DATA.TXT
**Enzyme:**	Trypsin: cuts C-term side of KR unless next residue is P.
**Fixed modifications:**	Carbamidomethyl (C)
**Variable modifications:**	Oxidation (M)

Protein sequence coverage: 3%

Matched peptides shown in ***bold red***.

```
  1 MSALGAVIAL LLWGQLFAVD SGNDVTDIAD DGCPKPPEIA HGYVEHSVRY
 51 QCKNYYKLRT EGDGVYTLND KKQWINKAVG DKLPECEADD GCPKPPEIAH
101 GYVEHSVRYQ CKNYYKLRTE GDGVYTLNNE KQWINKAVGD KLPECEAVCG
151 KPKNPANPVQ RILGGHLDAK GSFPWQAKMV SHHNLTTGAT LINEQWLLTT
201 AKNLFLNHSE NATAKDIAPT LTLYVGKKQL VEIEKVVLHP NYSQVDIGLI
251 KLKQKVSVNE RVMPICLPSK DYAEVGRVGY VSGWGRNANF KFTDHLKYVM
301 LPVADQDQCI RHYEGSTVPE KKTPKSPVGV QPILNEHTFC AGMSKYQEDT
351 CYGDAGSAFA VHDLEEDTWY ATGILSFDKS CAVAEYGVYV KVTSIQDWVQ
401 KTIAEN
```

Unformatted sequence string: 406 residues (for pasting into other applications).

Sort by ◉ residue number ○ increasing mass ○ decreasing mass
Show ◉ matched peptides only ○ predicted peptides also

Query	Start - End	Observed	Mr(expt)	Mr(calc)	ppm	M	Score	Expect	Rank	U	Peptide
1	117 - 131	1708.9120	1707.9047	1707.8428	36.3	1	91	3.1e-08	1	U	K.LRTEGDGVYTLNNEK.Q

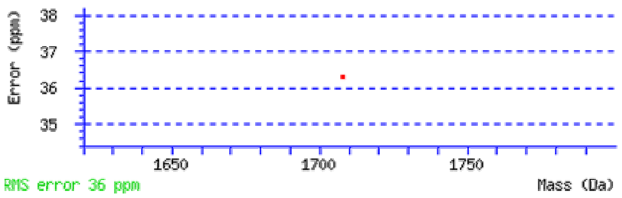

Supplement: Supplementary file 1 — Supplementary information. [file 41598_2020_72104_MOESM1_ESM.pdf]
